# Precision or Paradox? AI‐Driven Adiposity Imaging in Women With Overweight and Obesity: A Systematic Review and Meta‐Analysis

**DOI:** 10.1111/obr.70187

**Published:** 2026-07-08

**Authors:** Asefa Adimasu Taddese, Bjorn T. Tam

**Affiliations:** ^1^ Academy of Wellness and Human Development, Faculty of Arts and Social Sciences Hong Kong Baptist University Hong Kong SAR China; ^2^ Dr. Stephen Hui Research Centre for Physical Recreation and Wellness, Faculty of Arts and Social Sciences Hong Kong Baptist University Hong Kong SAR China

**Keywords:** adipose tissue, artificial intelligence, imaging, precision medicine, women

## Abstract

Artificial intelligence (AI) enables automated, high‐throughput adiposity quantification, offering refined risk stratification for women with overweight and obesity. We systematically reviewed and meta‐analyzed studies evaluating AI‐based segmentation of visceral, subcutaneous, and total fat in adult women populations (BMI: 25–29.9 and ≥ 30 kg/m^2^), searching MEDLINE, CENTRAL, Embase, Scopus, ScienceDirect, and Web of Science from inception to January 15, 2025, in accordance with PRISMA guidelines. Studies were included if they (1) focused on adult women (≥ 18 years) undergoing MRI, CT, or DXA‐based fat quantification; (2) employed automated or conventional radiomics segmentation; and (3) reported quantitative adiposity metrics (volumes or areas). Two reviewers independently extracted data, assessed quality via QUADAS‐2, and synthesized results using inverse‐variance and sample size–weighted random‐effects models. Of 13,098 records screened, 38 studies (12,815 women) met inclusion criteria. AI methods demonstrated high segmentation accuracy, exemplified by 3D‐Unet (Dice score: 0.97 [95% CI: 0.96–0.98]) and > 99% faster processing than manual methods (0.05 vs. 46.5 min/patient; *p* < 0.001). However, the primary finding was extreme between‐study heterogeneity, indicating limited reproducibility of adiposity measurements across studies. Substantial heterogeneity undermined pooled volumetric estimates: subcutaneous fat volume averaged 22,536 cm^3^ (*I* ^2^ = 99.9%), visceral fat 3615 cm^3^ (*I* ^2^ = 100%), and total fat 35,062 cm^3^ (*I* ^2^ = 99.8%), with prediction intervals spanning anatomically implausible ranges. This variability stemmed from inconsistent acquisition parameters, segmentation protocols, and modality‐specific differences, with DXA‐derived subcutaneous fat volumes 5.6‐fold higher than MRI‐derived estimates and CT attenuation thresholds spanning 244 HU (Hounsfield Units). Subgroup analyses indicated 72% of variance arose from technical rather than anthropometric factors. While AI enables highly efficient and accurate segmentation at an individual level, protocol heterogeneity and underrepresentation of diverse populations limit clinical generalizability. Standardized imaging protocols, harmonized analytic frameworks, and inclusive sampling are essential to translate AI's precision into clinically reliable adiposity metrics for women's health.

## 1 Introduction

Obesity prevalence has risen to pandemic levels over the past five decades: worldwide rates more than tripled between 1975 and 2022, with more than 890 million adults and over 1 billion people in total living with obesity [1, 2]. This corresponds to approximately 18.5% of women and 14.0% of men [3], and projections suggest that more than 1.53 billion adults could be living with obesity by 2035 [4, 5]. Women bear a disproportionate metabolic burden [6]: Obesity increases cardiovascular risk, for example, every 5 kg/m^2^ rise in BMI confers a 49% higher hypertension risk (RR: 1.49; 95% CI: 1.40–1.60), and it also increases the incidence of type 2 diabetes and hormone‐driven cancer [7, 8, 9].

Studies suggest that central to these outcomes is the anatomical distribution of adipose tissue. Visceral adipose tissue (VAT) promotes inflammation and insulin resistance via interleukin‐6 and free fatty acid release [10, 11], whereas gluteo‐femoral subcutaneous adipose tissue (SAT) confers metabolic protection through adiponectin‐mediated pathways [12, 13]. A women‐specific focus is further justified by well‐established sex differences in adiposity biology and cardiometabolic risk [14, 15]. Women typically exhibit a gynoid pattern of fat distribution, characterized by greater subcutaneous deposition, whereas men accumulate more VAT, which is more strongly associated with cardiometabolic risk [16, 17]. In addition to these biological differences, anatomical and physiological factors, such as fat compartment geometry and tissue composition, may influence imaging‐derived adiposity measurements and the performance of AI‐based segmentation models. Together, these factors create complex, multilayered imaging phenotypes that require high‐resolution and standardized quantification. However, current methodologies present significant challenges.

Body mass index (BMI) misclassifies up to 17.6% of adults, labeling those with high visceral adiposity as “metabolically healthy” despite adverse risk profiles [18, 19]. Cross‐sectional imaging (CT, MRI, and DXA) enables depot‐specific quantification but exhibits significant technical variability. CT attenuation thresholds for adipose tissue range from −274 to −30 HU depending on scanner and protocol [20], MRI‐based segmentation is subject to interobserver variability [21], and DXA tends to overestimate VAT compared with MRI, particularly in women with android fat patterns [22, 23, 24, 25, 26, 27].

In response, AI‐driven radiomics has emerged as a powerful approach to automate high‐throughput extraction of quantitative imaging biomarkers [28, 29, 30, 31]. Convolutional neural networks (CNNs)—a class of deep learning models designed to recognize patterns in imaging data—have been widely applied to automate adipose tissue segmentation. Among these, U‐Net architectures, which use an encoder–decoder structure to capture both global and fine‐grained image features, are particularly well suited for medical image analysis. These models can accurately delineate visceral and SAT compartments, achieving Dice similarity coefficients (DSC) (a measure of spatial overlap between automated and reference segmentations, where 1.0 indicates perfect agreement) exceeding 0.95 in under 1 min per scan [32, 33]. Radiomic features, such as CT‐derived VAT heterogeneity and MRI‐based SAT texture, have shown strong correlations with insulin resistance (*r* = 0.71) and coronary calcium scoring (AUC = 0.85), suggesting added prognostic value beyond volumetric measures [34, 35, 36, 37, 38, 39].

However, the rapid proliferation of radiomics studies has outpaced efforts to harmonize imaging and analysis protocols. Reviews indicate that repeatability and reproducibility of radiomic features remain limited, with only a subset of texture and morphological metrics demonstrating robust interstudy agreement [40]. The Image Biomarker Standardisation Initiative (IBSI) and related frameworks have called for standardized feature definitions, acquisition parameters, and reporting guidelines to mitigate fragmentation [41]. Without consensus, procedural inconsistencies across scanners (e.g., field strength and slice thickness), segmentation pipelines (threshold‐based vs. deep learning) [42, 43, 44, 45, 46], and feature‐extraction toolkits compromise cross‐study comparability [47].

Given the accelerating adoption of AI alongside persisting reproducibility challenges, there is an urgent need to synthesize existing evidence. We define the current state as a “precision paradox,” referring to the discrepancy between the high technical accuracy of AI‐based adipose tissue segmentation and its limited clinical reliability and cross‐study comparability. While AI models can achieve near‐perfect segmentation performance (e.g., high DSC), variability in imaging acquisition parameters, segmentation pipelines, and feature extraction methods introduces substantial inconsistency in derived adiposity metrics. This paradox therefore reflects both methodological heterogeneity and a clinical gap in the reliable quantification of adiposity. Although AI‐based imaging holds promise for standardized, automated, and high‐resolution assessment of fat depots, this potential remains constrained by the absence of harmonized protocols and validation frameworks. A consolidated, women‐specific meta‐analysis is therefore needed to quantify the extent to which methodological variability influences adiposity estimates and to identify points of convergence for protocol standardization. Accordingly, this review aims to (1) quantify the confounding effects of methodological heterogeneity on AI‐derived fat measurements in women; (2) compare AI segmentation performance with conventional methods in women cohorts; and (3) propose standardized frameworks to resolve the precision paradox in adiposity imaging.

## 2 Methods and Materials

### 2.1 Search Strategy and Selection Criteria

To comprehensively evaluate the role of radiomics in adiposity assessment among women with overweight and obesity, we conducted a systematic review in accordance with PRISMA‐2020 guidelines [48]. The completed PRISMA checklist is provided in Table S1.

A comprehensive search was performed across six electronic databases—MEDLINE (via PubMed), CENTRAL (via Cochrane Library), Embase (via Ovid), Scopus, ScienceDirect, and Web of Science Core Collection, from their inception to January 15, 2025. The search strategy combined controlled vocabulary terms (e.g., MeSH and Emtree) with free‐text keywords such as “radiomic profile,” “adiposity,” “obesity risk,” and “women,” using Boolean operators (AND/OR) to balance comprehensive retrieval of relevant studies (sensitivity) with exclusion of irrelevant records (specificity). Iterative refinement of search terms was conducted based on keywords identified during initial screening. Grey literature was also systematically searched, including conference proceedings, preprint servers, and technical reports. Detailed grey literature sources and search strategies are provided in Tables S2 and S3.

Following deduplication in EndNote 20.2, two reviewers independently screened titles and abstracts using Covidence systematic review software (Veritas Health Innovation, Melbourne, Australia) to facilitate structured blinded review [49]. A pilot calibration exercise was conducted prior to full screening. Studies were included if they (1) focused on adult women (≥ 18 years) undergoing MRI‐, CT‐, or DXA‐based fat quantification; (2) employed automated or conventional radiomics segmentation; and (3) reported quantitative adiposity metrics (volumes or areas). We excluded only male‐mixed‐sex cohorts without sex‐specific data, pediatric or adolescent populations, case reports, reviews, editorials, and conference abstracts lacking original data. Discrepancies were resolved through discussion, with consultation of a third independent reviewer when necessary.

### 2.2 Data Extraction and Quality Assessment

Two reviewers independently extracted data using a standardized, pilot‐tested extraction form capturing study characteristics, participant demographics, imaging parameters, segmentation methods, and funding/conflict‐of‐interest statements. Methodological quality and risk of bias were assessed using the QUADAS‐2 tool [50], across the domains of patient selection, index test (radiomics model validation), reference standard (accuracy of ground‐truth measurements), and flow/timing (protocol consistency). Studies without independent blinded assessment or dual‐reader consensus for ground‐truth segmentation were classified as high risk in the reference standard domain, given the potential for measurement bias. Agreement analyses comparing automated and conventional segmentation approaches are shown in Figure S1.

### 2.3 Data Analysis

Statistical analyses were conducted in R version 4.1.2 using the meta, lme4, and ggplot2 packages. Adiposity metrics were synthesized through two complementary approaches: (1) sample size–weighted pooled means, allowing larger studies to contribute proportionally more to the overall estimate, and (2) inverse‐variance weighted random‐effects models (DerSimonian–Laird estimator) to account for between‐study clinical and methodological heterogeneity.

Given the known methodological differences between imaging modalities, adiposity measurements derived from MRI, CT, and DXA were not assumed to be directly equivalent. These modalities rely on distinct physical principles—MRI on proton signal properties, CT on X‐ray attenuation, and DXA on differential photon absorption—and may therefore yield systematically different estimates of adipose tissue depots. Accordingly, pooled estimates across modalities were interpreted with caution and were intended to capture intermodality variability rather than to provide a single, clinically interchangeable measure of adiposity.

Heterogeneity was quantified using the *I* ^2^ statistic (with values ≥ 75% indicating substantial heterogeneity) and tau‐squared (τ^2^). Subgroup analyses stratified results by imaging modality, anthropometric variables (weight and waist circumference), and segmentation type, with imaging modality treated as a primary source of heterogeneity. Sensitivity analyses included leave‐one‐out meta‐analyses, trim‐and‐fill adjustments for publication bias, and exclusion of outliers.

The study protocol was registered with PROSPERO (CRD420251026787) and adhered to ethical standards by utilizing aggregated, anonymized data from published studies.

## 3 Results

**FIGURE 1:**
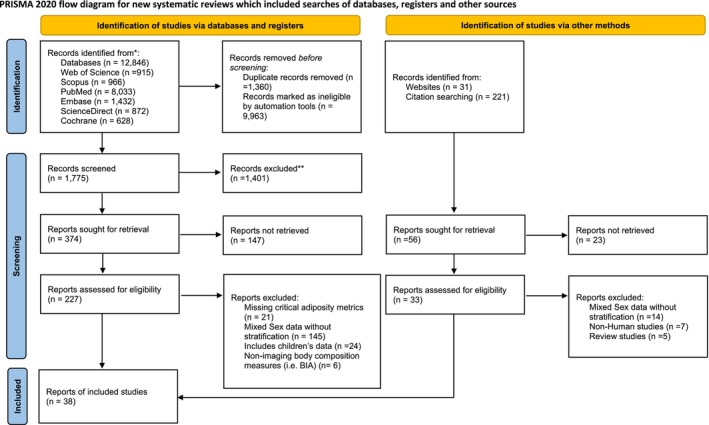
PRISMA 2020 flow diagram illustrates the systematic literature search process, detailing study identification through databases/registers and additional methods, screening procedures, and final study inclusion for meta‐analysis.

Following PRISMA‐2020 guidelines, 13,098 records were identified, of which 11,323 were excluded during prescreening (duplicates and automation). The remaining 1775 records underwent title/abstract screening, with 1401 excluded. Of 374 full‐text articles sought, 260 were assessed for eligibility; 222 were excluded due to missing adiposity metrics (*n* = 21), mixed‐sex data (*n* = 159), pediatric populations (*n* = 24), nonhuman studies (*n* = 7), reviews (*n* = 5), or non‐imaging measures (*n* = 6). Ultimately, 38 studies (1990–2025) involving 12,815 women living with overweight and obesity were included in the meta‐analysis (Figure 1).

Studies predominantly originated from high‐income regions (92.1%, *n* = 35/38) (as classified by the World Bank) [26, 27, 51, 52, 53, 54, 55, 56, 57, 58, 59, 60, 61, 62, 63, 64, 65, 66, 67, 68, 69, 70, 71, 72, 73, 74, 75, 76, 77, 78, 79, 80, 81, 82, 83, 84], with underrepresentation of African (2.6%, *n* = 1/38) [85] and Asian (5.26%, *n* = 2/38) [86, 87] cohorts. Participant ethnicity was inconsistently reported across studies; therefore, geographic location was used as a proxy for population characteristics. Pooled participant characteristics included a mean age of 51.0 years (95% CI: 50.1–54.4), BMI of 32.0 kg/m^2^ (95% CI: 30.7–33.4), and elevated adiposity indices (body fat percentage: 39% [95% CI: 36–42]; relative fat mass: 8.8 kg/m^2^ [95% CI: 8.1–9.5]) (Table 1).

**TABLE 1:** Participant characteristics and adiposity measures in AI‐driven adiposity imaging in women with obesity and overweight.

First author, year, country	Total number of women (images/scans)	Number of women with obesity/overweight	BMI thresholds in kg/m^2^ (obesity classification)	Obesity/overweight classification	Mean BMI (kg/m^2^) [SD]	Women with additional comorbidities (*N*)	Age, years (mean ± SD)	Mean weight (kg) [SD]	Mean height (m) [SD]	Mean waist (m) [SD]	Mean hip (m) [SD]	Adiposity indices (BAI/FMI/BFP)
Schneider et al. (2023), Germany	331 (12,422)	331	≥ 35	Obesity Class II (severe)	NR	NR	NR	NR	NR	NR	NR	NR
Bea et al. (2022), United States	69 (173)	21	≥ 30	Obesity Class I (moderate)	27.3 ± 5.4	NR	70.5 ± 5.8	NR	NR	0.79 ± 0.12	NR	FMI: 42.77
Hexsel et al. (2022), Brazil	133 (133)	46	≥ 25	Overweight	26.63 ± 1.3	Dermatologic (133)	41.7 ± 15.9	NR	NR	NR	NR	BFP: 34.43
Flehr et al. (2022), Sweden	38 (110)	14	≥ 25	Overweight	25.2 ± 4.0	Musculoskeletal, metabolic, CVD (24)	75.9 ± 3.1	66.2 ± 7.8	1.62 ± 0.06	NR	NR	BFP: 42.18
Froelich et al. (2020), Germany	116 (NR)	58	≥ 30	Obesity Class I (moderate)	25.1 ± 5.3	Metabolic (72)	35 ± 4	69.9 ± 14.8	1.67 ± 0.06	0.81 ± 0.11	1.00 ± 0.11	BAI: 28.34/FMI: 8.14/BFP: 33.49
Jayawarde et al. (2020), United States	45 (45)	25	≥ 25	Overweight	25.7 ± 4.2	Metabolic (7)	52.9 ± 2.6	NR	NR	0.83 ± 0.11	NR	NR
Klingensmith et al. (2019), United States	10 (635)	10	≥ 30	Obesity Class I (moderate)	35 ± 17.5	NR	27.5 ± 4.3	NR	NR	NR	NR	NR
Middleton et al. (2017), United States	18 (13,140)	18			27.46 ± 6.9	Metabolic (18)	52.9 ± 17.1	NR	NR	NR	NR	BFP: 50.57
Karampatos et al. (2016), Canada	21 (210)	13	≥ 30	Obesity Class I (moderate)	36.5 ± 9.0	Metabolic (12)	72 ± 3.5	NR	NR	NR	NR	BFP: 52.32
Silver et al. (2013), United States	12 (2448)	12	≥ 30	Obesity Class I (moderate)	34.3 ± 2.8	NR	36.7 ± 9.0	88.4 ± 12.1	1.60 ± 0.08	0.98 ± 0.08	1.16 ± 0.08	BAI: 15.59/BFP: 38.56
Schautz et al. (2011), Germany	97 (97)	47	≥ 28	Overweight	29.6 ± 7.1	NR	31.2 ± 6.7	84.9 ± 22.0	1.69 ± 0.07	0.95 ± 0.18	NR	BAI: 12.25/BFP: 35.86
Piché et al. (2008), Canada	113 (226)	43	≥ 30	Obesity Class I (moderate)	28.4 ± 5.1	NR	56.9 ± 4.4	29.3 ± 10.4	NR	0.91 ± 0.13	1.07 ± 0.11	BFP: 46.45
Ross et al. (2002), Canada	40 (1800)	40	≥ 25	Overweight	32.3 ± 3.3	NR	42 ± 7	NR	NR	0.98 ± 0.08	NR	NR
Gronemeyer et al. (2000), United States	16 (496)	16	≥ 30	Obesity Class I (moderate)		NR	32 ± 6.3	NR	NR	NR	NR	BFP: 53.08
Busetto et al. (2000), Italy	6 (246)	6	≥ 40	Obesity Class III (very severe)	42.6 ± 1.1	NR	NR	107 ± 5.3	NR	1.21 ± 0.07	1.25 ± 0.08	BFP: 32.90
Thomas et al. (1998), England	67 (67)	23	≥ 30	Obesity Class I (moderate)	31.92 ± 9.4	Genetic (13)	29.4 ± 6.7	92.3 ± 9.9	1.63 ± 0.03	NR	NR	BFP: 30.01
Schapira et al. (1994), United States	80 (NR)	23	≥ 25	Overweight	23.87 ± 5.5	Cancer (40)	47.1 ± 1.6	65.5 ± 1.7	NR	0.79 ± 0.02	NR	BFP: 32.31
Sohlstrom et al. (1993), Sweden	25 (750)	5	≥ 25	Overweight	22.4 ± 3.2	NR	30 ± 5	61.2 ± 9.4	NR	NR	NR	BFP: 45.06
Ross et al. (1993), Canada	15 (615)	15	≥ 30	Obesity Class I (moderate)	36.3 ± 7.1	NR	35 ± 9.8	98.6 ± 18.4	1.65 ± 0.05	1.15 ± 0.19	1.26 ± 0.16	BFP: 39.97
Fowler et al. (1991), Scotland	14 (196)	7	≥ 30	Obesity Class I (moderate)	31.1 ± 3.3	NR	37 ± 10	57.3 ± 4.4	1.66 ± 0.06	NR	NR	NR
Armao et al. (2006), United States	5 (140)	5	≥ 30	Obesity Class I (moderate)		NR	NR	NR	NR	NR	NR	BFP: 38.28
Davidson et al. (2020), South Africa	132 (132)	92	≥ 30	Obesity Class I (moderate)	36.4 ± 1.9	NR	57 ± 10	62	NR	0.85 ± 0.09	0.55 ± 0.02	BAI: 36.19/FMI: 34.11/BFP: 37.59
Waduud et al. (2017), Scotland	15 (450)	7	≥ 25	Overweight	24.9 ± 2.8	Cancer (15)	48.7 ± 7.1	67.5 ± 5.4	1.65 ± 0.07	0.93 ± 0.05	NR	BFP: 49.24
Bi et al. (2015), United States	229 (NR)	229	≥ 30	Obesity Class I (moderate)	36.2 ± 4.15	Metabolic (167)	39.2 ± 8.7	98.1 ± 15.0	1.64 ± 0.09	1.06 ± 0.11	1.20 ± 0.09	BAI: 39.14/FMI: 16.34/BFP: 44.54
Bredella et al. (2010), United States	57 (NR)	34	≥ 25	Overweight	34.1 ± 4.7	Psychiatric (39)	34.8 ± 7.1	53.9 ± 7.6	1.26 ± 0.03	NR	NR	FMI: 11.34/BFP: 35.60
Valentinitsch et al. (2013), United States	28 (840)	16	≥ 30	Obesity Class I (moderate)	27.5 ± 4.2	Metabolic (16)	65 ± 5.8	NR	NR	NR	NR	BFP: 41.83
Varady et al. (2007), Canada	31 (NR)	31	≥ 25	Overweight	26.9 ± 3.1	NR	50.1 ± 8.2	70.4 ± 9.9	1.62 ± 0.05	NR	NR	FMI: 8.54
Kway et al. (2023), England	389 (NR)	69	≥ 30	Obesity Class I (moderate)		NR	31.4 ± 3.9	NR	NR	NR	NR	BFP: 33.26
Haueise et al. (2023), Germany	5433 (NR)	3214	≥ 25	Overweight	26.2 ± 5.2	NR	51.7 ± 11.3	71.2 ± 14.4	1.65 ± 0.07	0.86 ± 0.13	NR	BFP: 48.37
Linder et al. (2019), Germany	13 (650)	13	≥ 30	Obesity Class I (moderate)	34.9 ± 5.0	NR	49 ± 3	NR	NR	NR	NR	NR
Maislin et al. (2012), United States	129 (NR)	71	≥ 30	Obesity Class I (moderate)	33.7 ± 6.0	Respiratory (129)	58.8 ± 9.1	NR	NR	1.06 ± 0.14	NR	BFP: 43.40
Schwenzer et al. (2010), Germany	227 (22,700)	227	≥ 27	Overweight	29.4 ± 5.4	NR	45.1 ± 11.4	81 ± 15.4	1.66 ± 0.06	NR	NR	BFP: 37.25
Mourtzakis et al. (2008), Canada	21 (42)	4	≥ 30	Obesity Class I (moderate)	26.9 ± 8.2	Cancer (21)	61 ± 11	NR	NR	NR	NR	NR
Positano et al. (2004), Italy	20 (640)	10	≥ 30	Obesity Class I (moderate)		Metabolic (20)	NR	NR	NR	NR	NR	BFP: 45.72
Kim et al. (2019), South Korea	445 (445)	27	≥ 30	Obesity Class I (moderate)	23.6 ± 3.1	Cancer (234)	52.6 ± 10.1	NR	NR	NR	NR	BFP: 32.6 ± 10.5

*Note:* BMI thresholds refer to study‐specific inclusion criteria for overweight/obesity (e.g., ≥ 25, ≥ 30, and ≥ 35 kg/m^2^), whereas reported mean BMI values represent pooled participant averages from the full study sample. Comorbidity categories were classified as follows: metabolic (type 2 diabetes mellitus, hypertension, hypercholesterolemia, NAFLD, metabolic syndrome, and gestational diabetes), cancer (breast, lung, and colorectal), genetic (Prader–Willi syndrome), cardiovascular (CVD), musculoskeletal (osteoporosis), psychiatric (anorexia nervosa), dermatologic (cellulite and skin laxity), and respiratory (obstructive sleep apnea). Waist and hip circumferences are reported in meters (m); weight is reported in kilograms (kg); adiposity area and volume measurements are reported according to the original studies. NR indicates data not available or not reported in the original publication.

Abbreviations: BAI = body adiposity index; BFP = body fat percentage; BMI = body mass index; CVD = cardiovascular disease; FMI = fat mass index; NAFLD = nonalcoholic fatty liver disease; NR = not reported; SD = standard deviation.

Our synthesis of 38 studies reveals that AI‐driven adiposity imaging in women with overweight and obesity combines remarkable technical advances with persistent methodological inconsistencies. Schneider et al. [27] demonstrated that fully convolutional networks achieved high segmentation precision for SAT with a DSC of 0.954 and for VAT with a DSC of 0.889. Additionally, a two‐step CNN pipeline reduced pixel‐level misclassification by 12% in abdominal CT scans [83]. Similarly, Kway et al. [65] reported that a 3D‐Unet model delivered Dice coefficients above 0.95 for SAT and VAT subdepot segmentation, although manual refinement was necessary in anatomically complex regions such as ovarian fat. Maislin et al. [68] validated that a single L2–L3 MRI slice could predict whole‐abdomen VAT with an *R* ^2^ of 0.96 in women, a finding that contrasts with other approaches requiring full volumetric data. Davidson et al. [85] found a strong correlation (*r* = 0.93) between CT and DXA measurements using cross‐modality equations (VAT‐CT = 0.03 × VAT‐DXA + 0.3), with race‐specific calibration required to maintain accuracy. Furthermore, longitudinal MRI–CT comparisons accurately identified sex‐specific VAT/SAT ratio trajectories [66].

Despite these technical gains, methodological heterogeneity remains a critical barrier. Across studies, CT attenuation thresholds ranged from −274 to −30 HU, with only 18% applying sex‐specific thresholds (e.g., −140 to −40 HU for women vs. −190 to −30 HU for men) [62, 67]. MRI slice thickness varied widely, from 1.8 mm [71] in breast imaging protocols to 20 mm in whole‐body scans [79]. Low‐dose CT protocols, while reducing radiation exposure to approximately 1.5 mSv, introduced a 12% inflation in VAT estimation error [66]. Definitions of adipose depots varied significantly. Some studies measured only pericardial fat, yielding an AUC of 0.78 for coronary outcomes [62], whereas others included mediastinal fat, resulting in up to 25% variability in predictive performance [87]. Moreover, race‐dependent metabolic risk thresholds were evident [53], with DXA‐VAT cutoffs diverging between African American (1320 cm^3^) and European American women (1713 cm^3^) [85]. Geographic and demographic representation was skewed, with 94% of cohorts comprising European populations [52, 53]. Finally, reporting standards were inconsistent: Only 23% of studies reported interobserver variability, which was under 2% for AI methods compared to 5%–10% for manual segmentation (Table 2). These findings highlight the potential of AI‐driven imaging to enhance adiposity measurements but underscore the need for standardized protocols and diverse cohorts to ensure accuracy and generalizability. A summary of imaging modalities and protocols is provided in Table S4.

**TABLE 2:** Summary of studies on adipose tissue quantification using imaging modalities: methodologies, validation, and key findings.

Study (author, year, country)	Imaging modality	Region of interest	Fat measurement metric	Segmentation method	Validation	Analysis software	Adipose depot	Major findings	Overall risk of bias
Schneider et al. (2023), Germany	MRI	Full abdominopelvic region	Signal intensity differences (dual‐echo sequences)	UNet variants (DenseUNet, CD‐FNet)	Semiautomated corrections (DicomFlex)	Custom	Total abdominal fat	Dice coefficients: 0.954 (SAT), 0.889 (VAT). Pearson correlations: 0.999 (SAT), 0.997 (VAT). 90% faster processing vs. manual methods.	Low
Bea et al. (2022), United States	DXA	Lumbar spine (L4‐L5)	Percent fat fraction (%)	Hologic APEX 4.0 algorithm	Cross‐modality (MRI vs. DXA)	Hologic APEX 4.0	SAT, VAT	DXA‐VAT vs. MRI: *r* = 0.90 (VAT), *r* = 0.92 (SAT). DXA overestimated SAT by −85.16 to −118.66 cm^2^ (QDR4500). Older DXA models showed greater bias (−30.71 cm^2^).	Low
Hexsel et al. (2022), Brazil	MRI	Pelvis (gluteal region)	Adipose thickness (mm)	Manual delineation	Expert review	DICOM Horos	SAT, fat lobules	SAT thickness correlated with BMI (*r* = 0.64). Strongest correlation in ages 15–30 (*r* = 0.76) and 6–60 (*r* = 0.75). SAT decreased with aging in low‐BMI groups.	High
Flehr et al. (2022), Sweden	HR‐pQCT	Distal/proximal tibia	Bone marrow radiodensity (−80 to +2000 HU)	MATLAB‐based voxel peeling	Software consistency checks	MATLAB	Bone marrow fat fraction	HR‐pQCT explained 76% of MRI‐derived bone marrow fat variation (*p* < 0.001) using ≥ 4 peeled voxel layers.	Medium
Froelich et al. (2020), Germany	MRI	Whole body	Fat fraction (mDixon fat‐water separation)	Slice‐O‐Matic 4.3	Cross‐modality (MRI vs. BIA)	SliceOmatic 4.3	VAT, SAT, total body fat	MRI total fat mass (FM) correlated with BIA (β = 0.583, *p* < 0.001). Deviations > 5 kg (FM) and > 1 kg (VAT).	Low
Jayawarde et al. (2020), United States	CT	Heart (epicardial/intrathoracic)	HU thresholds (−190 to −30)	QFAT 2.0 Software	Blinded repeated readings	QFAT 2.0	Epicardial fat volume (EFV)	Intra‐/interobserver ICC = 0.99 for EFV. Bland–Altman mean difference: 0.90 cm^3^ (intra), −1.8 cm^3^ (inter).	High
Priya and Sudha (2019), India	CT	Heart (epicardial/mediastinal)	Threshold (−100 HU)	Adaptive Fruitfly + GWO Neural Network	Manual segmentation comparison	Custom software	EFV, mediastinal fat volume (MFV)	AI‐driven segmentation improved mediastinal fat classification accuracy by 26% vs. manual methods.	Medium
Klingensmith et al. (2019), United States	MRI	Thoracic/abdominal	Binary fat/water masks (grayscale clustering)	K‐Means Clustering	Repeated scans	Python 3.5.4 (SciPy, NumPy)	SAT, VAT, cardiac adipose	*R* ^2^ = 0.80 (CAT), 0.99 (SAT/VAT) vs. manual CSA. Supports longitudinal obesity studies.	Low
Petridou et al. (2017), England	MRI	Breast tissue	Calibrated fat volume fraction (0%–100%)	AMRA Profiler	BI‐RADS mammography validation	AMRA segmentation software	Total breast fat percentage	Pre‐/post‐contrast breast density correlation: rho = 0.98–0.99. BI‐RADS vs. MRI: rho = 0.73–0.75.	High
Wang et al. (2017), United States	CT	Abdominal	HU thresholds (−140 to −40)	Two‐step CNN	Internal (CAD datasets)	Custom CNN	Total abdominal adipose	CNN accuracy: 95.8% (slice selection), 96.8% (fat segmentation). Automated SFA/VFA validated.	Medium
Middleton et al. (2017), United States	MRS	Whole body (skull base to knees)	Hepatic PDFF (IDEAL‐IQ)	AMRA Profiler	Manual corrections with Slice‐O‐Matic	SliceOmatic	SCAT, VAT, TAT, Thigh Muscle Volume	Intraclass correlation coefficients: 0.996–0.998 (adipose/muscle). Hepatic PDFF CV ≤ 7.3%.	Low
Karampatos et al. (2016), Canada	MRI	Lower leg (nondominant tibia)	Mathematical morphology and region‐growing	Slice‐O‐Matic 4.3	Radiologist verification	SliceOmatic	SAT, IntraMAT, InterMAT, Total Muscle	IntraMAT predicted frailty (OR = 2.5). Intra‐rater ICC = 0.991, inter‐rater ICC = 0.983.	Medium
Silver et al. (2013), United States	MRI	Whole body (trunk‐focused)	Fat signal fraction > 50% (volume‐to‐mass conversion)	Berglund's 3‐Point Dixon Algorithm	DXA‐derived validation	Custom fat‐water separation software	SAT, VAT, GBAT, GBLST	MRI vs. DXA concordance: 0.978 (GBAT), 0.802 (TTAT). DXA overestimated lean soft tissue.	High
Varady et al. (2007), Canada	MRI	Whole body (upper + lower body)	Fat mass (kg), percent fat mass (%)	Slice‐by‐slice tissue tagging (color coding)	Compared BIA estimates to MRI (reference method)	Slice‐O‐Matic v4.2	Fat mass (kg), percent fat mass (%)	Handheld BIA (Omron BF302) underestimated fat mass by 2.3 ± 3.3 kg and percent fat by 5.6% ± 3.9%; BIA reproducible (TE for fat mass 0.06 ± 0.07 kg) but not valid against MRI in overweight women	Medium
Schautz et al. (2011), Germany	MRI	Wrist to ankle	BrAT delineation	Slice‐O‐Matic 4.3	Manual review	SliceOmatic	BrAT, SAT, VAT	BrAT volume decreased by 20% post‐weight loss. No link to cardiometabolic risk. SATtrunk and VAT predicted risk (*p* < 0.05).	Medium
Piché et al. (2008), Canada	CT	Abdominal (L4‐L5), Midthigh	HU thresholds (−190 to −30)	Manual delineation	Graph pen validation	Manual software	TAT, VAT, SAT, mid‐thigh Adipose	VAT area at L4‐L5 predicted cardiovascular events (HR = 1.9). Mid‐thigh fat inversely linked to metabolic risk.	High
Ross et al. (2002), Canada	MRI/CT	Whole body (MRI); L4–L5 (CT)	MRI: Fat volume (0.92‐kg/L density); CT: HU thresholds (−190 to −30)	Voxel intensity (MRI); HU thresholds (CT)	Manual segmentation	Slice‐O‐Matic	TAT, SAT, VAT, Non‐abdominal fat	VAT correlated with insulin resistance (*r* = −0.42, *p* < 0.01). High VAT groups had lower glucose disposal rates (*p* < 0.05).	High
Gronemeyer et al. (2000), United States	MRI	Whole abdomen	Signal intensity thresholds	Signal Intensity Thresholding	Visual validation	Photoshop	SAT, VAT, TAT	Automated umbilicus‐level MRI correlated with manual whole‐abdomen analysis (*r* ^2^ = 0.958 SAT, 0.753 VAT). 6 min vs. 2‐h processing time.	Low
Busetto et al. (2000), Italy	MRI	Whole body (abdominal/gluteo‐femoral)	Grayscale intensity threshold (130/256)	Intensity Thresholding	Reproducibility testing (CV < 2%)	NIH Image	TAT, SAT, VAT, SAT‐gluteo‐femoral	VAT reduced by 1.0 L (0–8 weeks, *p* < 0.05). Gluteo‐femoral SAT protected against metabolic syndrome (OR = 0.6).	High
Thomas et al. (1998), England	MRI	Whole body (fingertips to toes)	Histogram analysis + contour‐following	Histogram Analysis	BIA validation	Custom knowledge‐based software	TAT, SAT, VAT	MRI vs. BIA correlation: *r* = 0.93 (overweight), *r* = 0.90 (obese). Weak correlation in lean groups (*r* = 0.54).	Medium
Schapira et al. (1994), United States	CT	L4 vertebral level	HU thresholds (−30 to −200)	ROI‐based segmentation	Manual validation	Manual software	TFA, VFA, SFA	Visceral fat area (VFA) predicted breast cancer risk (OR = 9.5 for VT ratio > 0.24).	High
Sohlstrom et al. (1993), Sweden	MRI	Whole body	Adipose volume (0.9225‐kg/L density)	Manual delineation	Underwater weighing	MIS 100	TAT, SAT, NSAT	MRI fat mass correlated with underwater weighing (*r* = 0.94). Subcutaneous fat: 74.2% of total adipose tissue.	High
Ross et al. (1993), Canada	MRI	Whole body	Pixel intensity threshold > 110	Threshold‐Based Segmentation	Manual segmentation	In‐house software	TAT, SAT, VAT	Threshold‐based segmentation reduced analysis time by 60%. Subcutaneous fat: 92.3% of total adipose tissue.	High
Fowler et al. (1991), Scotland	MRI	Whole body (anatomical landmarks)	Truncated cone model (78.3%–83.2% lipid correction)	Interactive Thresholding	UWW, BIA, body water dilution	Custom‐developed software	TAT, SAT, Non‐subcutaneous fat	MRI predicted body fat % with residual SD = 2.9%, outperforming skinfolds, BIA, and DXA.	Medium
Armao et al. (2006), United States	MRI	Abdomen/pelvis	Confidence‐connected region growing	Region Growing	Manual segmentation comparison	NLM Insight Toolkit	TAT, SAT, VAT	Automated VAT segmentation: 5 min vs. 1‐h manual. Reproducibility: ±5% variability.	High
Davidson et al. (2020), South Africa	DXA/CT	L4–L5 (CT); Android region (DXA)	DXA: Proprietary Hologic algorithm; CT: HU thresholds	Proprietary Algorithm (Hologic)	Cross‐modality comparison	Hologic Discovery W, Vitrea Core	VAT, SAT	DXA‐VAT vs. CT: *r* = 0.93 (SEE = 16 cm^2^). Outperformed anthropometric models (*r* = 0.79).	Low
Waduud et al. (2017), Scotland	CT/MRI	L3 vertebra	CT: HU thresholds (−190 to −30); MRI: Signal intensity	Threshold‐Based Segmentation	Manual vs. semiautomated comparison	ImageJ	TAA, SAT, VAT, EAF, IAF	Interchangeable CT/MRI models (Pearson's *R* = 0.625–0.903). L3 VAT predicted postoperative complications (OR = 2.4).	Medium
Bi et al. (2015), United States	DXA	Android region	VAT mass (0.94‐g/mL conversion)	CoreScan Algorithm	Not reported	enCore software	VAT mass/volume, total abdominal fat	DXA‐VAT predicted MetSx (AUC = 0.749). Race‐specific thresholds: 1320 cm^3^ (AA), 1713 cm^3^ (EA).	High
Bredella et al. (2010), United States	CT/DXA	L4 (abdomen), mid‐thigh	CT: HU thresholds (−50 to −250); DXA: CoreScan algorithm	CoreScan Algorithm	Not reported	Alice, Accuview	VAT, SAT, thigh fat/muscle	DXA underestimated VAT by 15% vs. CT in obese women. Strong correlations (*r* = 0.77–0.95).	Medium
Valentinitsch et al. (2013), United States	MRI	Calf/thigh	Fat fraction (six‐echo SPGR)	K‐Means Clustering	Ground‐truth comparison	MATLAB	SAT, IMAT	Automatic IMAT segmentation: *R* ^2^ = 0.96 (thigh), 0.92 (calf). High error in severe muscle fat infiltration.	Medium
Kway et al. (2023), England	MRI	Abdomen (L1‐L5)	Volumetric fat depots	3D‐UNet + manual refinement	Ground‐truth comparison	ITK‐SNAP, PyTorch	SAT (superficial/deep), Intra‐abdominal fat	3D‐UNet Dice scores: 98.3% (SSAT), 95.9% (PSAT). Validated in external cohort (*N* = 50).	Low
Haueise et al. (2023), Germany	MRI	Body trunk	VAT/SAT spatial distribution	3D nnU‐Net	Manual annotations	nnU‐Net, ITK‐SNAP	VAT (retroperitoneal/paraspinal), SAT	nnU‐Net Dice > 0.94. Processing time: 15 s vs. 3–4‐h manual. VAT higher in males, SAT in females.	Low
Linder et al. (2019), Germany	MRI	Abdomen (diaphragm to pelvis)	ASAT/VAT volumes	Dicomflex	Bland–Altman analysis	Custom Matlab‐based Dicomflex	ASAT, VAT	Half‐body MRI underestimated VAT by 8%. Strong correlations (*R* ^2^ > 0.97) for ASAT/VAT.	Low
Maislin et al. (2012), United States	MRI	Abdominal compartment	Volumetric analysis (Amira software)	Region Growing + Threshold	Manual validation	Amira 4.1.2	SAT, VAT	L2‐L3 VAT area predicted total VAT volume (*r* = 0.96). Better than L4‐L5 (*r* = 0.83).	Low
Schwenzer et al. (2010), Germany	MRI	Whole body	In‐house MATLAB tool	MATLAB‐based segmentation	Whole‐body validation	MATLAB	TAT, VAT, SCAT, NVAT	Umbilical VAT(u) predicted total VAT: *r* = 0.93 (women), 0.87 (men). Outperformed BMI (*r* = 0.56–0.71).	Low
Mourtzakis et al. (2008), Canada	CT	L3 vertebra	HU thresholds (−50 to −150 for VAT; −190 to −30 for SAT)	Slice‐O‐Matic	DXA/BIA comparison	SliceOmatic	VAT, SAT, intramuscular fat, TAT	CT predicted whole‐body fat (*r* = 0.86–0.94). Detected fat‐free mass changes missed by BIA.	Low
Positano et al. (2004), Italy	MRI	Abdomen (diaphragm to pelvis)	Fuzzy C‐means tissue classification	Fuzzy clustering	Gaussian curve fitting	IDL 6.0	VAT, SAT, VAT/SAT ratio	Unsupervised vs. manual: *r* = 0.9917 (SAT), 0.9601 (VAT). Overestimated SAT due to arm inclusion.	High
Kim et al. (2019), South Korea	CT	Umbilicus/L4	HU thresholds (−50 to −250)	Manual segmentation	Intraclass correlation (ICC: 0.98)	GE Advantage Windows	VAT, SAT, TAT, VAT/SAT ratio	Visceral obesity increased breast cancer risk in postmenopausal women (OR = 1.50). Higher VAT/SAT linked to negative PR/ER status.	High

*Note:* Dice coefficients, Pearson correlations, and *R* ^2^ values closer to 1.0 indicate stronger agreement or segmentation performance. HU thresholds refer to attenuation ranges used for adipose tissue identification in CT imaging. Risk of bias (ROB) was assessed using the QUADAS‐2 framework across four domains: patient selection (P), index test (I), reference dtandard (R), and flow and timing (FT). Overall, ROB was rated as low when all domains were judged low risk, high when ≥ 1 domain was judged high risk, and unclear/medium when ≥ 1 domain was judged unclear without any high‐risk domain.

Abbreviations: AA = African American; ASAT = abdominal subcutaneous adipose tissue; AUC = area under the curve; BIA = bioelectrical impedance analysis; BI‐RADS = Breast Imaging Reporting and Data System; BrAT = brown adipose tissue; CAD = computer‐aided diagnosis; CAT = cardiac adipose tissue; CNN = convolutional neural network; CT = computed tomography; CV = coefficient of variation; DXA = dual‐energy X‐ray absorptiometry; EA = European American; EAF = epicardial adipose fat; EFV = epicardial fat volume; ER = estrogen receptor; GBAT = gross body adipose tissue; GBLST = gross body lean soft tissue; HR = hazard ratio; HR‐pQCT = high‐resolution peripheral quantitative computed tomography; HU = Hounsfield units; IAF = intra‐abdominal fat; ICC = intraclass correlation coefficient; InterMAT = intermuscular adipose tissue; IntraMAT = intramuscular adipose tissue; MetSx = metabolic syndrome; MFV = mediastinal fat volume; MRI = magnetic resonance imaging; MRS = magnetic resonance spectroscopy; NSAT = non‐subcutaneous adipose tissue; NVAT = non‐visceral adipose tissue; OR = odds ratio; PDFF = proton density fat fraction; PR = progesterone receptor; PSAT = deep posterior subcutaneous adipose tissue; ROI = region of interest; SAT = subcutaneous adipose tissue; SEE = standard error of estimate; SFA = subcutaneous fat area; SSAT = superficial subcutaneous adipose tissue; TAT = total adipose tissue; TFA = total fat area; UWW = underwater weighing; VAT = visceral adipose tissue; VFA = visceral fat area.

Our pooled meta‐analysis reveals transformative efficiency in AI‐driven adiposity imaging models but highlights significant trade‐offs in consistency. Deep learning architectures such as 3D‐Unet achieved a mean DSC of 0.97 (95% CI: 0.96–0.98) with a Hausdorff Distance (HD) of 1.58 mm, and CDF‐Net delivered a DSC of 0.95 (95% CI: 0.93–0.97) alongside an *R* ^2^ of 0.99. The HD quantifies boundary agreement between segmented regions, with lower values indicating more precise contour alignment, whereas *R* ^2^ reflects the proportion of variance explained relative to reference measurements. These methods reduced processing times by over 99% (0.05–3.5 min per patient vs. 10–46.5 min for conventional approaches; *p* < 0.01) (Table 3). However, automated pipelines exhibited greater variability: Mean bias was −26.2 (SD: 606) compared with −18.8 (SD: 158) for manual/semiautomated methods. More strikingly, the 95% limits of agreement for AI were nearly four times wider, spanning −1213 to 1161 versus −328 to 290 for conventional techniques, indicating substantially greater measurement variability (Figure S2).

**TABLE 3:** Performance metrics comparison between automated and conventional adipose tissue segmentation techniques.

Technique	Model	Accuracy	Sensitivity	Specificity	Precision	HD (mm)^a^	*R* ^2^	DSC	Observer variability^b^	Processing speed (minutes per patient)
Automated	2S‐CNN	0.96	0.94	0.96	NR	NR	0.99	0.95	NR	NR
Automated	3D‐Unet	0.99	0.98	0.96	0.97	1.58	0.97	0.97	NR	0.25
Automated	AF‐GWO NN	0.99	0.981	0.98	0.97	NR	0.98	0.98	NR	3.5
Automated	QFAT 2.0	NR	0.971	NR	0.97	0.99	NR	NR	0.01	2.5
Automated	CDF‐Net	0.96	NR	NR	0.98	0.99	0.92	NR	0.020	0.05
Automated	B3PDA	NR	NR	NR	NR	NR	NR	0.98	NR	NR
Automated	CCRG	NR	NR	NR	NR	NR	0.80	NR	0.020	5.0
Automated	CSA	NR	NR	NR	NR	NR	0.77	NR	NR	NR
Automated	FCM	NR	NR	NR	NR	NR	NR	0.97	NR	NR
Automated	RGA	NR	NR	NR	NR	1.54	NR	NR	NR	NR
Conventional	KMC	NR	0.996	0.953	NR	26.5	0.962	0.82	0.124	NR
Conventional	MATLAB‐VP	0.99	0.993	0.98	0.98	NR	0.76	NR	NR	0.54
Conventional	PA (Hologic)	0.99	0.993	0.98	0.98	NR	0.91	NR	NR	NR
Conventional	SOM 4.3	0.96	0.96	0.96	0.98	NR	0.846	NR	0.0123	46.5
Conventional	AMRA‐P	NR	NR	NR	NR	NR	0.93	NR	0.0037	10.0
Conventional	TBS	NR	NR	NR	NR	NR	0.87	NR	0.039	22.0

*Note:* Accuracy = proportion of correctly classified pixels/voxels; sensitivity = true positive rate; specificity = true negative rate; precision = positive predictive value.

Abbreviations: 2S‐CNN = Two‐Step Convolutional Neural Network; 3D‐Unet = 3D U‐Net Architecture; AF‐GWO NN = Adaptive Fruitfly–Grey Wolf Optimizer Neural Network; AMRA‐P = AMRA Profiler; B3PDA = Berglund's 3‐Point Dixon Algorithm; CCRG = Confidence‐Connected Region Growing; CDF‐Net = Contextual Dynamic Fusion Network; CSA = CoreScan Algorithm; DSC = Dice similarity coefficient; FCM = Fuzzy C‐Means; HD = Hausdorff distance; KMC = K‐Means Clustering; MATLAB‐VP = MATLAB‐based Voxel Peeling; NR = not reported; PA = Proprietary Algorithm (Hologic); QFAT 2.0 = Quantitative Fat Analysis Tool 2.0; *R* ^2^ = coefficient of determination; RGA = Region‐Growing Algorithm; RMSE = root mean square error; SD = standard deviation; SOM 4.3 = Slice‐O‐Matic 4.3; TBS = Threshold‐Based Segmentation.

^a^ HD values are expressed in millimeters (mm); lower values indicate better boundary alignment accuracy. DSC ranges from 0 to 1, with higher values indicating better segmentation overlap.

^b^ Observer variability was reported as RMSE or SD between observers; lower values indicate higher reproducibility.

According to the sample sized weighted meta‐analytics, a methodological discordance persisted across volumetric outcomes. Subcutaneous fat volumes ranged from 3648 to 57,211 cm^3^, producing a prediction interval from −1605 to 46,677 cm^3^ (τ = 13,862), and visceral fat volumes (VFVs) extended from −1783 to 6869 cm^3^, bounds that are biologically implausible. These wide and partially nonphysiological intervals reflect underlying methodological heterogeneity rather than true biological variability. Protocol fragmentation was stark: CT attenuation thresholds spanned 244 HU (Hounsfield Units) (−274 to −30 HU), representing a 153% Protocol Fragmentation Index relative to standard ranges. This index reflects the degree of deviation from established reference thresholds, with higher values indicating greater methodological inconsistency across studies. Across modalities, DXA‐derived subcutaneous and VFVs were 5.6‐ and 3.85‐fold higher, respectively, than MRI‐derived values, indicating that modality choice alone introduces up to 5.6× variability (Table 4).

**TABLE 4:** Modality and segmentation stratified fat metric estimates (weighted by sample size; women with obesity or overweight).

Fat metric	Grouping		Estimates		Heterogeneity/*I* ^2^ (%)	95% confidence interval	95% prediction interval
	Modality	Segmentation	*N*	Mean (SD)			
Subcutaneous fat volume (cm^3^)	All modalities		6497	7205 (4207)	100	6766–7644	−28,194–42,604
	CT	Conventional	101	12,856 (1524)	100	11,225–14,486	−3531–29,242
	DXA	Automated	69	57,211 (20,985)	0	—	—
	DXA	Conventional	258	33,529 (11,708)	100	30,884–36,175	−8962–76,021
	MRI	Conventional	6138	6006 (3602)	100	5587–6424	−26,780–38,791
Subcutaneous fat rea (cm^2^)	All modalities		5568	184 (108)	100	180–189	−156–524
	CT	Conventional	5146	176 (110)	99.99	172–181	−121–474
	DXA	Conventional	258	379 (98)	99.99	365–393	151–606
	MRI	Conventional	164	118 (82)	99.99	66–171	−557–794
Visceral fat volume (cm^3^)	All modalities		6548	2926 (1958)	100	2811–3041	−6412–12,264
	CT	Conventional	178	2457 (316)	100	2032–2883	−3222–8137
	DXA	Automated	69	22,473 (11,124)	0	—	—
	DXA	Conventional	189	8627 (3273)	100	8039–9216	532–16,723
	MRI	Conventional	6112	2543 (1547)	100	2487–2598	−1783–6869
Visceral fat area (cm^2^)	All modalities		5762	106 (78)	100	104–108	−63–275
	CT	Conventional	5146	108 (81)	99.9	106–110	−57–274
	DXA	Conventional	258	135 (52)	99.9	127–144	19–251
	MRI	Conventional	358	56 (43)	99.9	48–63	−81–192
Total fat volume (cm^3^)			6759	11,918 (8262)	100	11,308–12,528	−38,233–62,068
Total fat area (cm^2^)			5439	296 (189)	100	290–302	−174–766

*Note:* Subcutaneous fat volume and visceral fat volume are reported in cubic centimeters (cm^3^); subcutaneous fat area, visceral fat area, and total fat area are reported in square centimeters (cm^2^). Prediction intervals reflect expected variability of estimates across future comparable studies and indicate uncertainty in generalizability. *I* ^2^ values approaching 100% indicate extreme between‐study heterogeneity. – indicates data not reported or insufficient for subgroup meta‐analysis.

Abbreviations: CI = confidence interval; CT = computed tomography; DXA = dual‐energy X‐ray absorptiometry; *I* ^2^ = Higgins inconsistency statistic for heterogeneity; MRI = magnetic resonance imaging; SD = standard deviation.

**FIGURE 2:**
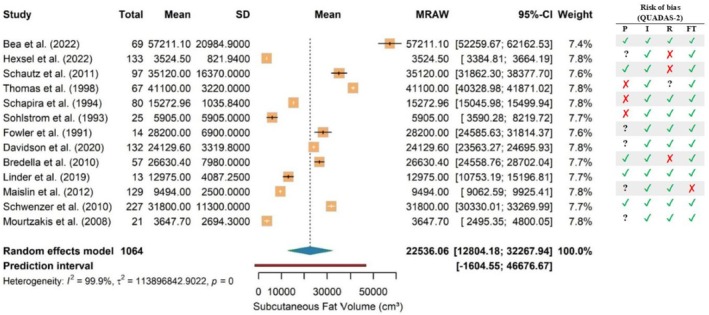
Meta‐analysis of subcutaneous fat volume (SFV) across 13 studies (*n* = 1064) revealed extreme heterogeneity (*I* ^2^ = 99.9%), with values ranging from 3647 to 57,211 cm^3^ and a pooled estimate of 22,536 cm^3^ (95% CI: 12,804–32,268). QUADAS‐2 risk‐of‐bias assessment demonstrated low risk in 38.5% of studies for patient selection, 92.3% for index test, 69.2% for reference standard, and 92.3% for flow/timing. Unclear risk was observed in 38.5% of studies for patient selection and 7.7% for index test, whereas high risk was identified in 23.1% of studies for patient selection, 30.8% for reference standard, and 7.7% for flow/timing.

An inverse‐variance random‐effects model confirmed that neither planar nor volumetric adiposity measures escape methodological confounding. For subcutaneous fat volume, the pooled mean estimate of 22,536 cm^3^ (95% CI: 12,804–32,268) was overshadowed by a prediction interval of −1605 to 46,677 cm^3^ and near‐total heterogeneity (*I* ^2^ = 99.9%, τ = 10,672 cm^3^) (Figure 2). These findings underscore the necessity of harmonized acquisition and analysis protocols to translate AI's technical promise into clinically reliable adiposity metrics.

**FIGURE 3:**
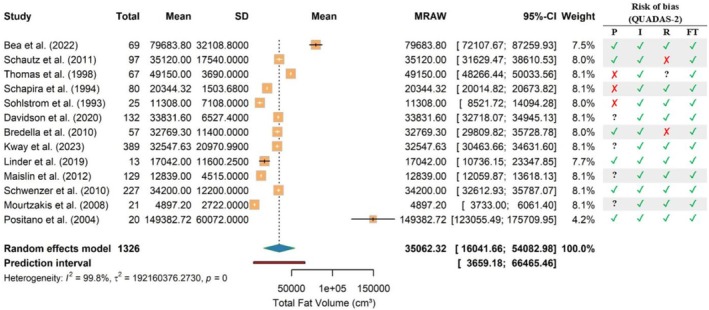
Forest plot displays meta‐analysis of total fat volume (TFV) across 13 studies (*n* = 1326), demonstrating extreme heterogeneity (*I* ^2^ = 99.8%) with values ranging from 4897 to 149,383 cm^3^. The pooled estimate was 35,062 cm^3^ (95% CI: 16,042–54,083) with a prediction interval of 3659–66,465 cm^3^, highlighting substantial between‐study variability in TFV quantification. QUADAS‐2 risk‐of‐bias assessment showed low risk in 61.5% of studies for patient selection, 100% for index test, 76.9% for reference standard, and 100% for flow/timing. High risk was identified in 23.1% of studies for patient selection and 15.4% for reference standard.

The meta‐analysis of VFV yielded a pooled mean estimate of 3615 cm^3^ (95% CI: 1487–5743), but this result spanned a 1500‐fold range and exhibited residual heterogeneity (*I* ^2^ = 100%, τ = 391 cm^3^), effectively precluding clinical interpretation. Total fat volume (TFV) performed only marginally better, with a pooled estimate of 35,062 cm^3^ (95% CI: 16,042–54,083) and a prediction interval from 3659 to 66,465 cm^3^; heterogeneity remained extreme (*I* ^2^ = 99.8%, τ = 13,862 cm^3^) (Figure 3).

**FIGURE 4:**
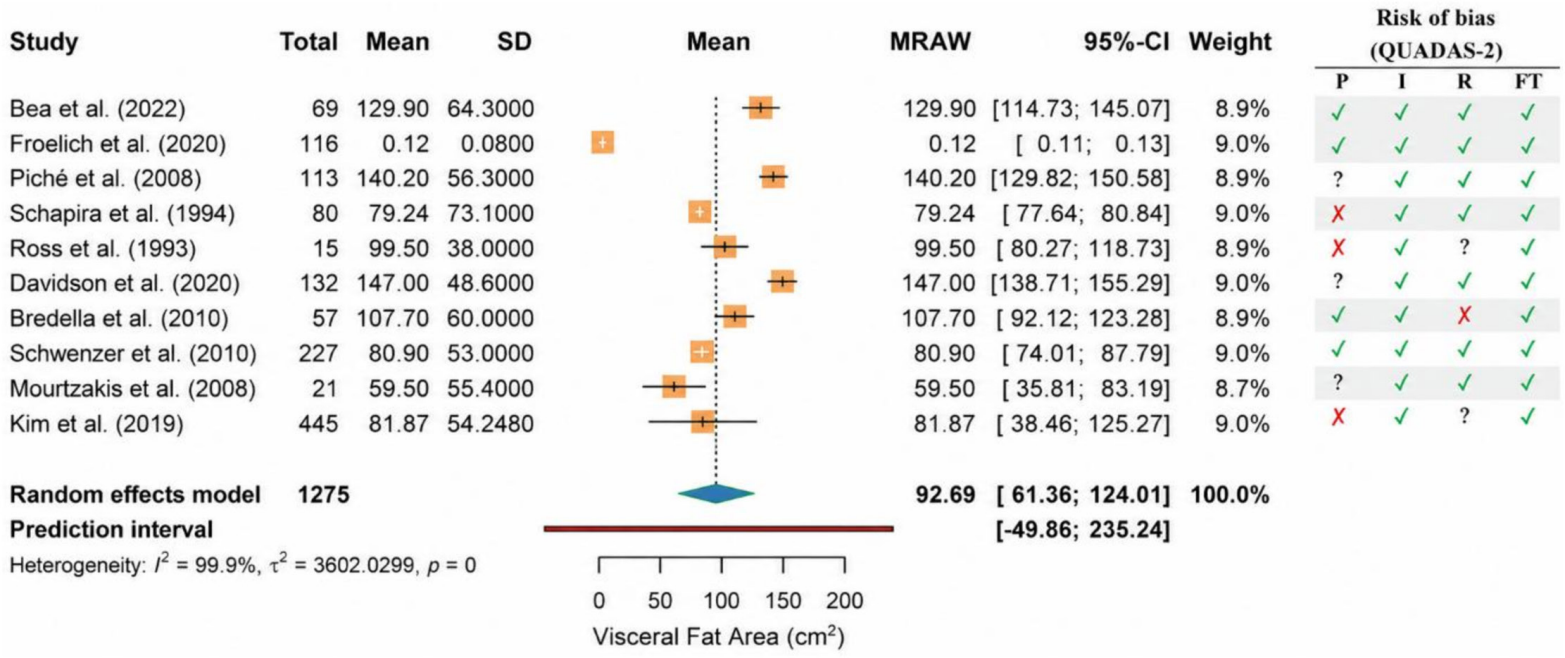
Meta‐analysis of visceral fat area (VFA) across 10 studies (*n* = 1275) revealed extreme heterogeneity (*I* ^2^ = 99.9%), with values ranging from 0.12 to 147.00 cm^2^ and a pooled estimate of 92.69 cm^2^ (95% CI: 61.36–124.01). Risk‐of‐bias assessment using QUADAS‐2 showed low risk in 40% of studies for patient selection, 100% for index test, 70% for reference standard, and 100% for flow/timing. Unclear risk was observed in 30% of studies for patient selection and 20% for reference standard, whereas high risk was identified in 30% of studies for patient selection and 10% for reference standard.

Planar measures showed the same pattern: Subcutaneous fat area (SFA) pooled to 312.22 cm^2^ (95% CI: 194.72–429.73) but encompassed anatomically impossible values (prediction interval −89.65 to 714.10 cm^2^), whereas visceral fat area (VFA) averaged 92.69 cm^2^ (95% CI: 61.36–124.01) and total fat area (TFA), 411.12 cm^2^ (95% CI: 289.90–532.35), each with *I* ^2^ ≥ 98.9% and invalid prediction bounds (Figure 4).

**FIGURE 5:**
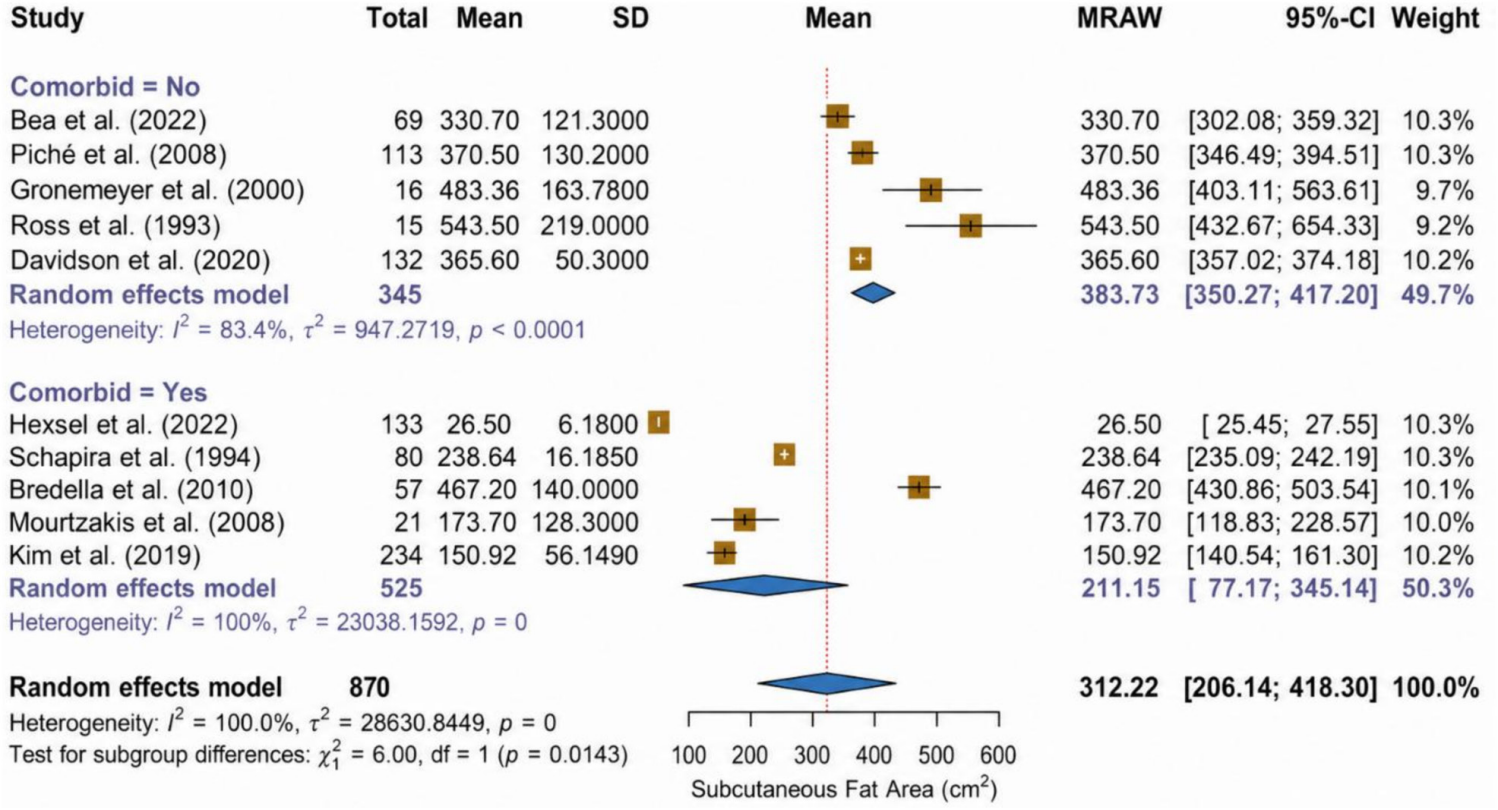
Forest plot comparing subcutaneous fat area in patients without (*n* = 345) versus with comorbidities (*n* = 525). Significant subgroup differences were observed (*p* = 0.014), with non‐comorbid patients showing higher fat areas (383.73 cm^2^, 95% CI: 350.27–417.20; *I* ^2^ = 83.4%) versus comorbid patients (211.15 cm^2^, 95% CI: 77.17–345.14; *I* ^2^ = 100%). Extreme overall heterogeneity persisted (*I* ^2^ = 100%), suggesting comorbidities may partially explain measurement variability.

Subgroup analyses offered only partial insight into variability sources and failed to resolve the pervasive heterogeneity. For SFV, women weighing > 75 kg had a higher pooled volume (36,042 cm^3^) than those ≤ 74.9 kg (19,959 cm^3^; *p* < 0.01), and DXA‐derived volumes exceeded MRI‐ and CT‐derived volumes (*p* = 0.02), yet *I* ^2^ remained ≥ 99.8%. SFA differed by comorbidity status (383.73 cm^2^ without comorbidity vs. 320.84 cm^2^ with comorbidity; *p* = 0.014) and modality (*p* = 0.015), but *I* ^2^ never fell below 83.4% (Figure 5; Figure S24).

**FIGURE 6:**
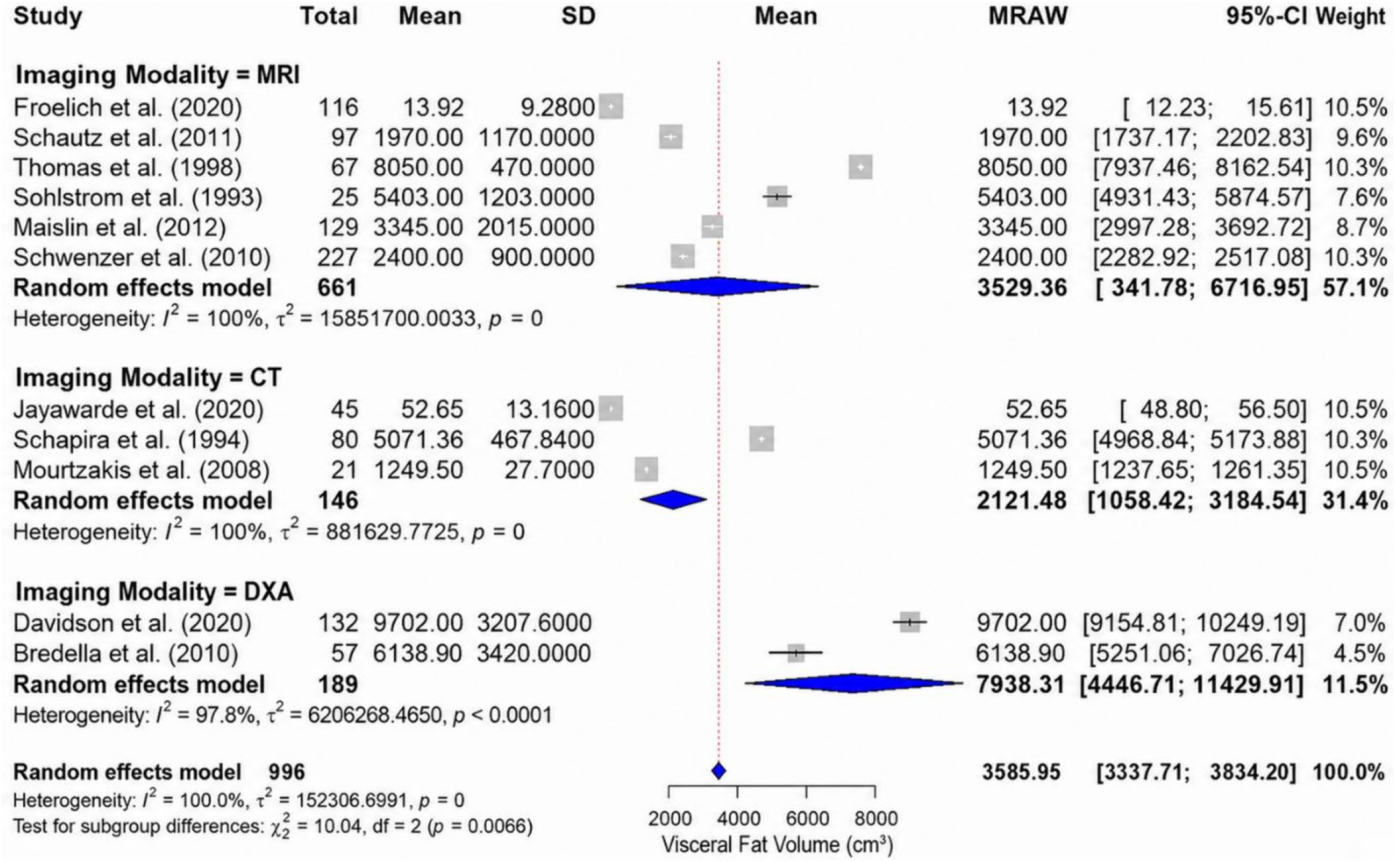
Forest plot of visceral fat volume (VFV) across 11 studies (*n* = 996) stratified by imaging modality (MRI, CT, and DXA). Extreme heterogeneity was observed overall (*I* ^2^ = 100%) and within subgroups (MRI: *I* ^2^ = 100%; CT: *I* ^2^ = 100%; DXA: *I* ^2^ = 97.8%). The test for subgroup differences was significant (*p* = 0.007), indicating imaging modality contributes to variability.

The meta‐analysis revealed significant differences in VFV measurements across imaging modalities (DXA > MRI > CT; *p* = 0.007), with extreme heterogeneity (*I* ^2^ = 100%) indicating substantial variability between studies. MRI studies showed the widest range of mean VFV values (13.92–8050 cm^3^), whereas CT results varied moderately (52.65–5071.36 cm^3^), and DXA yielded consistently higher measurements (6138.90–9702.00 cm^3^) (Figure 6). Notably, VFV was elevated in women with waist circumference greater than 95 cm (3345 cm^3^ vs. 2189 cm^3^; *p* < 0.0001), maintaining the same modality hierarchy (*p* = 0.007). VFA also varied by obesity status (117.21 cm^2^ in women living with obesity vs. 85.82 cm^2^ in overweight; *p* = 0.016) and body region (118.83‐cm^2^ regional vs. 81.38‐cm^2^ whole‐body; *p* = 0.002), though heterogeneity remained high (*I* ^2^ ≥ 54.7%). The detailed forest plots and subgroup analysis were provided in Figures S3–S41.

Sensitivity analyses confirmed statistical stability but underscored clinical irrelevance. All sensitivity analyses maintained extreme heterogeneity (*I* ^2^ ≥ 98%), despite minor shifts in point estimates (SFV: 19,777–24,128 cm^3^; TFV: 30,073–37,626 cm^3^). Trim‐and‐fill adjustments collapsed SFA to 69.92 cm^2^ and VFA to 14.02 cm^2^, statistically significant yet biologically implausible given unaddressed protocol variability. Excluding outliers such as an anomalously low VFA (0.12 cm^2^) improved point estimates (VFA: 104.01 cm^2^) but left *I* ^2^ ≥ 98.1%. Similarly, trimming TFV outliers [52, 73] reduced the estimate to 25,852 cm^3^ (95% CI: 16,865–34,840) without resolving discordance (Figures S42–S49; Table S5).

These pooled and subgroup analyses demonstrate that methodological artifacts across acquisition, segmentation, and definition overwhelm any true biological signal in AI‐driven adiposity quantification. Without urgent standardization, even the most robust meta‐analytic methods yield results that are statistically consistent yet clinically meaningless.

## 4 Discussion

This systematic review and meta‐analysis of 38 studies (*n* = 12,815) reveal a distinct paradox in adiposity imaging. While AI‐driven methods demonstrate near‐perfect segmentation accuracy and more than 99% reductions in processing time, pooled volumetric estimates are rendered biologically implausible by extreme protocol variability. Across all adiposity metrics, heterogeneity was extreme (*I* ^2^ ≈ 100%), producing prediction intervals that span nonphysiological values (e.g., a TFV prediction interval of −38,233 to 62,068 cm^3^). These outcomes reflect methodological fragmentation and statistical artifacts arising from extreme between‐study heterogeneity rather than true biological diversity.

Our findings echo reproducibility challenges reported in oncology radiomics, where inconsistent scanning parameters and feature‐extraction workflows have invalidated cross‐study comparisons [88]. The IBSI has advocated consensus definitions and harmonization of radiomic features, yet fewer than 10% of adipose‐related biomarkers achieve robust reproducibility without standardized protocols. Moreover, unlike mixed‐sex meta‐analyses that attribute heterogeneity partly to biological factors such as sex hormones, our sex‐specific synthesis indicates that technical factors overwhelmingly drive volumetric discrepancies.

Our results confirm AI's technical prowess—models like 3D‐UNet reduced processing times by > 99% and achieved near‐perfect segmentation accuracy (DSC = 0.97) [27, 65]. Yet, these technical strengths fail to translate into reliable population‐level insights due to variations in acquisition parameters and analytic pipelines. For example, CT attenuation thresholds spanned 244 HU (−274 to −30 HU) [66] exceeding accepted reference ranges by 153%. Furthermore, DXA‐derived VFVs were nearly five times higher than MRI‐derived estimates (12,330 [27] vs. 2543 [26] cm^3^; *p* < 0.001), a discrepancy with direct clinical implications. A woman may be classified as “high‐risk” by one modality and “low risk” by another, introducing potential misclassification in metabolic risk assessment.

Moreover, protocol‐driven variability disproportionately affects underrepresented groups. South Asian women, who have 12%–15% higher visceral adiposity at equivalent BMIs, are still assessed using Eurocentric diagnostic thresholds [89, 90, 91]. Geographic and demographic biases exacerbate this inequity: 72% of included cohorts came from high‐income countries, whereas only 2.6% were African and 15.4% Asian. Participant ethnicity or race was inconsistently reported across studies; however, where available, cohorts were predominantly of European or White background. This imbalance limits the global generalizability of pooled estimates and may introduce bias, particularly given known ethnic differences in fat distribution and cardiometabolic risk profiles. This mirrors disparities reported in dermatology AI, where performance dropped by 50% in Black patients due to training set homogeneity [92].

The strengths of this review include its adherence to PRISMA 2020 guidelines and its large, sex‐specific pooled sample, making it the most comprehensive meta‐analysis of AI‐driven adiposity imaging in women to date. However, limitations remain. The pooled sample was relatively older (mean age ≈ 51 years), which may limit generalizability to younger women and those at earlier stages of adiposity accumulation. There was a notable imbalance in imaging modalities, with MRI‐dominated datasets, and AI validation subgroups were often small (*n* = 16–69), which may limit the robustness and external validity of AI performance estimates in smaller cohorts. In addition, publication bias cannot be excluded, as studies reporting positive or high‐performing AI segmentation results may have been preferentially published, potentially inflating pooled estimates of segmentation accuracy and reproducibility. Furthermore, most studies did not report radiomic texture or heterogeneity biomarkers, limiting insight into fat tissue characteristics beyond volume. This absence represents an important methodological limitation, as such features may provide more detailed characterization of adipose tissue heterogeneity and biological behavior beyond volumetric assessment.

These findings challenge the assumption that biological differences explain the wide variability in adiposity estimates. Instead, the evidence points to protocol heterogeneity as the principal confounder. Automated methods, while computationally efficient, introduced greater volumetric variability than manual techniques (e.g., limits of agreement: −1213 to 1161 cm^3^ vs. –328 to 290 cm^3^). This divergence highlights the double‐edged nature of AI: precision in isolation, but unreliability in aggregation. Our results also differ from previous mixed‐sex meta‐analyses, which often attributed variability to hormonal or anthropometric factors—underscoring the distinct challenges of conducting sex‐specific adiposity research.

To harness AI's full potential, field‐wide standardization is imperative. We strongly recommend establishing a global consensus framework for adiposity imaging protocols, coordinated by international radiology and obesity research consortia. This framework should include minimum reporting standards for AI model architecture, imaging parameters (e.g., slice thickness and body region), and segmentation thresholds tailored by sex and ethnicity. Examples include the use of L2–L3 MRI slices for VAT estimation and sex‐specific HU thresholds (e.g., −140 to −40 HU for women). Regulatory agencies should mandate transparent external validation and demographic breakdowns in AI performance metrics to mitigate bias and ensure reproducibility. To operationalize these standards, a federated data‐sharing infrastructure could enable decentralized model training across centers, preserving data privacy while improving generalizability. In parallel, academic journals and funding bodies should require adherence to open‐source reproducibility guidelines and endorse repositories of prevalidated, protocol‐harmonized datasets. These collective steps can accelerate cross‐institutional comparability.

Future research should prioritize creating female‐centric datasets that include underrepresented populations. Furthermore, integrating imaging data with hormonal and metabolic biomarkers (e.g., leptin and adiponectin) is essential to reflect the biological complexity of adipose tissue. Longitudinal studies capturing fat distribution changes over the life course will be critical in improving both prevention and treatment strategies.

## 5 Conclusion

AI‐powered segmentation offers unprecedented efficiency and technical precision in adiposity imaging among women with overweight and obesity. However, without protocol harmonization, its outputs lack clinical interpretability. Our findings reveal that methodological fragmentation—more than biological variability—drives the inconsistencies in reported fat volumes. To realize AI's full potential in obesity care, the field must urgently prioritize standardization, inclusivity, and translational relevance. Broader clinical adoption of AI‐based adiposity imaging should be considered only after imaging protocols are standardized, algorithms are validated across diverse populations, and technical platforms are harmonized to ensure reliability and generalizability.

## Author Contributions

A.A.T. conceived the idea for this review, designed the review protocol, and developed the search strategy. A.A.T. conducted the literature searches across all databases. A.A.T. and B.T.T. independently screened references, determined eligibility, extracted data, assessed risk of bias, and conducted meta‐analyses. A.A.T. oversaw and adjudicated the study selection process. A.A.T. and B.T.T. jointly drafted the manuscript, contributed to revisions, and critically reviewed the final content. A.A.T. and B.T.T. directly accessed and verified the underlying data. Both authors independently validated study eligibility, data extraction, and analytical processes to ensure accuracy. All authors had full access to all data in the study and assume responsibility for its integrity, analysis, and interpretation. A.A.T. and B.T.T. jointly hold final responsibility for the decision to submit for publication.

## Funding

The authors have nothing to report.

## Conflicts of Interest

The authors declare no conflicts of interest.

## Supporting information


**Appendix S1:** Comprehensive search strategies for radiomics and obesity research across multiple databases.
**Appendix S2:** QUADAS‐2 assessment of risk of bias and applicability concerns across 38 studies. High risk of bias was most frequent in‐patient selection (21%, 8/38), while index test showed minimal bias (0% high risk). Applicability concerns were prominent in‐patient selection (63%, 24/38 high concern) but negligible for index/reference standards (0% high concern). Unclear ratings were common in reference standard (34%, 13/38) and flow/timing (8%, 3/38), reflecting reporting limitations.
**Appendix S3:** Multimodality imaging protocols and technical parameters for adiposity quantification across studies (1990–2025).
**Appendix S4:** Bland–Altman plot comparing automated versus conventional methods for fat volume measurement shows a mean bias of −22.3 cm^3^ (95% limits of agreement: −854.94 to 810.34), indicating no systematic measurement bias between methods despite wide variability in individual measurements (range: 0–30,000 cm^3^).
**Appendix S5:** Forest plot presents meta‐analysis of total fat area (TFA) measurements, showing high heterogeneity across studies (*I* ^2^ = 98.9%, τ^2^ = 16900.48) with individual study means ranging from 232.79 to 643.00 cm^2^ and pooled estimate of 411.12 cm^3^ (95% CI: 289.90–532.35). The wide prediction interval (94.16–728.09 cm^2^) reflects substantial between‐study variability.
**Appendix S6:** Forest plot displays subgroup analysis of total fat volume (TFV) by age categories (median age > 47.1 vs. ≤ 47.1 years), revealing no significant differences between age groups (*p* = 0.854) despite extreme heterogeneity in both subgroups (*I* ^2^ = 99.8%). Younger participants showed wider TFV range (11,308–49,150 cm^3^) compared to older participants (4897–79,684 cm^3^).
**Appendix S7:** Forest plot presents subgroup analysis of total fat volume (TFV) by body region (regional vs. whole‐body), showing no significant differences between measurement approaches (*p* = 0.939) despite extreme heterogeneity in both subgroups (*I* ^2^ = 99.6%–99.9%). Regional measurements demonstrated wider TFV range (4897–149,383 cm^3^) compared to whole‐body assessments (20,344–49,150 cm^3^).
**Appendix S8:** Forest plot displays subgroup analysis of TFV by comorbidity status, revealing no significant differences between groups with and without comorbidities (*p* = 0.756), though comorbidity‐positive studies showed greater heterogeneity (*I* ^2^ = 99.9% vs. 98.5%). The comorbidity group included both the lowest (4897 cm^3^) and highest (149,383 cm^3^) observed TFV values.
**Appendix S9:** Forest plot illustrates subgroup analysis of TFV by imaging modality (DXA/MRI/CT), demonstrating significant differences between modalities (*p* = 0.003) with MRI studies showing the widest TFV range (11,308–149,383 cm^3^) and highest heterogeneity (*I* ^2^ = 99.8%, τ^2^ = 398,752,853). DXA measurements were consistently higher (32,769–79,684 cm^3^) than CT values (4897–20,344 cm^3^).
**Appendix S10:** Forest plot of total fat volume (TFV) across 13 studies (*n* = 1326) stratified by obesity status (obese, overweight). Extreme heterogeneity was noted overall (*I* ^2^ = 99.8%) and within subgroups (Obese: *I* ^2^ = 99.9%; Overweight: *I* ^2^ = 99.1%). TFV ranged from 4897 cm^3^ (Mourtzakis et al. 2008) to 149,383 cm^3^ (Positano et al. 2004). The pooled estimate was 35,062 cm^3^ (95% CI: 27,328–42,797), with a prediction interval of 16,042–54,083 cm^3^. Subgroup differences were significant (*p* = 0.048).
**Appendix S11:** Forest plot of total fat area (TFA) across nine studies (*n* = 513). Extreme heterogeneity was observed (*I* ^2^ = 98.9%), with means ranging from 232.79 cm^2^ (Ki et al. 2019) to 643.00 cm^2^ (Ross et al. 1993). The pooled estimate was 411.12 cm^2^ (95% CI: 289.90–532.35), and the prediction interval (94.16–728.09 cm^2^) underscored high between‐study variability.
**Appendix S12:** Forest plot of total fat area across nine studies (*n* = 493) stratified by age (median age ≤ 47.1 vs. > 47.1 years). Extreme heterogeneity was observed overall (*I* ^2^ = 99.0%) and within subgroups (> 47.1: *I* ^2^ = 95.2%; ≤ 47.1: *I* ^2^ = 98.3%). The pooled estimate was 434.25 cm^2^ (95% CI: 338.52–529.97), with older participants showing lower but nonsignificant differences (subgroup *p* = 0.374).
**Appendix S13:** Forest plot of TFA across nine studies (*n* = 513) comparing regional vs. whole‐body measurements. Extreme heterogeneity persisted overall (*I* ^2^ = 98.9%) and within subgroups (Regional: *I* ^2^ = 97.5%; Whole‐body: *I* ^2^ = 95.8%). The pooled estimate was 411.12 cm^2^ (95% CI: 323.64–498.61), with no significant subgroup differences (*p* = 0.655). Notably, whole‐body studies showed wider variability (e.g., 317.88 to 643.00 cm^2^ vs. 232.79–574.90 cm^2^ for regional).
**Appendix S14:** Forest plot of TFA across nine studies (*n* = 513) stratified by comorbidity status. Significant subgroup differences were detected (*p* = 0.0006), with non‐comorbid participants showing higher pooled estimates (506.62 cm^2^, 95% CI: 472.73–540.51) and lower heterogeneity (*I* ^2^ = 66.5%) compared to comorbid groups (320.84 cm^2^, 95% CI: 220.68–421.01; *I* ^2^ = 96.6%). Extreme overall heterogeneity was observed (*I* ^2^ = 98.9%).
**Appendix S15:** Forest plot of TFA across nine studies (*n* = 513) stratified by imaging modality. Significant subgroup differences were observed (*p* = 0.021), with DXA showing the most consistent measurements (*I* ^2^ = 81.7%, pooled estimate = 514.07 cm^2^ [95% CI: 464.40–563.74]). Extreme heterogeneity was noted in CT (*I* ^2^ = 97.6%) and MRI (*I* ^2^ = 97.1%) subgroups, with MRI demonstrating the widest variability (233.41–643.00 cm^2^). The overall pooled estimate was 411.12 cm^2^ (95% CI: 323.64–498.61) with extreme heterogeneity (*I* ^2^ = 98.9%).
**Appendix S16:** Forest plot of TFA across nine studies (*n* = 513) comparing obese (*n* = 376) and overweight (*n* = 137) populations. No significant subgroup differences were found (*p* = 0.752), despite extreme heterogeneity in both groups (obese: *I* ^2^ = 97.3%; overweight: *I* ^2^ = 98.9%). The obese subgroup showed a pooled estimate of 401.27 cm^2^ (95% CI: 302.45–500.09), while overweight studies demonstrated wider variability (317.88–574.90 cm^2^). The overall estimate was 411.12 cm^2^ (95% CI: 323.64–498.61).
**Appendix S17:** Forest plot of TFA across seven studies (*n* = 415) stratified by waist circumference (median in meter ≤ 0.95 vs. > 0.95). No subgroup differences were detected (*p* = 0.944), though both groups showed extreme heterogeneity (≤ 0.95: *I* ^2^ = 99.5%; > 0.95: *I* ^2^ = 96.2%). The ≤ 0.95 subgroup had a pooled estimate of 450.05 cm^2^ (95% CI: 320.02–580.09), while the > 0.95 subgroup showed paradoxical results (232.79–643.00 cm^2^). The overall estimate was 442.87 cm^2^ (95% CI: 333.90–551.85).
**Appendix S18:** Forest plot comparing subcutaneous fat volume stratified by body weight (> 75 kg, *n* = 391) vs. below the median value (≤ 74.9, *n* = 308) populations across eight studies (*n* = 699). Significant subgroup differences were found (*p* = 0.0005), with high weight participants showing higher pooled volumes (34,155.82 cm^3^, 95% CI: 27,772.25–40,539.39) compared to participants having lower weight (24,129.60–28,200.00 cm^3^). Extreme heterogeneity was observed in both subgroups (> 75 kg: *I* ^2^ = 98.4%; ≤ 74.9: *I* ^2^ = 99.6%) and overall (*I* ^2^ = 99.9%).
**Appendix S19:** Forest plot of seven studies (*n* = 594) stratified by height above and below the median value in meter (> 1.60 vs. ≤ 1.60). Significant subgroup differences emerged (*p* = 0.0099), with higher height groups demonstrating greater fat volumes (34,155.82 cm^3^, 95% CI: 27,772.25–40,539.39) versus lower‐height groups (25,173.32 cm^3^, 95% CI: 22,756.29–27,590.35). Notably, heterogeneity was substantially lower in the ≤ 1.60 subgroup (*I* ^2^ = 80.8% vs. 98.1%).
**Appendix S20:** Forest plot of 12 studies (*n* = 995) significant differences in subcutaneous fat volume (SFV) comparing MRI (*n* = 705), CT (*n* = 101), and DXA (*n* = 189) measurements. Significant modality effects were found (*p* = 0.0241), with MRI showing the widest range (3524.50–41,100.00 cm^3^) and highest heterogeneity (*I* ^2^ = 99.9%). DXA measurements demonstrated the lowest heterogeneity (*I* ^2^ = 80.8%). The overall estimate was 31,168.37 cm^3^ (95% CI: 23,351.11–38,985.64) with extreme heterogeneity (*I* ^2^ = 99.9%).
**Appendix S21:** Forest plot presents subgroup analysis of subcutaneous fat volume (SFV) by age categories (≤ 47.1 vs. > 47.1 years), showing no significant difference between age groups (*p* = 0.090) despite extreme heterogeneity in both subgroups (*I* ^2^ = 100% and 99.8%, respectively), with younger participants demonstrating wider SFV range (3524–41,100 cm^3^) compared to older participants (3647–24,130 cm^3^).
**Appendix S22:** Forest plot displays subgroup analysis of SFV by body mass percentage (BMP) (≤ 35% vs. > 35%) revealing no significant subgroup differences (*p* = 0.780) with persistent extreme heterogeneity (*I* ^2^ = 100%) across all studies, though higher BMP group showed greater variability in SFV measurements (5905–35,120 cm^3^ vs. 3525–41,100 cm^3^).
**Appendix S23:** Forest plot illustrates subgroup analysis of SFV by body region (regional vs. whole‐body), demonstrating no significant measurement differences (*p* = 0.121) despite substantial heterogeneity in both subgroups (*I* ^2^ = 99.9%), with whole‐body assessments showing higher mean SFV (29,093 cm^3^) compared to regional measurements (15,111 cm^3^).
**Appendix S24:** Forest plot presents subgroup analysis of subcutaneous fat volume (SFV) by comorbidity status, showing no significant difference between groups with and without comorbidities (*p* = 0.236), with similarly extreme heterogeneity in both subgroups (*I* ^2^ = 100% vs. 99%) and comparable SFV ranges (3647–41,100 cm^3^ with comorbidities vs. 5905–35,120 cm^3^ without).
**Appendix S25:** Forest plot displays subgroup analysis of SFV by obesity status (overweight vs. obese), revealing no significant differences between groups (*p* = 0.968) despite extreme heterogeneity in both subgroups (*I* ^2^ = 99.9%), with obese individuals showing wider SFV range (3647–41,100 cm^3^) compared to overweight (3525–35,120 cm^3^).
**Appendix S26:** Forest plot of subcutaneous fat area across 10 studies (*n* = 642) demonstrating extreme heterogeneity (*I* ^2^ = 100%). Individual study means ranged from 26.50 cm^2^ (Hexsel et al. 2022) to 543.50 cm^2^ (Ross et al. 1993), with a pooled estimate of 312.22 cm^2^ (95% CI: 194.72–429.73). The wide prediction interval (−89.65 to 714.10 cm^2^) highlights substantial between‐study variability in measurement techniques or populations.
**Appendix S27:** Forest plot comparing subcutaneous fat area in patients without (*n* = 345) versus with comorbidities (*n* = 297). Significant subgroup differences were observed (*p* = 0.014), with non‐comorbid patients showing higher fat areas (383.73 cm^2^, 95% CI: 350.27–417.20; *I* ^2^ = 83.4%) versus comorbid patients (211.15 cm^2^, 95% CI: 77.17–345.14; *I* ^2^ = 100%). Extreme overall heterogeneity persisted (*I* ^2^ = 100%), suggesting comorbidities may partially explain measurement variability.
**Appendix S28:** Forest plot of eight studies (*n* = 615) stratified by body fat percentage (> 35% vs. ≤ 35%). Significant differences emerged (*p* = 0.011), with higher adiposity groups demonstrating greater subcutaneous fat areas (pooled mean range: 330.70–543.50 cm^2^, *I* ^2^ = 90.5%) versus lower adiposity groups (26.50–238.64 cm^2^, *I* ^2^ = 100%). The extreme overall heterogeneity (*I* ^2^ = 100%) underscores the influence of body composition on measurement outcomes.
**Appendix S29:** Forest plot of subcutaneous fat area across 10 studies (*n* = 642) comparing regional (*n* = 531) versus whole‐body (*n* = 111) measurements. While no significant subgroup differences were found (*p* = 0.297), regional measurements showed greater variability (*I* ^2^ = 99.9%, range: 26.50–467.20 cm^2^) compared to whole‐body assessments (*I* ^2^ = 96.9%, range: 238.64–543.50 cm^2^). The pooled estimate was 312.22 cm^2^ (95% CI: 206.14–418.30) with extreme overall heterogeneity (*I* ^2^ = 100%), suggesting measurement methodology may influence results despite nonsignificant subgroup effects.
**Appendix S30:** Forest plot comparing obese (*n* = 372) and overweight (*n* = 270) populations across 10 studies. No significant differences were observed (*p* = 0.325), though obese individuals showed slightly higher pooled fat areas (334.64 cm^2^, 95% CI: 275.57–393.70) with lower heterogeneity (*I* ^2^ = 96.1%) compared to overweight groups (243.02 cm^2^, 95% CI: 70.22–415.82; *I* ^2^ = 100%). The extreme overall heterogeneity (*I* ^2^ = 100%) indicates additional unmeasured factors contribute to variability beyond obesity status.
**Appendix S31:** Forest plot evaluating automated (*n* = 85) versus conventional (*n* = 557) segmentation techniques across 10 studies. Nonsignificant differences were found (*p* = 0.244), with automated methods showing marginally higher estimates (402.24 cm^2^, 95% CI: 252.93–551.55; *I* ^2^ = 91.9%) versus conventional approaches (289.22 cm^2^, 95% CI: 171.38–407.07; *I* ^2^ = 100%). The persistent extreme heterogeneity (*I* ^2^ = 100%) across all analyses highlights the need for standardized measurement protocols in adiposity research.
**Appendix S32:** Forest plot evaluating waist circumference subgroups (≤ 0.95 m [*n* = 394] vs. > 0.95 m [*n* = 21]) across six studies. Nonsignificant differences were found (*p* = 0.924), with both subgroups showing similar estimates (≤ 0.95 m: mean not reported, 95% CI: not available, *I* ^2^ = 99.6% vs. > 0.95 m: mean not reported, 95% CI: not available, *I* ^2^ = 97.6%). The extreme heterogeneity (*I* ^2^ = 99.4%) across analyses indicates substantial variability in subcutaneous fat area measurements.
**Appendix S33:** Forest plot analyzing visceral fat volume across 13 studies (*n* = 1070). Extreme heterogeneity was observed (*I* ^2^ = 100%), with individual study means ranging from 13.92 to 22,472.70 cm^3^. The random effects model yielded a pooled estimate of 3614.81 cm^3^ (95% CI: 1486.73–5742.89), with prediction interval (2721.90–4507.72 cm^3^) reflecting substantial between‐study variability.
**Appendix S34:** Forest plot evaluating age subgroups (≤ 47.1 years [*n* = 669] vs. > 47.1 years [*n* = 327]) across 11 studies. Nonsignificant differences were found (*p* = 0.646), with younger participants showing higher estimates (4145.30 cm^3^, 95% CI: 1262.51–7028.09; *I* ^2^ = 100%) versus older participants (3440.00 cm^3^, 95% CI: 2579.48–4300.52; *I* ^2^ = 100%). The extreme heterogeneity (*I* ^2^ = 100%) persists across all analyses.
**Appendix S35:** Forest plot of visceral fat volume across six studies (*n* = 599) stratified by waist circumference in meter (≤ 0.95 vs. > 0.95). Significant subgroup differences were observed (*p* < 0.0001), with the ≤ 0.95 group showing extreme variability (13.92–9702.00 cm^3^, *I* ^2^ = 100%) compared to the > 0.95 group (3345.00 cm^3^ single study). The pooled estimate was 3585.95 cm^3^ (95% CI: 3337.71–3834.20) with extreme overall heterogeneity (*I* ^2^ = 100%), suggesting WC may be an important modifier of visceral adiposity measurements.
**Appendix S36:** Forest plot comparing whole‐body (*n* = 490) versus regional (*n* = 506) measurements across 11 studies. No significant differences were found (*p* = 0.976), though whole‐body measurements showed greater variability (13.92–8050.00 cm^3^, *I* ^2^ = 100%) versus regional assessments (52.65–9702.00 cm^3^, *I* ^2^ = 100%). The similar pooled estimates (whole‐body: 3883.64 cm^3^; regional: 3823.29 cm^3^) suggest measurement methodology may not substantially influence visceral fat quantification despite extreme heterogeneity (*I* ^2^ = 100%).
**Appendix S37:** Forest plot of 11 studies (*n* = 996) stratified by comorbidity status. Nonsignificant differences emerged (*p* = 0.134), though comorbid patients showed lower pooled volumes (3140.77 cm^3^, 95% CI: 2840.66–3440.88; *I* ^2^ = 100%) versus non‐comorbid groups (4851.26 cm^3^, 95% CI: 2636.45–7066.07; *I* ^2^ = 99.6%). The extreme overall heterogeneity (*I* ^2^ = 100%) indicates additional factors beyond comorbidity status affect measurements.
**Appendix S38:** Forest plot comparing whole‐body (*n* = 490) versus regional (*n* = 506) assessment methods across 11 studies. No significant differences were found (*p* = 0.976), with similar pooled estimates (whole‐body: 3883.64 cm^3^; regional: 3823.29 cm^3^) despite extreme heterogeneity in both subgroups (*I* ^2^ = 100%). The remarkably wide range of values (13.92–9702.00 cm^3^ across all studies) highlights substantial variability in visceral fat quantification regardless of measurement approach.
**Appendix S39:** Forest plot of visceral fat volume across 12 studies (*n* = 996) comparing obese (*n* = 465) versus overweight (*n* = 531) populations. No significant subgroup differences were observed (*p* = 0.463), though obese individuals showed higher pooled volumes (4407.97 cm^3^, 95% CI: 3463.06–5352.88) compared to overweight groups (3492.95 cm^3^, 95% CI: 1237.10–5748.80). Extreme heterogeneity was present in both subgroups (*I* ^2^ = 100%) and overall (*I* ^2^ = 100%), with values ranging from 13.92 to 9702.00 cm^3^, suggesting obesity status alone does not explain measurement variability.
**Appendix S40:** Forest plot of visceral fat area across eight studies (*n* = 714) stratified by obesity status. Significant subgroup differences were observed (*p* = 0.016), with obese individuals demonstrating higher visceral adiposity (117.21 cm^2^, 95% CI: 93.57–140.84; *I* ^2^ = 93.5%) compared to overweight subjects (85.82 cm^2^, 95% CI: 75.88–95.76; *I* ^2^ = 84.4%). The overall pooled estimate was 105.95 cm^2^ (95% CI: 83.39–128.51) with extreme heterogeneity (*I* ^2^ = 98.3%), suggesting obesity status significantly influences visceral fat accumulation but does not fully explain measurement variability.
**Appendix S41:** Forest plot comparing older (> 47.1 years, *n* = 335) versus younger (≤ 47.1 years, *n* = 379) populations. Significant differences emerged (*p* = 0.016), with older individuals showing greater visceral fat accumulation (121.59 cm^2^, 95% CI: 96.31–146.87; *I* ^2^ = 93.7%) versus younger subjects (88.13 cm^2^, 95% CI: 78.38–97.88; *I* ^2^ = 82.3%). The persistent high heterogeneity (*I* ^2^ = 98.3% overall) indicates age‐related differences in visceral adiposity, though other factors likely contribute to measurement variability.
**Appendix S42:** Forest plot of eight studies (*n* = 714) comparing regional (*n* = 392) versus whole‐body (*n* = 322) assessment methods. Highly significant differences were found (*p* = 0.002), with regional measurements yielding higher estimates (118.83 cm^2^, 95% CI: 96.27–141.40; *I* ^2^ = 93.3%) versus whole‐body approaches (81.38 cm^2^, 95% CI: 75.61–87.14; *I* ^2^ = 54.7%). The substantially lower heterogeneity in whole‐body measurements suggests this method may provide more consistent results for visceral fat quantification.
**Appendix S43:** Forest plot evaluating comorbidity subgroups (No [*n* = 556] vs. Yes [*n* = 158]) across eight studies. Significant differences were found (*p* = 0.001), with non‐comorbid patients showing higher visceral fat area (119.60 cm^2^, 95% CI: 88.30–150.91; *I* ^2^ = 97.8%) versus comorbid patients (83.34 cm^2^, 95% CI: 61.87–104.81; *I* ^2^ = 87%). The extreme overall heterogeneity (*I* ^2^ = 98.3%) suggests substantial variability in adiposity measurements between studies.
**Appendix S44:** Forest plot comparing imaging modalities (DXA [*n* = 258], CT [*n* = 214], and MRI [*n* = 242]) across eight studies. Significant differences were found (*p* = 0.022), with CT showing the widest variability (59.50–140.20 cm^2^; *I* ^2^ = 98.5%) and MRI demonstrating the lowest heterogeneity (*I* ^2^ = 68.6%). DXA measurements ranged from 107.70 to 147.00 cm^2^ (*I* ^2^ = 90%), highlighting modality‐specific measurement characteristics.
**Appendix S45:** Sensitivity analysis of subcutaneous fat volume (*n* = 13 studies) demonstrates robust pooled estimates (22,536 cm^3^, 95% CI: 12,804–32,268) maintained across all study omissions (range: 19,777–24,128 cm^3^). Extreme heterogeneity persists throughout all iterations (*I* ^2^ = 99.9%, τ^2^ = 113,896,843), confirming the stability of findings despite substantial between‐study variability in adiposity measurements.
**Appendix S46:** Sensitivity analysis of subcutaneous fat area (*n* = 10 studies) shows consistent pooled estimates (312 cm^2^, 95% CI: 195–430) across all exclusions (range: 289–340 cm^2^). The persistent extreme heterogeneity (*I* ^2^ = 100%) across all analyses underscores the need for standardized measurement protocols in body composition research.
**Appendix S47:** Sensitivity analysis of visceral fat volume (*n* = 13 studies) reveals stable pooled estimates (3615 cm^3^, 95% CI: 1487–5743) throughout all study omissions (range: 3022–4748 cm^3^). The universal extreme heterogeneity (*I* ^2^ = 100%) highlights significant methodological variability in visceral adiposity quantification across studies.
**Appendix S48:** Forest plot evaluating visceral fat area estimates (*n* = 10 studies). The analysis revealed remarkable stability (range: 86.52–104.01 cm^2^) around the pooled estimate of 92.69 cm^2^ (95% CI: 61.36–124.01), with all confidence intervals overlapping. The exclusion of Froelich et al. (2020) notably reduced heterogeneity (*I* ^2^ = 98.1% vs. 99.9% overall), suggesting this study may contribute disproportionately to between‐study variability in visceral fat area measurements.
**Appendix S49:** Forest plot demonstrating the robustness of total fat volume estimates (*n* = 13 studies) when sequentially omitting individual studies. The pooled estimate remained stable (range: 30,072.65–37,760.37 cm^3^) despite extreme heterogeneity (*I* ^2^ ≥ 99.6% in all iterations). The overall estimate of 35,062.32 cm^3^ (95% CI: 16,041.66–54,082.98) showed < 15% variation when any single study was excluded, confirming the analysis was not unduly influenced by individual studies. The consistently high τ^2^ values (> 96 million across all iterations) highlight substantial between‐study variability regardless of study exclusion.
**Appendix S50:** Forest plot assessing the stability of total fat area estimates (*n* = 9 studies). The pooled estimates varied modestly (range: 387.25–434.25 cm^2^) when excluding individual studies, consistently overlapping with the overall estimate of 411.12 cm^2^ (95% CI: 289.90–532.35). Heterogeneity remained extremely high (*I* ^2^ ≥ 97.1%) in all iterations, though τ^2^ values (14,664.27–17,970.58) were substantially lower than for fat volume analyses, suggesting greater relative consistency in area measurements.
**Appendix S51:** Quantitative evaluation using Egger's linear regression (*p* = 0.032) and Begg's rank correlation (*p* = 0.047) tests for adiposity metrics.
**Appendix S52:** Trim‐and‐fill adjusted funnel plot for subcutaneous fat volume (SFV) shows asymmetric distribution of effect sizes, particularly in the high standard error range (> 2000). The plot indicates potential publication bias with missing studies likely in the low effect size (< 20,000 cm^3^) and high variability quadrant, implying underrepresentation of smaller null studies.
**Appendix S53:** Trim‐and‐fill adjusted funnel plot for visceral fat area (VFA) demonstrates significant asymmetry across the full spectrum of spread error values (0–726), with imputed studies concentrated in high variability regions (> 100 spread error). This pattern reveals substantial publication bias, suggesting systematic exclusion of studies with higher measurement variability from the literature.

**Table S1:** PRISMA 2020 checklist of the systematic review and meta‐analysis.
**Table S2:** Comprehensive search strategies for radiomics and obesity research across multiple databases.
**Table S3:** Grey literature sources searched for adiposity and radiomics evidence.
**Table S4:** Multimodality imaging protocols and technical parameters for adiposity quantification across studies (1990–2025).
**Table S5:** Quantitative evaluation using Egger's linear regression (*p* = 0.032) and Begg's rank correlation (*p* = 0.047) tests for adiposity metrics.
**Figure S1:** Bland–Altman plot comparing automated versus conventional methods for fat volume measurement shows a mean bias of −22.3 cm^3^ (95% limits of agreement: −854.94 to 810.34), indicating no systematic measurement bias between methods despite wide variability in individual measurements (range: 0–30,000 cm^3^).
**Figure S2:** Forest plot displays subgroup analysis of total fat volume (TFV) by age categories (median age > 47.1 vs. ≤ 47.1 years), revealing no significant differences between age groups (*p* = 0.854) despite extreme heterogeneity in both subgroups (*I* ^2^ = 99.8%). Younger participants showed wider TFV range (11,308–49,150 cm^3^) compared to older participants (4897–79,684 cm^3^).
**Figure S3:** Forest plot presents subgroup analysis of total fat volume (TFV) by body region (regional vs. whole‐body), showing no significant differences between measurement approaches (*p* = 0.939) despite extreme heterogeneity in both subgroups (*I* ^2^ = 99.6%–99.9%). Regional measurements demonstrated wider TFV range (4897–149,383 cm^3^) compared to whole‐body assessments (20,344–49,150 cm^3^).
**Figure S4:** Forest plot displays subgroup analysis of TFV by comorbidity status, revealing no significant differences between groups with and without comorbidities (*p* = 0.756), though comorbidity‐positive studies showed greater heterogeneity (*I* ^2^ = 99.9% vs. 98.5%). The comorbidity group included both the lowest (4897 cm^3^) and highest (149,383 cm^3^) observed TFV values.
**Figure S5:** Forest plot illustrates subgroup analysis of TFV by imaging modality (DXA/MRI/CT), demonstrating significant differences between modalities (*p* = 0.003) with MRI studies showing the widest TFV range (11,308–149,383 cm^3^) and highest heterogeneity (*I* ^2^ = 99.8%, τ^2^ = 398,752,853). DXA measurements were consistently higher (32,769–79,684 cm^3^) than CT values (4897–20,344 cm^3^).
**Figure S6:** Forest plot of total fat volume (TFV) across 13 studies (*n* = 1326) stratified by obesity status (Obese, Overweight). Extreme heterogeneity was noted overall (*I* ^2^ = 99.8%) and within subgroups (Obese: *I* ^2^ = 99.9%; Overweight: *I* ^2^ = 99.1%). TFV ranged from 4897 cm^3^ (Mourtzakis et al. 2008) to 149,383 cm^3^ (Positano et al. 2004). The pooled estimate was 35,062 cm^3^ (95% CI: 27,328–42,797), with a prediction interval of 16,042–54,083 cm^3^. Subgroup differences were significant (*p* = 0.048).
**Figure S7:** Forest plot of total fat area (TFA) across nine studies (*n* = 952). Extreme heterogeneity was observed (*I* ^2^ = 98.9%), with means ranging from 232.79 cm^2^ (Kim et al. 2019) to 643.00 cm^2^ (Ross et al. 1993). The pooled estimate was 411.12 cm^2^ (95% CI: 289.90–532.35), and the prediction interval (94.16–728.09 cm^2^) underscored high between‐study variability.
**Figure S8:** Forest plot of total fat area across eight studies (*n* = 932) stratified by age (median age ≤ 47.1 vs. > 47.1 years). Extreme heterogeneity was observed overall (*I* ^2^ = 99.0%) and within subgroups (> 47.1: *I* ^2^ = 95.2%; ≤ 47.1: *I* ^2^ = 98.3%). The pooled estimate was 434.25 cm^2^ (95% CI: 338.52–529.97), with older participants showing lower but nonsignificant differences (subgroup *p* = 0.374).
**Figure S9:** Forest plot of TFA across nine studies (*n* = 952) comparing regional vs. whole‐body measurements. Extreme heterogeneity persisted overall (*I* ^2^ = 98.9%) and within subgroups (Regional: *I* ^2^ = 97.5%; Whole‐body: *I* ^2^ = 95.8%). The pooled estimate was 411.12 cm^2^ (95% CI: 323.64–498.61), with no significant subgroup differences (*p* = 0.655). Notably, whole‐body studies showed wider variability (e.g., 317.88 to 643.00 cm^2^ vs. 232.79–574.90 cm^2^ for regional).
**Figure S10:** Forest plot of total fat area (TFA) across nine studies (*n* = 741) stratified by comorbidity status. Significant subgroup differences were detected (*p* = 0.0006), with non‐comorbid participants showing higher pooled estimates (506.62 cm^2^, 95% CI: 472.73–540.51) and lower heterogeneity (*I* ^2^ = 66.5%) compared to comorbid groups (320.84 cm^2^, 95% CI: 220.68–421.01; *I* ^2^ = 96.6%). Extreme overall heterogeneity was observed (*I* ^2^ = 98.9%).
**Figure S11:** Forest plot of TFA across nine studies (*n* = 952) stratified by imaging modality. Significant subgroup differences were observed (*p* = 0.021), with DXA showing the most consistent measurements (*I* ^2^ = 81.7%, pooled estimate = 514.07 cm^2^ [95% CI: 464.40–563.74]). Extreme heterogeneity was noted in CT (*I* ^2^ = 97.6%) and MRI (*I* ^2^ = 97.1%) subgroups, with MRI demonstrating the widest variability (233.41–643.00 cm^2^). The overall pooled estimate was 411.12 cm^2^ (95% CI: 323.64–498.61) with extreme heterogeneity (*I* ^2^ = 98.9%).
**Figure S12:** Forest plot of TFA across nine studies comparing obese (*n* = 397) and overweight (*n* = 137) populations. No significant subgroup differences were found (*p* = 0.752), despite extreme heterogeneity in both groups (obese: *I* ^2^ = 97.3%; overweight: *I* ^2^ = 98.9%). The obese subgroup showed a pooled estimate of 401.27 cm^2^ (95% CI: 302.45–500.09), while overweight studies demonstrated wider variability (317.88–574.90 cm^2^). The overall estimate was 411.12 cm^2^ (95% CI: 323.64–498.61).
**Figure S13:** Forest plot of TFA across seven studies (*n* = 854) stratified by waist circumference (median in meter ≤ 0.95 vs. > 0.95). No subgroup differences were detected (*p* = 0.944), though both groups showed extreme heterogeneity (≤ 0.95: *I* ^2^ = 99.5%; > 0.95: *I* ^2^ = 96.2%). The ≤ 0.95 subgroup had a pooled estimate of 450.05 cm^2^ (95% CI: 320.02–580.09), while the > 0.95 subgroup showed paradoxical results (232.79–643.00 cm^2^). The overall estimate was 442.87 cm^2^ (95% CI: 333.90–551.85).
**Figure S14:** Forest plot comparing subcutaneous fat volume stratified by body weight (> 75 kg, *n* = 391) vs. below the median value (≤ 74.9, *n* = 308) populations across eight studies (*n* = 699). Significant subgroup differences were found (*p* = 0.0005), with high weight participants showing higher pooled volumes (34,155.82 cm^3^, 95% CI: 27,772.25–40,539.39) compared to participants having lower weight (24,129.60–28,200.00 cm^3^). Extreme heterogeneity was observed in both subgroups (> 75 kg: *I* ^2^ = 98.4%; ≤ 74.9: *I* ^2^ = 99.6%) and overall (*I* ^2^ = 99.9%).
**Figure S15:** Forest plot of seven studies (*n* = 594) stratified by height above and below the median value in meter (> 1.60 vs. ≤ 1.60). Significant subgroup differences emerged (*p* = 0.0099), with higher height groups demonstrating greater fat volumes (34,155.82 cm^3^, 95% CI: 27,772.25–40,539.39) versus lower height groups (25,173.32 cm^3^, 95% CI: 22,756.29–27,590.35). Notably, heterogeneity was substantially lower in the ≤ 1.60 subgroup (*I* ^2^ = 80.8% vs. 98.1%).
**Figure S16:** Forest plot of 12 studies (*n* = 995) significant differences in subcutaneous fat volume (SFV) comparing MRI (*n* = 705), CT (*n* = 101), and DXA (*n* = 189) measurements. Significant modality effects were found (*p* = 0.0241), with MRI showing the widest range (3524.50–41,100.00 cm^3^) and highest heterogeneity (*I* ^2^ = 99.9%). DXA measurements demonstrated the lowest heterogeneity (*I* ^2^ = 80.8%). The overall estimate was 31,168.37 cm^3^ (95% CI: 23,351.11–38,985.64) with extreme heterogeneity (*I* ^2^ = 99.9%).
**Figure S17:** Forest plot presents subgroup analysis of subcutaneous fat volume (SFV) by age categories (≤ 47.1 vs. > 47.1 years), showing no significant difference between age groups (*p* = 0.090) despite extreme heterogeneity in both subgroups (*I* ^2^ = 100% and 99.8%, respectively), with younger participants demonstrating wider SFV range (3524–41,100 cm^3^) compared to older participants (3647–24,130 cm^3^).
**Figure S18:** Forest plot displays subgroup analysis of SFV by body mass percentage (BMP) (≤ 35% vs. > 35%) revealing no significant subgroup differences (*p* = 0.780) with persistent extreme heterogeneity (*I* ^2^ = 100%) across all studies, though higher BMP group showed greater variability in SFV measurements (5905–35,120 cm^3^ vs. 3525–41,100 cm^3^).
**Figure S19:** Forest plot illustrates subgroup analysis of SFV by body region (regional vs. whole‐body), demonstrating no significant measurement differences (*p* = 0.121) despite substantial heterogeneity in both subgroups (*I* ^2^ = 99.9%), with whole‐body assessments showing higher mean SFV (29,093 cm^3^) compared to regional measurements (15,111 cm^3^).
**Figure S20:** Forest plot presents subgroup analysis of subcutaneous fat volume (SFV) by comorbidity status, showing no significant difference between groups with and without comorbidities (*p* = 0.236), with similarly extreme heterogeneity in both subgroups (*I* ^2^ = 100% vs. 99%) and comparable SFV ranges (3647–41,100 cm^3^ with comorbidities vs. 5905–35,120 cm^3^ without).
**Figure S21:** Forest plot displays subgroup analysis of SFV by obesity status (overweight vs. obese), revealing no significant differences between groups (*p* = 0.968) despite extreme heterogeneity in both subgroups (*I* ^2^ = 99.9%), with obese individuals showing wider SFV range (3647–41,100 cm^3^) compared to overweight (3525–35,120 cm^3^).
**Figure S22:** Forest plot of subcutaneous fat area across 10 studies (*n* = 1081) demonstrating extreme heterogeneity (*I* ^2^ = 100%). Individual study means ranged from 26.50 cm^2^ (Hexsel et al. 2022) to 543.50 cm^2^ (Ross et al. 1993), with a pooled estimate of 312.22 cm^2^ (95% CI: 194.72–429.73). The wide prediction interval (−89.65 to 714.10 cm^2^) highlights substantial between‐study variability in measurement techniques or populations.
**Figure S23:** Forest plot comparing subcutaneous fat area across imaging modalities. Subcutaneous fat area varied substantially depending on the imaging technique. DXA studies (*n* = 258) yielded a pooled mean of 386.02 cm^2^ (95% CI 328.07–443.97; *I* ^2^ = 94.4%), while MRI studies (*n* = 164) produced a markedly imprecise pooled estimate of 348.68 cm^2^ (95% CI −34.95–732.32; *I* ^2^ = 99%). In contrast, CT studies (*n* = 659) showed a lower pooled mean of 235.83 cm^2^ (95% CI 152.66–318.99; *I* ^2^ = 97.8%). The overall random‐effects model across all modalities indicated a pooled mean of 312.22 cm^2^ (95% CI 206.14–418.30; *I* ^2^ = 100%). A test for subgroup differences was statistically significant (χ^2^ = 8.44, df = 2, *p* = 0.0147), suggesting that imaging modality explains part of the variability. Nonetheless, extreme heterogeneity persisted, underscoring the influence of methodological differences and population characteristics on subcutaneous fat measurements.
**Figure S24:** Forest plot of eight studies (*n* = 615) stratified by body fat percentage (> 35% vs. ≤ 35%). Significant differences emerged (*p* = 0.011), with higher adiposity groups demonstrating greater subcutaneous fat areas (pooled mean range: 330.70–543.50 cm^2^, *I* ^2^ = 90.5%) versus lower adiposity groups (26.50–238.64 cm^2^, *I* ^2^ = 100%). The extreme overall heterogeneity (*I* ^2^ = 100%) underscores the influence of body composition on measurement outcomes.
**Figure S25:** Forest plot of subcutaneous fat area across 10 studies (*n* = 1086) comparing regional (*n* = 975) versus whole‐body (*n* = 111) measurements. While no significant subgroup differences were found (*p* = 0.297), regional measurements showed greater variability (*I* ^2^ = 99.9%, range: 26.50–467.20 cm^2^) compared to whole‐body assessments (*I* ^2^ = 96.9%, range: 238.64–543.50 cm^2^). The pooled estimate was 312.22 cm^2^ (95% CI: 206.14–418.30) with extreme overall heterogeneity (*I* ^2^ = 100%), suggesting measurement methodology may influence results despite nonsignificant subgroup effects.
**Figure S26:** Forest plot comparing obese (*n* = 393) and overweight (*n* = 270) populations across 10 studies. No significant differences were observed (*p* = 0.325), though obese individuals showed slightly higher pooled fat areas (334.64 cm^2^, 95% CI: 275.57–393.70) with lower heterogeneity (*I* ^2^ = 96.1%) compared to overweight groups (243.02 cm^2^, 95% CI: 70.22–415.82; *I* ^2^ = 100%). The extreme overall heterogeneity (*I* ^2^ = 100%) indicates additional unmeasured factors contribute to variability beyond obesity status.
**Figure S27:** Forest plot evaluating automated (*n* = 85) versus conventional (*n* = 996) segmentation techniques across 10 studies. Nonsignificant differences were found (*p* = 0.244), with automated methods showing marginally higher estimates (402.24 cm^2^, 95% CI: 252.93–551.55; *I* ^2^ = 91.9%) versus conventional approaches (289.22 cm^2^, 95% CI: 171.38–407.07; *I* ^2^ = 100%). The persistent extreme heterogeneity (*I* ^2^ = 100%) across all analyses highlights the need for standardized measurement protocols in adiposity research.
**Figure S28:** Forest plot evaluating waist circumference subgroups (≤ 0.95 m [*n* = 394] vs. > 0.95 m [*n* = 460]) across six studies. Nonsignificant differences were found (*p* = 0.924), with both subgroups showing similar estimates (≤ 0.95 m: mean not reported, 95% CI: not available, *I* ^2^ = 99.6% vs. > 0.95 m: mean not reported, 95% CI: not available, *I* ^2^ = 97.6%). The extreme heterogeneity (*I* ^2^ = 99.4%) across analyses indicates substantial variability in subcutaneous fat area measurements.
**Figure S29:** Forest plot analyzing visceral fat volume across 13 studies (*n* = 1070). Extreme heterogeneity was observed (*I* ^2^ = 100%), with individual study means ranging from 13.92 to 22,472.70 cm^3^. The random effects model yielded a pooled estimate of 3614.81 cm^3^ (95% CI: 1486.73–5742.89), with prediction interval (2721.90–4507.72 cm^3^) reflecting substantial between‐study variability.
**Figure S30:** Forest plot evaluating age subgroups (≤ 47.1 years [*n* = 669] vs. > 47.1 years [*n* = 327]) across 11 studies. Nonsignificant differences were found (*p* = 0.646), with younger participants showing higher estimates (4145.30 cm^3^, 95% CI: 1262.51–7028.09; *I* ^2^ = 100%) versus older participants (3440.00 cm^3^, 95% CI: 2579.48–4300.52; *I* ^2^ = 100%). The extreme heterogeneity (*I* ^2^ = 100%) persists across all analyses.
**Figure S31:** Forest plot of visceral fat volume across six studies (*n* = 599) stratified by waist circumference in meter (≤ 0.95 vs. > 0.95). Significant subgroup differences were observed (*p* < 0.0001), with the ≤ 0.95 group showing extreme variability (13.92–9702.00 cm^3^, *I* ^2^ = 100%) compared to the > 0.95 group (3345.00 cm^3^ single study). The pooled estimate was 3585.95 cm^3^ (95% CI: 3337.71–3834.20) with extreme overall heterogeneity (*I* ^2^ = 100%), suggesting WC may be an important modifier of visceral adiposity measurements.
**Figure S32:** Forest plot comparing whole‐body (*n* = 490) versus regional (*n* = 506) measurements across 11 studies. No significant differences were found (*p* = 0.976), though whole‐body measurements showed greater variability (13.92–8050.00 cm^3^, *I* ^2^ = 100%) versus regional assessments (52.65–9702.00 cm^3^, *I* ^2^ = 100%). The similar pooled estimates (whole‐body: 3883.64 cm^3^; regional: 3823.29 cm^3^) suggest measurement methodology may not substantially influence visceral fat quantification despite extreme heterogeneity (*I* ^2^ = 100%).
**Figure S33:** Forest plot of 11 studies (*n* = 996) stratified by comorbidity status. Nonsignificant differences emerged (*p* = 0.134), though comorbid patients showed lower pooled volumes (3140.77 cm^3^, 95% CI: 2840.66–3440.88; *I* ^2^ = 100%) versus non‐comorbid groups (4851.26 cm^3^, 95% CI: 2636.45–7066.07; *I* ^2^ = 99.6%). The extreme overall heterogeneity (*I* ^2^ = 100%) indicates additional factors beyond comorbidity status affect measurements.
**Figure S34:** Forest plot comparing whole‐body (*n* = 490) versus regional (*n* = 506) assessment methods across 11 studies. No significant differences were found (*p* = 0.976), with similar pooled estimates (whole‐body: 3883.64 cm^3^; regional: 3823.29 cm^3^) despite extreme heterogeneity in both subgroups (*I* ^2^ = 100%). The remarkably wide range of values (13.92–9702.00 cm^3^ across all studies) highlights substantial variability in visceral fat quantification regardless of measurement approach.
**Figure S35:** Forest plot of visceral fat volume across 12 studies (*n* = 996) comparing obese (*n* = 465) versus overweight (*n* = 531) populations. No significant subgroup differences were observed (*p* = 0.463), though obese individuals showed higher pooled volumes (4407.97 cm^3^, 95% CI: 3463.06–5352.88) compared to overweight groups (3492.95 cm^3^, 95% CI: 1237.10–5748.80). Extreme heterogeneity was present in both subgroups (*I* ^2^ = 100%) and overall (*I* ^2^ = 100%), with values ranging from 13.92 to 9702.00 cm^3^, suggesting obesity status alone does not explain measurement variability.
**Figure S36:** Forest plot of visceral fat area across eight studies (*n* = 714) stratified by obesity status. Significant subgroup differences were observed (*p* = 0.016), with obese individuals demonstrating higher visceral adiposity (117.21 cm^2^, 95% CI: 93.57–140.84; *I* ^2^ = 93.5%) compared to overweight subjects (85.82 cm^2^, 95% CI: 75.88–95.76; *I* ^2^ = 84.4%). The overall pooled estimate was 105.95 cm^2^ (95% CI: 83.39–128.51) with extreme heterogeneity (*I* ^2^ = 98.3%), suggesting obesity status significantly influences visceral fat accumulation but does not fully explain measurement variability.
**Figure S37:** Forest plot comparing older (> 47.1 years, *n* = 335) versus younger (≤ 47.1 years, *n* = 379) populations. Significant differences emerged (*p* = 0.016), with older individuals showing greater visceral fat accumulation (121.59 cm^2^, 95% CI: 96.31–146.87; *I* ^2^ = 93.7%) versus younger subjects (88.13 cm^2^, 95% CI: 78.38–97.88; *I* ^2^ = 82.3%). The persistent high heterogeneity (*I* ^2^ = 98.3% overall) indicates age‐related differences in visceral adiposity, though other factors likely contribute to measurement variability.
**Figure S38:** Forest plot of eight studies (*n* = 714) comparing regional (*n* = 392) versus whole‐body (*n* = 322) assessment methods. Highly significant differences were found (*p* = 0.002), with regional measurements yielding higher estimates (118.83 cm^2^, 95% CI: 96.27–141.40; *I* ^2^ = 93.3%) versus whole‐body approaches (81.38 cm^2^, 95% CI: 75.61–87.14; *I* ^2^ = 54.7%). The substantially lower heterogeneity in whole‐body measurements suggests this method may provide more consistent results for visceral fat quantification.
**Figure S39:** Forest plot evaluating comorbidity subgroups (No [*n* = 556] vs. Yes [*n* = 158]) across eight studies. Significant differences were found (*p* = 0.001), with non‐comorbid patients showing higher visceral fat area (119.60 cm^2^, 95% CI: 88.30–150.91; *I* ^2^ = 97.8%) versus comorbid patients (83.34 cm^2^, 95% CI: 61.87–104.81; *I* ^2^ = 87%). The extreme overall heterogeneity (*I* ^2^ = 98.3%) suggests substantial variability in adiposity measurements between studies.
**Figure S40:** Forest plot comparing imaging modalities (DXA [*n* = 258], CT [*n* = 214], MRI [*n* = 242]) across eight studies. Significant differences were found (*p* = 0.022), with CT showing the widest variability (59.50–140.20 cm^2^; *I* ^2^ = 98.5%) and MRI demonstrating the lowest heterogeneity (*I* ^2^ = 68.6%). DXA measurements ranged from 107.70 to 147.00 cm^2^ (*I* ^2^ = 90%), highlighting modality‐specific measurement characteristics.
**Figure S41:** Sensitivity analysis of subcutaneous fat volume (*n* = 13 studies) demonstrates robust pooled estimates (22,536 cm^3^, 95% CI: 12,804–32,268) maintained across all study omissions (range: 19,777–24,128 cm^3^). Extreme heterogeneity persists throughout all iterations (*I* ^2^ = 99.9%, τ^2^ = 113,896,843), confirming the stability of findings despite substantial between‐study variability in adiposity measurements.
**Figure S42:** Sensitivity analysis of subcutaneous fat area (*n* = 10 studies) shows consistent pooled estimates (312 cm^2^, 95% CI: 195–430) across all exclusions (range: 289–340 cm^2^). The persistent extreme heterogeneity (*I* ^2^ = 100%) across all analyses underscores the need for standardized measurement protocols in body composition research.
**Figure S43:** Sensitivity analysis of visceral fat volume (*n* = 13 studies) reveals stable pooled estimates (3615 cm^3^, 95% CI: 1487–5743) throughout all study omissions (range: 3022–4748 cm^3^). The universal extreme heterogeneity (*I* ^2^ = 100%) highlights significant methodological variability in visceral adiposity quantification across studies.
**Figure S44:** Forest plot evaluating visceral fat area estimates (*n* = 10 studies). The analysis revealed remarkable stability (range: 86.52–104.01 cm^2^) around the pooled estimate of 92.69 cm^2^ (95% CI: 61.36–124.01), with all confidence intervals overlapping. The exclusion of Froelich et al. (2020) notably reduced heterogeneity (*I* ^2^ = 98.1% vs. 99.9% overall), suggesting this study may contribute disproportionately to between‐study variability in visceral fat area measurements.
**Figure S45:** Forest plot demonstrating the robustness of total fat volume estimates (*n* = 13 studies) when sequentially omitting individual studies. The pooled estimate remained stable (range: 30,072.65–37,760.37 cm^3^) despite extreme heterogeneity (*I* ^2^ ≥ 99.6% in all iterations). The overall estimate of 35,062.32 cm^3^ (95% CI: 16,041.66–54,082.98) showed < 15% variation when any single study was excluded, confirming the analysis was not unduly influenced by individual studies. The consistently high τ^2^ values (> 96 million across all iterations) highlight substantial between‐study variability regardless of study exclusion.
**Figure S46:** Forest plot assessing the stability of total fat area estimates (*n* = 9 studies). The pooled estimates varied modestly (range: 387.25–434.25 cm^2^) when excluding individual studies, consistently overlapping with the overall estimate of 411.12 cm^2^ (95% CI: 289.90–532.35). Heterogeneity remained extremely high (*I* ^2^ ≥ 97.1%) in all iterations, though τ^2^ values (14,664.27–17,970.58) were substantially lower than for fat volume analyses, suggesting greater relative consistency in area measurements.
**Figure S47:** Trim‐and‐fill adjusted funnel plot for subcutaneous fat volume (SFV) shows asymmetric distribution of effect sizes, particularly in the high standard error range (> 2000). The plot indicates potential publication bias with missing studies likely in the low effect size (< 20,000 cm^3^) and high variability quadrant, implying underrepresentation of smaller null studies.
**Figure S48:** Trim‐and‐fill adjusted funnel plot for visceral fat area (VFA) demonstrates significant asymmetry across the full spectrum of spread error values (0–726), with imputed studies concentrated in high variability regions (> 100 spread error). This pattern reveals substantial publication bias, suggesting systematic exclusion of studies with higher measurement variability from the literature.

## Data Availability

Deidentified data are available upon request from corresponding author. No additional documents.
