# Supplementary material for: Precision or Paradox? AI‐Driven Adiposity Imaging in Women With Overweight and Obesity: A Systematic Review and Meta‐Analysis
Source: Obes Rev. 2026 Jul 8;27(9):e70187. doi: 10.1111/obr.70187 (PMC13460875; doi:10.1111/obr.70187)

Precision or Paradox? AI-Driven Adiposity Imaging in Women with Overweight and Obesity: A Systematic Review and Meta-Analysis

Asefa Adimasu TADDESE[1,] and Bjorn T. Tam[1,2*]

[1]Academy of Wellness and Human Development, Faculty of Arts and Social Sciences, Hong Kong Baptist University, Hong Kong SAR, China

[2]Dr. Stephen Hui Research Centre for Physical Recreation and Wellness, Faculty of Arts and Social Sciences, Hong Kong Baptist University, Hong Kong SAR, China

[*]**Corresponding Author:**

Bjorn T. Tam Email: bjorntam@hkbu.edu.hk

Supplementary Information

List of supplementary tables

Table S 1: PRISMA 2020 Checklist of the Systematic Review and Meta-Analysis. 8
Table S 2: Comprehensive Search Strategies for Radiomics and Obesity Research Across Multiple Databases 13
Table S 3. Grey Literature Sources Searched for Adiposity and Radiomics Evidence. 15
Table S 4: Multimodality imaging protocols and technical parameters for adiposity quantification across studies (1990–2025) 19
Table S 5: Quantitative evaluation using Egger's linear regression (p=0.032) and Begg's rank correlation (p=0.047) tests for adiposity metrics. 71

List of supplementary figures

Fig. S1: Bland-Altman plot comparing automated versus conventional methods for fat volume measurement shows a mean bias of -22.3 cm³ (95% limits of agreement: -854.94 to 810.34), indicating no systematic measurement bias between methods despite wide variability in individual measurements (range: 0-30000 cm³). 25
Fig. S 2: Forest plot displays subgroup analysis of total fat volume (TFV) by age categories (Median age >47.1 vs ≤47.1 years), revealing no significant differences between age groups (p=0.854) despite extreme heterogeneity in both subgroups (I²=99.8%). Younger participants showed wider TFV range (11,308-49,150 cm³) compared to older participants (4,897-79,684 cm³). 26

Fig. S 3: Forest plot presents subgroup analysis of total fat volume (TFV) by body region (regional vs whole-body), showing no significant differences between measurement approaches (p=0.939) despite extreme heterogeneity in both subgroups (I^2=99.6-99.9%). Regional measurements demonstrated wider TFV range (4,897-149,383 cm^3) compared to whole-body assessments (20,344-49,150 cm^3)................... 27

Fig. S 4 : Forest plot displays subgroup analysis of TFV by comorbidity status, revealing no significant differences between groups with and without comorbidities (p=0.756), though comorbidity-positive studies showed greater heterogeneity (I^2=99.9% vs 98.5%). The comorbidity group included both the lowest (4,897 cm^3) and highest (149,383 cm^3) observed TFV values. 28

Fig. S 5: Forest plot illustrates subgroup analysis of TFV by imaging modality (DXA/MRI/CT), demonstrating significant differences between modalities (p=0.003) with MRI studies showing the widest TFV range (11,308-149,383 cm^3) and highest heterogeneity (I^2=99.8%, τ^2=398,752,853). DXA measurements were consistently higher (32,769-79,684 cm^3) than CT values (4,897-20,344 cm^3). 29

Fig. S 6 : Forest plot of total fat volume (TFV) across 13 studies (n=1326) stratified by obesity status (Obese, Overweight). Extreme heterogeneity was noted overall (I^2=99.8%) and within subgroups (Obese: I^2=99.9%; Overweight: I^2=99.1%). TFV ranged from 4,897 cm^3 (Mourtzakis et al., 2008) to 149,383 cm^3 (Positano et al., 2004). The pooled estimate was 35,062 cm^3 (95% CI: 27,328–42,797), with a prediction interval of 16,042–54,083 cm^3. Subgroup differences were significant (p=0.048). 30

Fig. S 7: Forest plot of total fat area (TFA) across 9 studies (n=952). Extreme heterogeneity was observed (I^2=98.9%), with means ranging from 232.79 cm^2 (Kim et al., 2019) to 643.00 cm^2 (Ross et al., 1993). The pooled estimate was 411.12 cm^2 (95% CI: 289.90–532.35), and the prediction interval (94.16–728.09 cm^2) underscored high between-study variability.. 31

Fig. S 8: Forest plot of total fat area across 8 studies (n=932) stratified by age (Median age ≤47.1 vs. >47.1 years). Extreme heterogeneity was observed overall (I^2=99.0%) and within subgroups (>47.1: I^2=95.2%; ≤47.1: I^2=98.3%). The pooled estimate was 434.25 cm^2 (95% CI: 338.52–529.97), with older participants showing lower but non-significant differences (subgroup p=0.374).. 32

Fig. S 9: Forest plot of TFA across 9 studies (n=952) comparing regional vs. whole-body measurements. Extreme heterogeneity persisted overall (I^2=98.9%) and within subgroups (Regional: I^2=97.5%; Whole-body: I^2=95.8%). The pooled estimate was 411.12 cm^2 (95% CI: 323.64–498.61), with no significant subgroup differences (p=0.655). Notably, whole-body studies showed wider variability (e.g., 317.88 cm^2 to 643.00 cm^2 vs. 232.79–574.90 cm^2 for regional). 33

Fig. S 10: Forest plot of total fat area (TFA) across 9 studies (n=741) stratified by comorbidity status. Significant subgroup differences were detected (p=0.0006), with non-comorbid participants showing higher pooled estimates (506.62 cm^2, 95% CI: 472.73–540.51) and lower heterogeneity (I^2=66.5%) compared to comorbid groups (320.84 cm^2, 95% CI: 220.68–421.01; I^2=96.6%). Extreme overall heterogeneity was observed (I^2=98.9%). 34

Fig. S 11: Forest plot of TFA across 9 studies (n=952) stratified by imaging modality. Significant subgroup differences were observed (p=0.021), with DXA showing the most consistent measurements (I^2=81.7%, pooled estimate=514.07 cm^2 [95% CI: 464.40-563.74]). Extreme heterogeneity

was noted in CT (I^2=97.6%) and MRI (I^2=97.1%) subgroups, with MRI demonstrating the widest variability (233.41-643.00 cm^2). The overall pooled estimate was 411.12 cm^2 (95% CI: 323.64-498.61) with extreme heterogeneity (I^2=98.9%). 35

Fig. S 12: Forest plot of TFA across 9 studies comparing obese (n=397) and overweight (n=137) populations. No significant subgroup differences were found (p=0.752), despite extreme heterogeneity in both groups (obese: I^2=97.3%; overweight: I^2=98.9%). The obese subgroup showed a pooled estimate of 401.27 cm^2 (95% CI: 302.45-500.09), while overweight studies demonstrated wider variability (317.88-574.90 cm^2). The overall estimate was 411.12 cm^2 (95% CI: 323.64-498.61). 36

Fig. S 13: Forest plot of TFA across 7 studies (n=854) stratified by waist circumference (median in meter ≤0.95 vs >0.95). No subgroup differences were detected (p=0.944), though both groups showed extreme heterogeneity (≤0.95: I^2=99.5%; >0.95: I^2=96.2%). The ≤0.95 subgroup had a pooled estimate of 450.05 cm^2 (95% CI: 320.02-580.09), while the >0.95 subgroup showed paradoxical results (232.79-643.00 cm^2). The overall estimate was 442.87 cm^2 (95% CI: 333.90-551.85). 37

Fig. S 14: Forest plot comparing subcutaneous fat volume stratified by body weight (>75 kg., n=391) vs below the median value (≤74.9, n=308) populations across 8 studies (n=699). Significant subgroup differences were found (p=0.0005), with high weight participants showing higher pooled volumes (34,155.82 cm^3, 95% CI: 27,772.25-40,539.39) compared to participants having lower weight (24,129.60-28,200.00 cm^3). Extreme heterogeneity was observed in both subgroups ((>75 k.g: I^2=98.4%; ≤74.9: I^2=99.6%) and overall (I^2=99.9%). 38

Fig. S 15: Forest plot of 7 studies (n=594) stratified by height (above and below the median value in meter (>1.60 vs ≤1.60). Significant subgroup differences emerged (p=0.0099), with higher-height groups demonstrating greater fat volumes (34,155.82 cm^3, 95% CI: 27,772.25-40,539.39) versus lower-height groups (25,173.32 cm^3, 95% CI: 22,756.29-27,590.35). Notably, heterogeneity was substantially lower in the ≤1.60 subgroup (I^2=80.8% vs 98.1%). 39

Fig. S 16: Forest plot of 12 studies (n=995) significant differences in subcutaneous fat volume (SFV) comparing MRI (n=705), CT (n=101), and DXA (n=189) measurements. Significant modality effects were found (p=0.0241), with MRI showing the widest range (3,524.50-41,100.00 cm^3) and highest heterogeneity (I^2=99.9%). DXA measurements demonstrated the lowest heterogeneity (I^2=80.8%). The overall estimate was 31,168.37 cm^3 (95% CI: 23,351.11-38,985.64) with extreme heterogeneity (I^2=99.9%). 40

Fig. S 17: Forest plot presents subgroup analysis of subcutaneous fat volume (SFV) by age categories (≤47.1 vs >47.1 years), showing no significant difference between age groups (p=0.090) despite extreme heterogeneity in both subgroups (I^2=100% and 99.8% respectively), with younger participants demonstrating wider SFV range (3,524-41,100 cm^3) compared to older participants (3,647-24,130 cm^3). 41

Fig. S 18: Forest plot displays subgroup analysis of SFV by Body mass percentage(BMP) (≤35% vs >35%) revealing no significant subgroup differences (p=0.780) with persistent extreme heterogeneity (I^2=100%) across all studies, though higher BMP group showed greater variability in SFV measurements (5,905-35,120 cm^3 versus 3,525-41,100 cm^3). 42

Fig. S 19: Forest plot illustrates subgroup analysis of SFV by body region (regional vs whole-body), demonstrating no significant measurement differences (p=0.121) despite substantial heterogeneity in both subgroups (I^2=99.9%), with whole-body assessments showing higher mean SFV (29,093 cm^3) compared to regional measurements (15,111 cm^3). ... 43

Fig. S 20: Forest plot presents subgroup analysis of subcutaneous fat volume (SFV) by comorbidity status, showing no significant difference between groups with and without comorbidities (p=0.236), with similarly extreme heterogeneity in both subgroups (I^2=100% vs 99%) and comparable SFV ranges (3,647-41,100 cm^3 with comorbidities vs 5,905-35,120 cm^3 without). ... 44

Fig. S 21 : Forest plot displays subgroup analysis of SFV by obesity status (overweight vs obese), revealing no significant differences between groups (p=0.968) despite extreme heterogeneity in both subgroups (I^2=99.9%), with obese individuals showing wider SFV range (3,647-41,100 cm^3) compared to overweight (3,525-35,120 cm^3). ... 45

Fig. S 22: Forest plot of subcutaneous fat area across 10 studies (n=1081) demonstrating extreme heterogeneity (I^2=100%). Individual study means ranged from 26.50 cm^2 (Hexsel et al., 2022) to 543.50 cm^2 (Ross et al., 1993), with a pooled estimate of 312.22 cm^2 (95% CI: 194.72-429.73). The wide prediction interval (-89.65 to 714.10 cm^2) highlights substantial between-study variability in measurement techniques or populations. ... 46

Fig. S 23: Forest plot comparing subcutaneous fat area across imaging modalities. Subcutaneous fat area varied substantially depending on the imaging technique. DXA studies (n = 258) yielded a pooled mean of 386.02 cm^2 (95% CI 328.07–443.97; I^2 = 94.4%), while MRI studies (n = 164) produced a markedly imprecise pooled estimate of 348.68 cm^2 (95% CI −34.95–732.32; I^2 = 99%). In contrast, CT studies (n = 659) showed a lower pooled mean of 235.83 cm^2 (95% CI 152.66–318.99; I^2 = 97.8%). The overall random-effects model across all modalities indicated a pooled mean of 312.22 cm^2 (95% CI 206.14–418.30; I^2 = 100%). A test for subgroup differences was statistically significant (χ^2 = 8.44, df = 2, p = 0.0147), suggesting that imaging modality explains part of the variability. Nonetheless, extreme heterogeneity persisted, underscoring the influence of methodological differences and population characteristics on subcutaneous fat measurements. ... 47

Fig. S 24: Forest plot of 8 studies (n=615) stratified by body fat percentage (>35% vs ≤35%). Significant differences emerged (p=0.011), with higher adiposity groups demonstrating greater subcutaneous fat areas (pooled mean range: 330.70-543.50 cm^2, I^2=90.5%) versus lower adiposity groups (26.50-238.64 cm^2, I^2=100%). The extreme overall heterogeneity (I^2=100%) underscores the influence of body composition on measurement outcomes. ... 48

Fig. S 25: Forest plot of subcutaneous fat area across 10 studies (n=1086) comparing regional (n=975) versus whole-body (n=111) measurements. While no significant subgroup differences were found (p=0.297), regional measurements showed greater variability (I^2=99.9%, range: 26.50-467.20 cm^2) compared to whole-body assessments (I^2=96.9%, range: 238.64-543.50 cm^2). The pooled estimate was 312.22 cm^2 (95% CI: 206.14-418.30) with extreme overall heterogeneity (I^2=100%), suggesting measurement methodology may influence results despite non-significant subgroup effects. ... 49

Fig. S 26: Forest plot comparing obese (n=393) and overweight (n=270) populations across 10 studies. No significant differences were observed (p=0.325), though obese individuals showed slightly higher pooled fat areas (334.64 cm^2, 95% CI: 275.57-393.70) with lower heterogeneity (I^2=96.1%) compared to overweight groups (243.02 cm^2, 95% CI: 70.22-415.82; I^2=100%). The extreme overall heterogeneity (I^2=100%) indicates additional unmeasured factors contribute to variability beyond obesity status. 50

Fig. S 27: Forest plot evaluating automated (n=85) versus conventional (n=996) segmentation techniques across 10 studies. Non-significant differences were found (p=0.244), with automated methods showing marginally higher estimates (402.24 cm^2, 95% CI: 252.93-551.55; I^2=91.9%) versus conventional approaches (289.22 cm^2, 95% CI: 171.38-407.07; I^2=100%). The persistent extreme heterogeneity (I^2=100%) across all analyses highlights the need for standardized measurement protocols in adiposity research. 51

Fig. S 28: Forest plot evaluating waist circumference subgroups (≤0.95 m [n=394] vs >0.95 m [n=460]) across 6 studies. Non-significant differences were found (p=0.924), with both subgroups showing similar estimates (≤0.95 m: mean not reported, 95% CI: not available, I^2=99.6% vs >0.95 m: mean not reported, 95% CI: not available, I^2=97.6%). The extreme heterogeneity (I^2=99.4%) across analyses indicates substantial variability in subcutaneous fat area measurements. 52

Fig. S 29: Forest plot analysing visceral fat volume across 13 studies (n=1070). Extreme heterogeneity was observed (I^2=100%), with individual study means ranging from 13.92-22,472.70 cm^3. The random effects model yielded a pooled estimate of 3,614.81 cm^3 (95% CI: 1,486.73-5,742.89), with prediction interval (2,721.90-4,507.72 cm^3) reflecting substantial between-study variability. 53

Fig. S 30: Forest plot evaluating age subgroups (≤47.1 years [n=669] vs >47.1 years [n=327]) across 11 studies. Non-significant differences were found (p=0.646), with younger participants showing higher estimates (4,145.30 cm^3, 95% CI: 1,262.51-7,028.09; I^2=100%) versus older participants (3,440.00 cm^3, 95% CI: 2,579.48-4,300.52; I^2=100%). The extreme heterogeneity (I^2=100%) persists across all analyses. 54

Fig. S 31: Forest plot of visceral fat volume across 6 studies (n=599) stratified by waist circumference in meter (≤0.95 vs >0.95). Significant subgroup differences were observed (p<0.0001), with the ≤0.95 group showing extreme variability (13.92-9702.00 cm^3, I^2=100%) compared to the >0.95 group (3345.00 cm^3 single study). The pooled estimate was 3585.95 cm^3 (95% CI: 3337.71-3834.20) with extreme overall heterogeneity (I^2=100%), suggesting WC may be an important modifier of visceral adiposity measurements. 55

Fig. S 32: Forest plot comparing whole-body (n=490) versus regional (n=506) measurements across 11 studies. No significant differences were found (p=0.976), though whole-body measurements showed greater variability (13.92-8050.00 cm^3, I^2=100%) versus regional assessments (52.65-9702.00 cm^3, I^2=100%). The similar pooled estimates (whole-body: 3883.64 cm^3; regional: 3823.29 cm^3) suggest measurement methodology may not substantially influence visceral fat quantification despite extreme heterogeneity (I^2=100%). 56

Fig. S 33: Forest plot of 11 studies (n=996) stratified by comorbidity status. Non-significant differences emerged (p=0.134), though comorbid patients showed lower pooled volumes (3140.77 cm^3, 95% CI: 2840.66-3440.88; I^2=100%) versus non-comorbid groups (4851.26 cm^3, 95% CI: 2636.45-7066.07; I^2=99.6%). The extreme overall heterogeneity (I^2=100%) indicates additional factors beyond comorbidity status affect measurements. 57

Fig. S 34: Forest plot comparing whole-body (n=490) versus regional (n=506) assessment methods across 11 studies. No significant differences were found (p=0.976), with similar pooled estimates (whole-body: 3883.64 cm^3; regional: 3823.29 cm^3) despite extreme heterogeneity in both subgroups (I^2=100%). The remarkably wide range of values (13.92-9702.00 cm^3 across all studies) highlights substantial variability in visceral fat quantification regardless of measurement approach. 58

Fig. S 35: Forest plot of visceral fat volume across 12 studies (n=996) comparing obese (n=465) versus overweight (n=531) populations. No significant subgroup differences were observed (p=0.463), though obese individuals showed higher pooled volumes (4407.97 cm^3, 95% CI: 3463.06-5352.88) compared to overweight groups (3492.95 cm^3, 95% CI: 1237.10-5748.80). Extreme heterogeneity was present in both subgroups (I^2=100%) and overall (I^2=100%), with values ranging from 13.92 cm^3 to 9702.00 cm^3, suggesting obesity status alone does not explain measurement variability. 59

Fig. S 36: Forest plot of visceral fat area across 8 studies (n=714) stratified by obesity status. Significant subgroup differences were observed (p=0.016), with obese individuals demonstrating higher visceral adiposity (117.21 cm^2, 95% CI: 93.57-140.84; I^2=93.5%) compared to overweight subjects (85.82 cm^2, 95% CI: 75.88-95.76; I^2=84.4%). The overall pooled estimate was 105.95 cm^2 (95% CI: 83.39-128.51) with extreme heterogeneity (I^2=98.3%), suggesting obesity status significantly influences visceral fat accumulation but does not fully explain measurement variability. 60

Fig. S 37: Forest plot comparing older (>47.1 years, n=335) versus younger (≤47.1 years, n=379) populations. Significant differences emerged (p=0.016), with older individuals showing greater visceral fat accumulation (121.59 cm^2, 95% CI: 96.31-146.87; I^2=93.7%) versus younger subjects (88.13 cm^2, 95% CI: 78.38-97.88; I^2=82.3%). The persistent high heterogeneity (I^2=98.3% overall) indicates age-related differences in visceral adiposity, though other factors likely contribute to measurement variability. 61

Fig. S 38: Forest plot of 8 studies (n=714) comparing regional (n=392) versus whole-body (n=322) assessment methods. Highly significant differences were found (p=0.002), with regional measurements yielding higher estimates (118.83 cm^2, 95% CI: 96.27-141.40; I^2=93.3%) versus whole-body approaches (81.38 cm^2, 95% CI: 75.61-87.14; I^2=54.7%). The substantially lower heterogeneity in whole-body measurements suggests this method may provide more consistent results for visceral fat quantification. 62

Fig. S 39: Forest plot evaluating comorbidity subgroups (No [n=556] vs Yes [n=158]) across 8 studies. Significant differences were found (p=0.001), with non-comorbid patients showing higher visceral fat area (119.60 cm^2, 95% CI: 88.30-150.91; I^2=97.8%) versus comorbid patients (83.34 cm^2, 95% CI: 61.87-104.81; I^2=87%). The extreme overall heterogeneity (I^2=98.3%) suggests substantial variability in adiposity measurements between studies. 63

Fig. S 40: Forest plot comparing imaging modalities (DXA [n=258], CT [n=214], MRI [n=242]) across 8 studies. Significant differences were found (p=0.022), with CT showing the widest variability (59.50-140.20 cm^2; I^2=98.5%) and MRI demonstrating the lowest heterogeneity (I^2=68.6%). DXA measurements ranged from 107.70-147.00 cm^2 (I^2=90%), highlighting modality-specific measurement characteristics. 64

Fig. S 41: Sensitivity analysis of subcutaneous fat volume (n=13 studies) demonstrates robust pooled estimates (22,536 cm^3, 95% CI: 12,804-32,268) maintained across all study omissions (range: 19,777-24,128 cm^3). Extreme heterogeneity persists throughout all iterations (I^2=99.9%, τ^2=113,896,843), confirming the stability of findings despite substantial between-study variability in adiposity measurements. 65

Fig. S 42: Sensitivity analysis of subcutaneous fat area (n=10 studies) shows consistent pooled estimates (312 cm^2, 95% CI: 195-430) across all exclusions (range: 289-340 cm^2). The persistent extreme heterogeneity (I^2=100%) across all analyses underscores the need for standardized measurement protocols in body composition research. 66

Fig. S 43: Sensitivity analysis of visceral fat volume (n=13 studies) reveals stable pooled estimates (3,615 cm^3, 95% CI: 1,487-5,743) throughout all study omissions (range: 3,022-4,748 cm^3). The universal extreme heterogeneity (I^2=100%) highlights significant methodological variability in visceral adiposity quantification across studies. 67

Fig. S 44: Forest plot evaluating visceral fat area estimates (n=10 studies). The analysis revealed remarkable stability (range: 86.52-104.01 cm^2) around the pooled estimate of 92.69 cm^2 (95% CI: 61.36-124.01), with all confidence intervals overlapping. The exclusion of Froelich et al. (2020) notably reduced heterogeneity (I^2=98.1% vs 99.9% overall), suggesting this study may contribute disproportionately to between-study variability in visceral fat area measurements. 68

Fig. S 45: Forest plot demonstrating the robustness of total fat volume estimates (n=13 studies) when sequentially omitting individual studies. The pooled estimate remained stable (range: 30,072.65-37,760.37 cm^3) despite extreme heterogeneity ($I^2 \geq 99.6\%$ in all iterations). The overall estimate of 35,062.32 cm^3 (95% CI: 16,041.66-54,082.98) showed <15% variation when any single study was excluded, confirming the analysis was not unduly influenced by individual studies. The consistently high τ^2 values (>96 million across all iterations) highlight substantial between-study variability regardless of study exclusion. 69

Fig. S 46: Forest plot assessing the stability of total fat area estimates (n=9 studies). The pooled estimates varied modestly (range: 387.25-434.25 cm^2) when excluding individual studies, consistently overlapping with the overall estimate of 411.12 cm^2 (95% CI: 289.90-532.35). Heterogeneity remained extremely high ($I^2 \geq 97.1\%$) in all iterations, though τ^2 values (14,664.27-17,970.58) were substantially lower than for fat volume analyses, suggesting greater relative consistency in area measurements. 70

Fig. S 47: Trim-and-fill adjusted funnel plot for subcutaneous fat volume (SFV) shows asymmetric distribution of effect sizes, particularly in the high standard error range (>2000). The plot indicates potential publication bias with missing studies likely in the low effect size (<20,000 cm^3) and high variability quadrant, implying underrepresentation of smaller null studies. 72

Fig. S 48: Trim-and-fill adjusted funnel plot for visceral fat area (VFA) demonstrates significant asymmetry across the full spectrum of spread error values (0-726), with imputed studies concentrated in high variability regions (>100 spread error). This pattern reveals substantial publication bias, suggesting systematic exclusion of studies with higher measurement variability from the literature. 73

Table S 1: PRISMA 2020 Checklist of the Systematic Review and Meta-Analysis.

Section and Topic	Item #	Checklist item	Location where item is reported
**TITLE**			
Title	1	Identify the report as a systematic review.	Title Page, Page 1, Line 1
**ABSTRACT**			
Abstract	2	See the PRISMA 2020 for Abstracts checklist.	Abstract, Page 2, Lines 26–42
**INTRODUCTION**			
Rationale	3	Describe the rationale for the review in the context of existing knowledge.	Introduction, Pages 3–4, Lines 61–106
Objectives	4	Provide an explicit statement of the objective(s) or question(s) the review addresses.	Introduction, Page 4, Lines 107–113
**METHODS**			
Eligibility criteria	5	Specify the inclusion and exclusion criteria for the review and how studies were grouped for the syntheses.	Methods and Materials, pages 5–6, lines 128–153
Information sources	6	Specify all databases, registers, websites, organisations, reference lists and other sources searched or consulted to identify studies. Specify the date when each source was last searched or consulted.	Methods and Materials, pages 5–6, lines 128–153; Supplementary Tables S2–S3
Search strategy	7	Present the full search strategies for all databases, registers and websites, including any filters and limits used.	Methods and Materials, pages 5–6, lines 128–153; Supplementary Table S2
Selection process	8	Specify the methods used to decide whether a study met the inclusion criteria of the review, including how many reviewers screened each record and each report retrieved, whether they worked independently, and if applicable, details of automation tools used in the process.	Methods and Materials, pages 5–6, lines 154–163
Data collection process	9	Specify the methods used to collect data from reports, including how many reviewers collected data from each report, whether they worked independently, any processes for obtaining or confirming data from study investigators, and if applicable, details of automation tools used in the process.	Methods and Materials, pages 5–6, lines 154–163
Data items	10a	List and define all outcomes for which data were sought. Specify whether all results	Methods and Materials, pages

Section and Topic	Item #	Checklist item	Location where item is reported
		that were compatible with each outcome domain in each study were sought (e.g. for all measures, time points, analyses), and if not, the methods used to decide which results to collect.	5–6, lines 154–158
	10b	List and define all other variables for which data were sought (e.g. participant and intervention characteristics, funding sources). Describe any assumptions made about any missing or unclear information.	Methods and Materials, pages 5–6, lines 154–158; Supplementary Table S4
Study risk of bias assessment	11	Specify the methods used to assess risk of bias in the included studies, including details of the tool(s) used, how many reviewers assessed each study and whether they worked independently, and if applicable, details of automation tools used in the process.	Methods and Materials, pages 5–6, lines 158–163
Effect measures	12	Specify for each outcome the effect measure(s) (e.g. risk ratio, mean difference) used in the synthesis or presentation of results.	Methods and Materials, page 6, lines 165–187
Synthesis methods	13a	Describe the processes used to decide which studies were eligible for each synthesis (e.g. tabulating the study intervention characteristics and comparing against the planned groups for each synthesis (item #5)).	Methods and Materials, page 6, lines 165–187
	13b	Describe any methods required to prepare the data for presentation or synthesis, such as handling of missing summary statistics, or data conversions.	Methods and Materials, page 6, lines 165–187
	13c	Describe any methods used to tabulate or visually display results of individual studies and syntheses.	Methods and Materials, page 6, lines 165–187
	13d	Describe any methods used to synthesize results and provide a rationale for the choice(s). If meta-analysis was performed, describe the model(s), method(s) to identify the presence and extent of statistical heterogeneity, and software package(s) used.	Methods and Materials, page 6, lines 165–187
	13e	Describe any methods used to explore possible causes of heterogeneity among study results (e.g. subgroup analysis, meta-regression).	Methods and Materials, page 6, lines 165–187
	13f	Describe any sensitivity analyses conducted to assess robustness of the synthesized results.	Methods and Materials, page 6, lines 165–187
Reporting bias	14	Describe any methods used to assess risk of bias due to missing results in a synthesis (arising from reporting biases).	Methods and Materials, page 6, lines 165–187

Section and Topic	Item #	Checklist item	Location where item is reported
assessment			
Certainty assessment	15	Describe any methods used to assess certainty (or confidence) in the body of evidence for an outcome.	Methods and Materials, page 6, lines 165–187
**RESULTS**			
Study selection	16a	Describe the results of the search and selection process, from the number of records identified in the search to the number of studies included in the review, ideally using a flow diagram.	Results, page 7, lines 193–200; Figure 1
	16b	Cite studies that might appear to meet the inclusion criteria, but which were excluded, and explain why they were excluded.	Results, page 7, lines 201–208
Study characteristics	17	Cite each included study and present its characteristics.	Results, page 7, lines 201–245; Table 1; Supplementary Table S4
Risk of bias in studies	18	Present assessments of risk of bias for each included study.	Results, Table 2; Figures 2–4
Results of individual studies	19	For all outcomes, present, for each study: (a) summary statistics for each group (where appropriate) and (b) an effect estimate and its precision (e.g. confidence/credible interval), ideally using structured tables or plots.	Results, pages 7–8, lines 209–245; Figures 2–6; Supplementary Figures S2–S40
Results of syntheses	20a	For each synthesis, briefly summarise the characteristics and risk of bias among contributing studies.	Results, pages 8–10, lines 246–312; Tables 1–2
	20b	Present results of all statistical syntheses conducted. If meta-analysis was done, present for each the summary estimate and its precision (e.g. confidence/credible interval) and measures of statistical heterogeneity. If comparing groups, describe the direction of the effect.	Results, pages 7–10, lines 209–312; Figures 2–6; Supplementary Figures S2–S40
	20c	Present results of all investigations of possible causes of heterogeneity among study results.	Results, page 10, lines 293–312; Supplementary Figures S2–S40
	20d	Present results of all sensitivity analyses conducted to assess the robustness of the synthesized results.	Results, pages 10–11, lines 313–326; Supplementary

Section and Topic	Item #	Checklist item	Location where item is reported
			Figures S41–S46
Reporting biases	21	Present assessments of risk of bias due to missing results (arising from reporting biases) for each synthesis assessed.	Results, pages 10–11, lines 313–326; Supplementary Table S5; Supplementary Figures S47–S48
Certainty of evidence	22	Present assessments of certainty (or confidence) in the body of evidence for each outcome assessed.	
**DISCUSSION**			
Discussion	23a	Provide a general interpretation of the results in the context of other evidence.	Discussion, page 11 line 327-355
	23b	Discuss any limitations of the evidence included in the review.	Discussion, page 12 line 356-366
	23c	Discuss any limitations of the review processes used.	Discussion, page 12 line 367-379
	23d	Discuss implications of the results for practice, policy, and future research.	Discussion, page 12-13 line 380-409
**OTHER INFORMATION**			
Registration and protocol	24a	Provide registration information for the review, including register name and registration number, or state that the review was not registered.	Methods and Materials, page 6, lines 188–190
	24b	Indicate where the review protocol can be accessed, or state that a protocol was not prepared.	PROSPERO registration record (CRD420251026787)
	24c	Describe and explain any amendments to information provided at registration or in the protocol.	No amendments were made following protocol registration.
Support	25	Describe sources of financial or non-financial support for the review, and the role of the funders or sponsors in the review.	No specific funding was received for this study.
Competing interests	26	Declare any competing interests of review authors.	The authors declare no competing interests.
Availability of	27	Report which of the following are publicly available and where they can be found:	Deidentified data available

Section and Topic	Item #	Checklist item	Location where item is reported
data, code and other materials		template data collection forms; data extracted from included studies; data used for all analyses; analytic code; any other materials used in the review.	upon request from corresponding author. No additional documents.

From: Page MJ, McKenzie JE, Bossuyt PM, Boutron I, Hoffmann TC, Mulrow CD, et al. The PRISMA 2020 statement: an updated guideline for reporting systematic reviews. BMJ 2021;372:n71. doi: 10.1136/bmj.n71. This work is licensed under CC BY 4.0. To view a copy of this license, visit https://creativecommons.org/licenses/by/4.0/

Table S 2: Comprehensive Search Strategies for Radiomics and Obesity Research Across Multiple Databases

**Searching Terms**	
**Databases**	**Search strategy**
PubMed	*(("Radiomics"[Title/Abstract] OR "Imaging"[Title/Abstract] OR "CT-scan"[Title/Abstract] OR "CT scan"[Title/Abstract] OR "MRI"[Title/Abstract] OR "Magnetic resonance imaging"[Title/Abstract] OR "Medical imaging"[Title/Abstract] OR "Image-based"[Title/Abstract] OR "Quantitative imaging"[Title/Abstract] OR "Computational imaging"[Title/Abstract]) AND ("Obesity"[MeSH Terms] OR "Obesity"[Title/Abstract] OR "Abnormal obesity"[Title/Abstract] OR "Predicting obesity"[Title/Abstract] OR "Obesity biomarkers"[Title/Abstract] OR "Obesity risk factors"[Title/Abstract] OR "Metabolic Syndrome"[MeSH Terms] OR "Metabolism Disorders"[MeSH Terms] OR "Overweight"[MeSH Terms] OR "Overweight"[Title/Abstract] OR "Excess weight"[Title/Abstract] OR "Excessive fat"[Title/Abstract] OR "Body fat accumulation"[Title/Abstract] OR "Adiposity"[MeSH Terms] OR "Excess adiposity"[Title/Abstract] OR "Fat accumulation"[Title/Abstract] OR "High BMI"[Title/Abstract] OR "Body Mass Index"[MeSH Terms]) AND ("Female"[MeSH Terms] OR "Women"[Title/Abstract] OR "Gender-specific"[Title/Abstract]))*
Web of science	*TS=("Radiomics" OR "Imaging" OR "CT-scan" OR "CT scan" OR "MRI" OR "Magnetic resonance imaging" OR "Medical imaging" OR "Image-based" OR "Quantitative imaging" OR "Computational imaging") AND TS=("Obesity" OR "Abnormal obesity" OR "Predicting obesity" OR "Obesity biomarkers" OR "Obesity risk factors" OR "Metabolic Syndrome" OR "Metabolism Disorders" OR "Overweight" OR "Excess weight" OR "Excessive fat" OR "Body fat accumulation" OR "Adiposity" OR "Excess adiposity" OR "Fat accumulation" OR "High BMI" OR "Body Mass Index") AND TS=("Female" OR "Women" OR "Gender-specific")*
Scopus	*(TITLE-ABS-KEY ("Radiomics" OR "Imaging features" OR "Medical imaging" OR "Image-based" OR "Quantitative imaging" OR "CT scan" OR "MRI" OR "Magnetic resonance imaging" OR "CT-scan" OR "Computed tomography" OR "Computational imaging" OR "Ultrasound")) AND (TITLE-ABS-KEY ("Abnormal obesity" OR "Obesity" OR "Excess body fat" OR "Adiposity" OR "Excess adiposity" OR "Overweight" OR "Body Mass Index" OR "BMI" OR "Visceral fat" OR "Visceral adipose tissue" OR "Body composition" OR "Non-alcoholic fatty liver disease" OR "NAFLD" OR "Hepatic steatosis")) AND (TITLE-ABS-KEY ("Artificial Intelligence" OR "AI" OR "Machine Learning" OR "Deep Learning" OR "Neural Networks" OR "Computational techniques" OR "Automated analysis" OR "Radiomic techniques")) AND (TITLE-ABS-KEY ("Women" OR "Female" OR "Gender-specific" OR "Women's health"))*
Science Direct	*1) (radiomics OR "radiomic profiling") AND ("abnormal obesity" OR obesity OR adiposity) 2) TITLE("Radiomics" OR "Imaging" OR "CT scan" OR "MRI") AND TITLE("Obesity" OR "Abnormal obesity" OR "Predicting obesity") 3) TITLE("Magnetic resonance imaging" OR "Medical imaging" OR "Image-based") AND TITLE("Obesity biomarkers" OR "Obesity prediction" OR "Obesity risk factors") 4) TITLE("Quantitative imaging" OR "Computational imaging" OR "Radiomics and obesity prediction") AND TITLE("Obesity" OR "Metabolic disorders")*
Embase	*((Radiomics or "Imaging features" or "Medical imaging" or "Image-based" or "Quantitative imaging" or "CT scan" or MRI or "Magnetic resonance imaging" or "CT-scan" or "Computed tomography" or "Computational imaging" or Ultrasound or "Imaging biomarkers") and ("Abnormal obesity" or Obesity or "Excess body fat" or Adiposity or "Excess adiposity" or Overweight or "Body Mass Index" or BMI or "Visceral fat" or "Visceral adipose tissue" or "Body composition" or "Non-alcoholic fatty liver disease" or NAFLD or "Hepatic steatosis"*

	or "Metabolic disorder") and ("Artificial Intelligence" or AI or "Machine Learning" or "Deep Learning" or "Neural Networks" or "Computational techniques" or "Automated analysis" or "Radiomic techniques" or "Feature extraction") and (Women or Female or "Gender-specific" or "Women's health" or "Sex differences")).mp.
Cochrane Library	*1) "Radiomics" OR "Multi-omics" AND "Obesity 2) "Radiomics" AND ("Obesity" OR "Abnormal obesity" OR "Predicting obesity") 3) "Imaging" AND ("Obesity biomarkers" OR "Obesity prediction" OR "Obesity risk factors" 4) ("CT scan" OR "MRI") AND ("Obesity" OR "Metabolic disorders")*

Table S 3. Grey Literature Sources Searched for Adiposity and Radiomics Evidence.

Grey literature sources were systematically searched to capture emerging evidence not indexed in traditional databases. Conference proceedings (RSNA, MICCAI, ObesityWeek, CAIMI, and Obesity abstracts) were reviewed for early validation studies in radiomics and adipose tissue imaging. Preprint servers (medRxiv, arXiv) were interrogated using structured queries to identify recent advances in AI segmentation and adiposity phenotyping in women. Technical reports (World Obesity Atlas, IQVIA Outlook, Perspectum Imaging whitepapers) provided context on global prevalence, diagnostic acceleration, and imaging innovation. Additional grey literature, including doctoral theses, was incorporated to ensure comprehensive coverage of adipose tissue diversity and methodological advances.

Source / Venue	Type	Search Strategy	Representative Examples
RSNA (Radiological Society of North America)	Conference proceedings	*(AI segmentation) AND (fat quantification) AND (MRI OR CT OR DXA) AND (obesity)*	Murphy PM. *A Pair to Echo: AI Segmentation of CT and MRI.* Radiology. 2025;315(1):e250843.<br>Kartalis N, Grigoriadis A. *Photon-counting CT: The New Kid on the Block for Liver Fat Quantification.* Radiology. 2025;314(3):e250480.
MICCAI (Medical Image Computing and Computer Assisted Intervention)	Conference proceedings	*(AI segmentation) AND (fat quantification) AND (MRI OR CT OR DXA) AND (obesity)*	Ma Z et al. *AATCT-IDS benchmark abdominal adipose tissue CT dataset.* Comput Biol Med. 2024
ObesityWeek (The Obesity Society, TOS)	Conference proceedings	*(AI segmentation) AND (fat quantification) AND (MRI OR CT OR DXA) AND (obesity)*	*Poster Abstracts.* Obesity. 2024;32(S1):55-308.
CAIMI (SIIM Conference on AI in Medical Imaging)	Conference proceedings	*(AI segmentation) AND (fat quantification) AND (MRI OR CT OR DXA) AND (obesity)*	*Selected Abstracts from the 2025 Conference on Artificial Intelligence in Medical Imaging (CAIMI) of the Society for Imaging Informatics in Medicine (SIIM).* J Imaging Inform Med. 2025;38(Suppl 2):51-77.
medRxiv/ arXiv (eess.IV)	Preprint server	*(AI OR "artificial intelligence" OR "deep learning" OR "radiomics") AND (adiposity OR "adipose tissue" OR "visceral fat" OR "subcutaneous fat")*	1. Kim JN, Gomez-Perez L, Zimin VN, Makhlouf MHE, Al-Kindi S, Wilson DL, et al. Pericoronary adipose tissue radiomics from coronary CT angiography… medRxiv. 2023:2023.01.09.23284346.<br>2. Maldonado-Garcia C, Salih A, Neubauer S, Petersen SE, Raisi-Estabragh Z. Compartment-specific Fat Distribution Profiles… medRxiv. 2025:2025.09.16.25335879.

		AND (women OR female)	3. Morris DM, Wang C, Papanastasiou G, Gray CD, Badr S, Paccou J, et al. Deep learning for bone marrow adiposity… medRxiv. 2022:2022.12.06.22283151.<br>4. Pillai A, Bliznashki K, Hutchison E, Kumar C, Challis B, Patel M. Machine Learning Enabled Non-invasive Diagnosis of NAFLD… medRxiv. 2022:2022.03.25.22272965.<br>5. Wang G-J, Kuo L, Chang S-L, Lin Y-J, Chung F-P, Lo L-W, et al. Deep Learning-based Workflow for Automatic Extraction of Atria and Epicardial Adipose Tissue… medRxiv. 2023:2023.05.03.23289448.<br>6. Zhang M, Yin X, Li W, Zha Y, Zeng X, Zhang X, et al. Radiomics features from adrenal gland and periadrenal fat CT predicting COVID-19 progression. medRxiv. 2021:2021.01.03.21249183.<br>7. Agrawal S, Wang M, Klarqvist MDR, Shin J, Dashti H, Diamant N, et al. Inherited basis of visceral, abdominal subcutaneous and gluteofemoral fat depots. medRxiv. 2021:2021.08.24.21262564.<br>8. Alipour E, Gratzl S, Algohary A, Lin H, Bapat M, Do D, et al. Multimodal Deep Learning Model to Estimate CT-based Body Composition Measures… medRxiv. 2025:2025.01.16.25320684.<br>9. Baya NA, Erdem IS, Venkatesh SS, Reibe S, Charles PD, Navarro-Guerrero E, et al. Combining evidence from human genetic and functional screens… medRxiv. 2025:2024.09.19.24313913.<br>10. Brennan AM, Coen PM, Mau T, Hetherington-Rauth M, Toledo FGS, Kershaw EE, et al. Regional adipose tissue distribution and skeletal muscle bioenergetics… medRxiv. 2023:2023.11.10.23298359.<br>11. Kaufmann T, Bjørnstad PM, Falck M, Nerland S, O’Connell K, Frei O, et al. Estimating bone marrow adiposity from head MRI… medRxiv. 2024:2022.08.19.22278950.<br>12. Klarqvist MDR, Agrawal S, Diamant N, Ellinor PT, Philippakis A, Ng K, et al. Estimating body fat distribution from silhouette images. medRxiv. 2022:2022.01.14.22269328.<br>13. Losev V, Lu C, Senevirathne DS, Inglese P, Bai W, King AP, et al. Body fat and human cardiovascular ageing. medRxiv. 2024:2024.06.27.24309526.

			14. MacLean MT, Jehangir Q, Vujkovic M, Ko Y-A, Litt H, Borthakur A, et al. Linking abdominal imaging traits to EHR phenotypes. medRxiv. 2020:2020.09.08.20190330.<br>15. Parkinson JR, Thanaj M, Basty N, Whitcher B, Thomas EL, Bell JD. Fat fraction and iron concentration in lumbar vertebral bone marrow. medRxiv. 2025:2025.03.19.25324245.<br>16. Paul F, Wang H, Wang J, Tan LJ, Chia CY, Sun L, et al. CIDEC variant segregates with familial obesity… medRxiv. 2025:2025.07.28.25332029.<br>17. Thanaj M, Basty N, Whitcher B, Sorokin EP, Liu Y, Srinivasan R, et al. Precision MRI phenotyping of muscle volume and quality… medRxiv. 2023:2023.03.02.23286689.<br>18. Ma Z, Li C, Du T, Zhang L, Tang D, Ma D, et al. AATCT-IDS: Benchmark Abdominal Adipose Tissue CT Dataset. Comput Biol Med. 2024;177:108628.<br>19. Alghamdi M, Rashid M, Arif M. Systematic Review of AI Methods for Adipose Tissue Analysis in Medical Imaging. IEEE Access. 2026;PP:1-.
World Obesity Atlas (2020–2025)	Technical report	Keyword search: *obesity prevalence, NCD burden*	World Obesity Federation. *World Obesity Atlas 2024.*
IQVIA Obesity Outlook Reports	Technical report	Keyword search: *obesity care, diagnostic criteria*	IQVIA. *Obesity Outlook Report.* 2025
Perspectum Imaging Whitepapers	Technical report	Keyword search: *obesity imaging, metabolic disease*	Perspectum *Imaging*. *Obesity Imaging Whitepaper v2.* 2026
Doctoral Thesis (Karolinska Institutet)	Thesis (grey literature)	Keyword search: *white adipose tissue diversity, single-cell resolution*	Jalkanen J. Unveiling human white adipose tissue diversity at single-cell resolution. Karolinska Institutet. 2025. DOI: 10.69622/29064209.v1

Fig. S 1: *QUADAS-2 assessment of risk of bias and applicability concerns across 38 studies. High risk of bias was most frequent in-patient selection (21%, 8/38), while index test showed minimal bias (0% high risk). Applicability concerns were prominent in-patient selection (63%, 24/38 high concern) but negligible for index/reference standards (0% high concern). Unclear/Medium ratings were common in reference standard (34%, 13/38) and flow/timing (8%, 3/38), reflecting reporting limitations.*

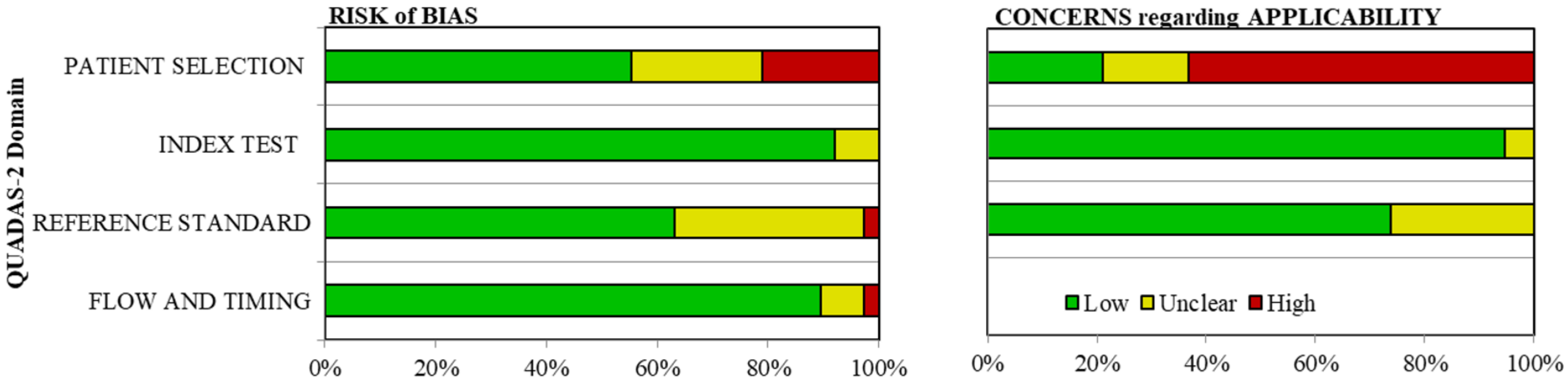

Table S 4: Multimodality imaging protocols and technical parameters for adiposity quantification across studies (1990–2025)

Study (Author, Year)	Modality	Pulse Sequence/Imaging Technique	Technical Innovation	Key Parameters	Field Strength (T)	Scanner Manufacturer and Model
Schneider et al. (2023), Germany	MRI	T1-weighted Gradient Echo	T1-weighted Gradient Echo for visceral fat	Slice Thickness: 10 mm; Voxel Size: 73.2 mm³; Interslice Gap: 0.5 mm; FOV: 530 × 530 × 190 mm; TE: 2.3/4.6 ms (opposed/in-phase); TR: 76 ms; Matrix: 216 × 177; Flip Angle: 70°	1.5	Philips Achieva XR
Hexsel et al. (2022), BRAZIL	MRI	Axial T2-Weighted	Axial T2-weighted pelvic fat mapping	NA	1.5	GE Healthcare SignaTM Explorer SV 25.0
Froelich et al. (2020), Germany	MRI	Axial mDixon and T1-Weighted	Whole-body mDixon for fat fraction quantification	Slice Thickness: 10 mm; Voxel Size: 14 mm³; Interslice Gap: 10 mm; FOV: 520 × 400 × 190 mm; TE: 1.45 ms (mDixon)/7.6 ms (T1); TR: 4.0 ms (mDixon)/672 ms (T1); Matrix: 208 × 161/208 × 197; Flip Angle: 10°/90°	3	NA
Klingensmith et al. (2019), USA	MRI	T1-weighted Volumetric Gradient Echo (Dixon)	Volumetric breath-hold Dixon for thoracic fat	Slice Thickness: 2.5 mm (20% gap); Voxel Size: 5.9 mm³; FOV: 450 mm; TE: 1.23/3.97 ms (out/in-phase); TR: 3.97 ms; Matrix: 320 × 260; Flip Angle: 9°	3	Siemens Skyra System (Munich, Germany)
Petridou et al. (2017), England	MRI	Dynamic Contrast-Enhanced (T1 FSE, STIR, DWI)	Dynamic contrast-enhanced breast fat analysis	Slice Thickness: 1.8 mm; Voxel Size: 5.9 mm³; FOV: 432 × 432 mm; TE: 1.19/2.32 ms; TR: 5.57 ms; Matrix: 232 × 232; Flip Angle: 10°	NA	Discovery MR750w Wide-Bore 3T (GE Healthcare)
Karampatos et al. (2016), Canada	MRI	Axial T1-weighted Fast Spin Echo (FSE)	Lower-leg T1-weighted FSE for muscle-fat segmentation	Slice Thickness (mm) = 2; Interslice Gap (mm) = 0; Field of View (mm)= 160; Echo Time (ms)= 22.9, Repetition Time (ms) = 600; Acquisition Matrix= 320 × 320; Flip Angle (degrees) = 90°	1	GE OrthOne (GE Healthcare)
Silver et al. (2013), USA	MRI	Multigradient-echo (Multiple Fast Field Echo)	Multigradient-echo whole-body fat quantification	Slice Thickness (mm) = 8; Voxel Size (mm³) =32; Interslice Gap (mm) = 0; Field of View (mm)= 500 × 390; Echo Time (ms)= 1.34, 2.87, and 4.40; Repetition Time (ms) =75; Acquisition Matrix=252 × 195; Flip Angle (degrees) = 20°	3	Philips Achieva 3T (Philips Healthcare)

Schautz et al. (2011), Germany	MRI	T1-weighted gradient echo sequence	Dual-scanner protocol for limb fat analysis	Slice Thickness (mm) = 10 mm (Vision); 8 mm (Avanto); Echo Time (ms)= 15 ms (Magnetom Vision), 4 ms (Siemens Avanto); Repetition Time (ms) = 575 ms (Magnetom Vision), 157 ms (Siemens Avanto)	1.5	Magnetom Vision and Avanto (Siemens, Erlangen, Germany)
Ross et al. (2002), Canada	MRI/ CT	T1-weighted for MRI; Standard CT imaging for midthigh muscle attenuation	Cross-modality comparison (MRI vs. CT)	Slice Thickness (mm) = 10 mm (CT); 1 cm (MRI); Interslice Gap (mm) = 40; Fat Attenuation Threshold (HU) =−190 to −30 HU (CT)	1.5	General Electric MRI and CT systems
Gronemeyer et al. (2000), USA	MRI	T1-weighted spin-echo sequence	Early whole-abdomen T1-weighted spin-echo	Slice Thickness (mm) = 10; Interslice Gap (mm) = 38; Field of View (mm)= 500; Echo Time (ms)= 15; Repetition Time (ms) =700; Flip Angle (degrees) = 90°	1.5	Magnetom SP63A (Siemens Medical Systems, Iselin, NJ)
Busetto et al. (2000), ITALY	MRI	Whole-body spin-echo MRI	Whole-body spin-echo for regional adiposity	Slice Thickness (mm) = 10; Voxel Size (mm^3) =40; Interslice Gap (mm) = 50; Field of View (mm)= 500; Echo Time (ms)= 20; Repetition Time (ms) =500; Acquisition Matrix=256 × 256	NA	Philips Gyroscan, Philips Medical Systems
Thomas et al. (1998), England	MRI	Rapid T1-weighted spin-echo	Rapid T1-weighted whole-body fat imaging	Slice Thickness (mm) = 10; Voxel Size (mm^3) =54.76; Interslice Gap (mm) =30 (arms, legs); 10 (torso); Field of View (mm)= 600; Echo Time (ms)= 14; Repetition Time (ms) =36; Acquisition Matrix=256 × 256; Flip Angle (degrees) = 120°	1.0	Picker 1.0T HPQ System
Sohlstrom et al. (1993), Sweden	MRI	Saturation recovery sequence (T1-weighted imaging)	Low-field T1-weighted whole-body fat analysis	Slice Thickness (mm) = 20; Echo Time (ms)= 30; Repetition Time (ms) =250	0.02	Acut Scan 110 (Instrumentarium, Helsinki)
Ross et al. (1993), Canada	MRI	T1-weighted spin-echo sequences	Early whole-body T1 spin-echo for fat quantification	Slice Thickness (mm) = 10; Voxel Size (mm^3) =145.16; Interslice Gap (mm) =50; Field of View (mm)= 500; Echo Time (ms)= 20; Acquisition Matrix=500	1.5	Siemens 1.5-T whole-body scanner
Fowler et al. (1991), Scotland	MRI	Inversion Recovery (IR) sequence	Inversion recovery for anatomical landmark fat mapping	Slice Thickness (mm) = 12; Echo Time (ms)= 170; Repetition Time (ms) =370	0.08	University of Aberdeen's 3.4

						MHz (0.08 T) NMR proton imager
Armao et al. (2006), USA	MRI	T1-weighted spoiled gradient-echo (water-saturation and non-water-saturation sequences)	Water-saturation sequences for abdominal fat	Slice Thickness (mm) = 8; Voxel Size (mm^3) =32.5; Interslice Gap (mm) =2; Field of View (mm)= 400; Echo Time (ms)= 1.61 (water-saturation sequence), 4.4 (non-water-saturation sequence); Repetition Time (ms) =285; Acquisition Matrix=154 × 256; Flip Angle (degrees) = 90°	1.5	Siemens Sonata Medical Solutions, Malvern, PA, USA
Waduud et al. (2017), Scotland	MRI/ CT	True fast imaging with steady-state free precession (MRI)	True fast imaging for L3 vertebra fat	Slice Thickness (mm) = 4; Field of View (mm)= 350 × 240; Echo Time (ms)=1.71; Repetition Time (ms) =4.01; Acquisition Matrix=256 × 176; Flip Angle (degrees) = 60°; Fat Attenuation Threshold (HU) =-190 to -30 HU	1.5	NA
Valentinitsch et al. (2013), USA	MRI	Six-echo spoiled gradient echo (SPGR) sequence with chemical shift-based water-fat separation	Six-echo SPGR for calf/thigh fat-water separation	Slice Thickness (mm) = 4 mm (calf); 5 mm (thigh); Field of View (mm)= 180 mm (calf); 200 mm (thigh); Echo Time (ms)= 1.4 ms (calf); 1.3 ms (thigh); Repetition Time (ms) =12 ms (calf); 10.8 ms (thigh); Acquisition Matrix=180 × 180; Flip Angle (degrees) = 3°	3	Signa HDx, General Electric Healthcare
Kway et al. (2023), England	MRI	Two-point Dixon	Two-point Dixon for automated segmentation	Slice Thickness (mm) =3; Voxel Size (mm^3) =6.75; Echo Time (ms)= 1.23; Repetition Time (ms) =3.86; Flip Angle (degrees) = 9°	3	Siemens 3T Skyra Scanner (S-PRESTO) and Siemens 3T Tim Trio Scanner
Haueise et al. (2023), Germany	MRI	T1-weighted 3D VIBE Dixon	3D VIBE Dixon for trunk fat quantification	Slice Thickness (mm) = 3; Voxel Size (mm^3) =5.88; Echo Time (ms)= 1.23 ms and 2.46 ms; Repetition Time (ms) =4.36	3	Siemens MAGNETOM Skyra
Linder et al. (2019), Germany	MRI	Two-point Dixon fat-sensitive transverse imaging	Two-point Dixon for abdominal fat	Slice Thickness (mm) = 10; Interslice Gap (mm) =0.5	1.5	Achieva d Stream, Philips Healthcare, Best, Netherlands

Maislin et al. (2012), USA	MRI	Gradient Echo Pulse Sequence	Gradient echo for abdominal compartment fat	Slice Thickness (mm) = 10; Interslice Gap (mm) =0	1.5	Siemens Avanto, Germany
Schwenzer et al. (2010), Germany	MRI	Axial T1-weighted fast spin-echo	Whole-body T1 FSE for fat segmentation	Slice Thickness (mm) =10; Interslice Gap (mm) =10; Field of View (mm)= 450–530; Echo Time (ms)=12; Repetition Time (ms) =490; Acquisition Matrix=256 × 178; Flip Angle (degrees) = 3°	1.5	Magnetom Sonata (Siemens Healthcare, Erlangen, Germany)
Positano et al. (2004), ITALY	MRI	Axial T1-weighted fast gradient echo (FIESTA)	FIESTA sequence for abdominal fat	Field of View (mm)= 360–480; Echo Time (ms)= 4.2; Repetition Time (ms) =120; Acquisition Matrix=256 × 256; Flip Angle (degrees) = 90°	1.5	GE Signa Horizon LX System
Bea et al. (2022), USA	DXA	DXA Whole-Body Scan	AI-driven regional fat% analysis	Field of View (mm)= 500 × 400 mm; Radiation Dose (CT only, mSv) = Minimal (DXA specific dose <1 mSv)	NA	DXA: Hologic QDR2000/4500W
Flehr et al. (2022), SWEDEN	HR-pQCT	HR-pQCT with voxel peeling for radiodensity analysis	Voxel peeling for bone marrow radiodensity	Slice Thickness: 82 μm; Voxel Size: 0.00055 mm³; Fat Attenuation: −80 to +2000 HU; Radiation Dose: <0.1 mSv	NA	XtremeCT (Scanco Medical AG)
Jayawarde et al. (2020), USA	CT	Electron Beam CT (EBCT) and Multidetector CT (MDCT) with Prospective ECG-Triggering	EBCT/MDCT for epicardial fat	Slice Thickness (mm) =1.5 mm, 2.5 mm, or 3.0 mm (depending on scanner); Fat Attenuation Threshold (HU) =−190 to −30 HU	NA	EBCT: C150XP or C300 Electron Beam CT Scanner MDCT: GE Helical Scanner or Siemens Multi-Slice Scanner
Priya and Sudha (2019), India	CT	Non-contrast CT of the cardiac region	Non-contrast cardiac CT for mediastinal fat	Acquisition Matrix=512 × 512; Fat Attenuation Threshold (HU) =−100 HU	NA	NA
Wang et al. (2017), USA	CT	Abdominal CT with a GE "Standard" image reconstruction kernel	Standardized HU thresholds for VAT/SAT	Slice Thickness (mm) =5; Acquisition Matrix=12 × 512; Fat Attenuation Threshold (HU) =-140 to -40 HU	NA	GE LightSpeed VCT (64-detector) or GE Discovery 600 (16-detector)

Middleton et al. (2017), USA	MRS	3D Spoiled Gradient-Recalled Echo (SGRE), Chemical Shift-Encoded MRI	3D SGRE for whole-body fat spectroscopy	Slice Thickness (mm) =3.5; Echo Time (ms)= 1.15–2.3; Radiation Dose (CT only, mSv) = 4.1–4.3; Acquisition Matrix=256 × 160; Flip Angle (degrees) = 10°	3	GE Signa EXCITE HDxt (GE Medical Systems, Milwaukee, WI)
Piché et al. (2008), Canada	CT	Abdominal and midthigh CT scans	Abdominal/midthigh CT for fat attenuation	Slice Thickness (mm) =10; Fat Attenuation Threshold (HU) =−190 to −30 HU	NA	GE High-Speed Advantage CT Scanner (General Electric Medical Systems, Milwaukee, WI)
Schapira et al. (1994), USA	CT	Single axial slice at the L4 vertebral level	Single-slice L4 CT for abdominal fat	Slice Thickness (mm) =8; Fat Attenuation Threshold (HU) =−30 to −200 HU	NA	Siemens HI-Q Scanner (Siemens, Germany)
Davidson et al. (2020), South Africa	DXA	Single axial slice at the L4–L5 vertebral level, sequential scanning mode (CT)	DXA/CT fusion for android fat analysis	Slice Thickness (mm) =5; Voxel Size (mm³) = 3.05; Acquisition Matrix=512 × 512	NA	GE 16-Slice Lightspeed CT scanner
Bi et al. (2015), USA	DXA	Lunar iDXA whole-body scanning	Whole-body DXA for android fat%	NA	NA	Lunar iDXA whole-body scanner (GE Healthcare, Madison, WI, USA)
Bredella et al. (2010), USA	DXA	Single-slice CT imaging	Dual-energy CT for abdominal/thigh fat	Slice Thickness (mm) =3.5; Field of View (mm)= 480; Radiation Dose (CT only, mSv) = 80 kV for abdomen, 120 kV for thigh; 70 mA for abdomen, 170 mA for thigh; Fat Attenuation Threshold (HU) =−50 to −250 HU	NA	
Lee et al. (2018), USA	CT	Non-contrast abdominal CT	Low-dose protocol for abdominal fat	Slice Thickness (mm) =1.25; Radiation Dose (CT only, mSv) = Variable low-dose protocol, 120 kVp with modulated mA; Fat Attenuation Threshold (HU) = −274 to −49 HU	NA	GE Healthcare multi-detector-row CT scanners

Mourtzakis et al. (2008), CANADA	CT	Standard diagnostic CT imaging	L3 CT for lumbar fat quantification	Slice Thickness (mm) =5; Voxel Size (mm³) =125; Field of View (mm)= 260	NA	NA
Kim et al. (2019), SOUTH KOREA	CT	Multi-detector row CT (MDCT)	MDCT for umbilical/L4 fat analysis	Slice Thickness (mm) =10; Interslice Gap (mm) =50; Voxel Size (mm³) =40; Field of View (mm)= 500	NA	NA

Footnote: *Abbreviations: MRI (Magnetic Resonance Imaging), CT (Computed Tomography), DXA (Dual-Energy X-ray Absorptiometry), HR-pQCT (High-Resolution peripheral Quantitative Computed Tomography), MRS (Magnetic Resonance Spectroscopy), Imaging sequences: SPGR (Spoiled Gradient-Recalled Echo), FIESTA (Fast Imaging Employing Steady-State Acquisition), VIBE (Volumetric Interpolated Breath-hold Examination), mDixon (modified Dixon technique), EBCT (Electron Beam CT), MDCT (Multidetector CT), SGRE (Spoiled Gradient-Recalled Echo); Parameters: HU (Hounsfield Units, fat attenuation thresholds), ms (milliseconds), mm (millimeters), mSv (millisieverts, radiation dose), voxel size (mm³); NA indicates data not reported or unavailable. Scanner manufacturers and models are included for technical reproducibility. Field strength (T) denotes Tesla for MRI systems.*

Fig. S2: Bland-Altman plot comparing automated versus conventional methods for fat volume measurement shows a mean bias of -22.3 cm^3 (95% limits of agreement: -854.94 to 810.34), indicating no systematic measurement bias between methods despite wide variability in individual measurements (range: 0-30000 cm^3).

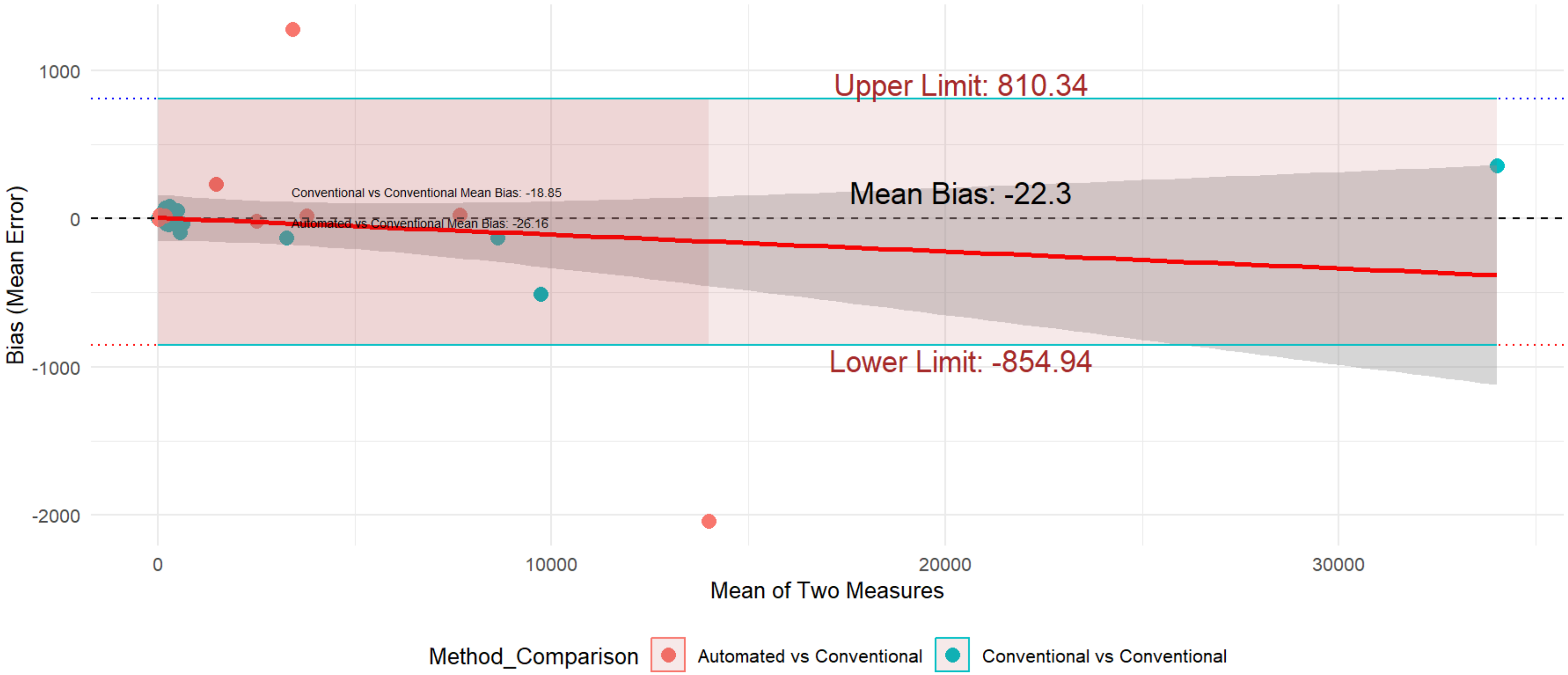

Fig. S 3: Forest plot displays subgroup analysis of total fat volume (TFV) by age categories (Median age >47.1 vs ≤47.1 years), revealing no significant differences between age groups (p=0.854) despite extreme heterogeneity in both subgroups (I²=99.8%). Younger participants showed wider TFV range (11,308-49,150 cm³) compared to older participants (4,897-79,684 cm³).

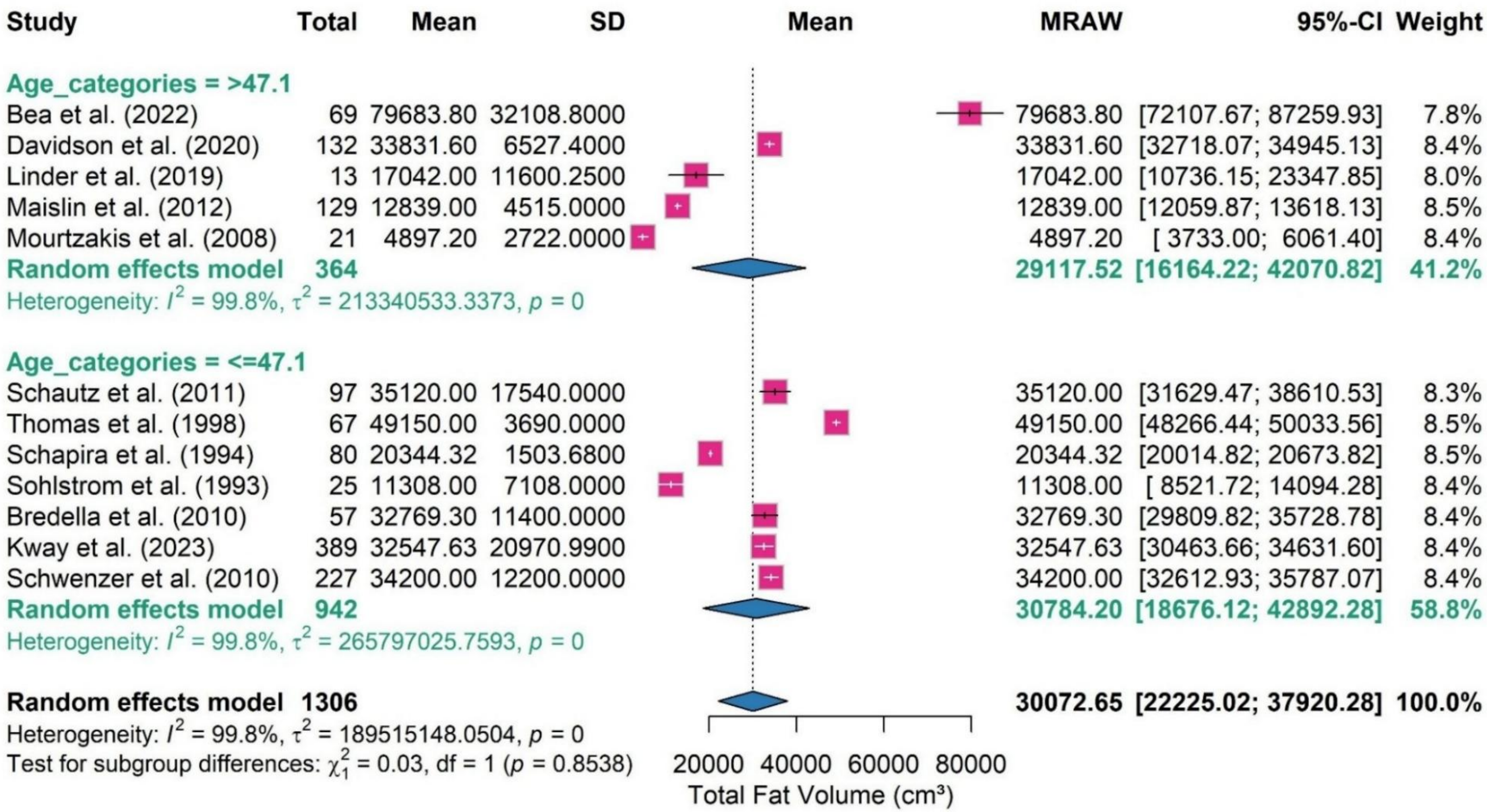

Fig. S 4: Forest plot presents subgroup analysis of total fat volume (TFV) by body region (regional vs whole-body), showing no significant differences between measurement approaches (p=0.939) despite extreme heterogeneity in both subgroups (I²=99.6-99.9%). Regional measurements demonstrated wider TFV range (4,897-149,383 cm³) compared to whole-body assessments (20,344-49,150 cm³).

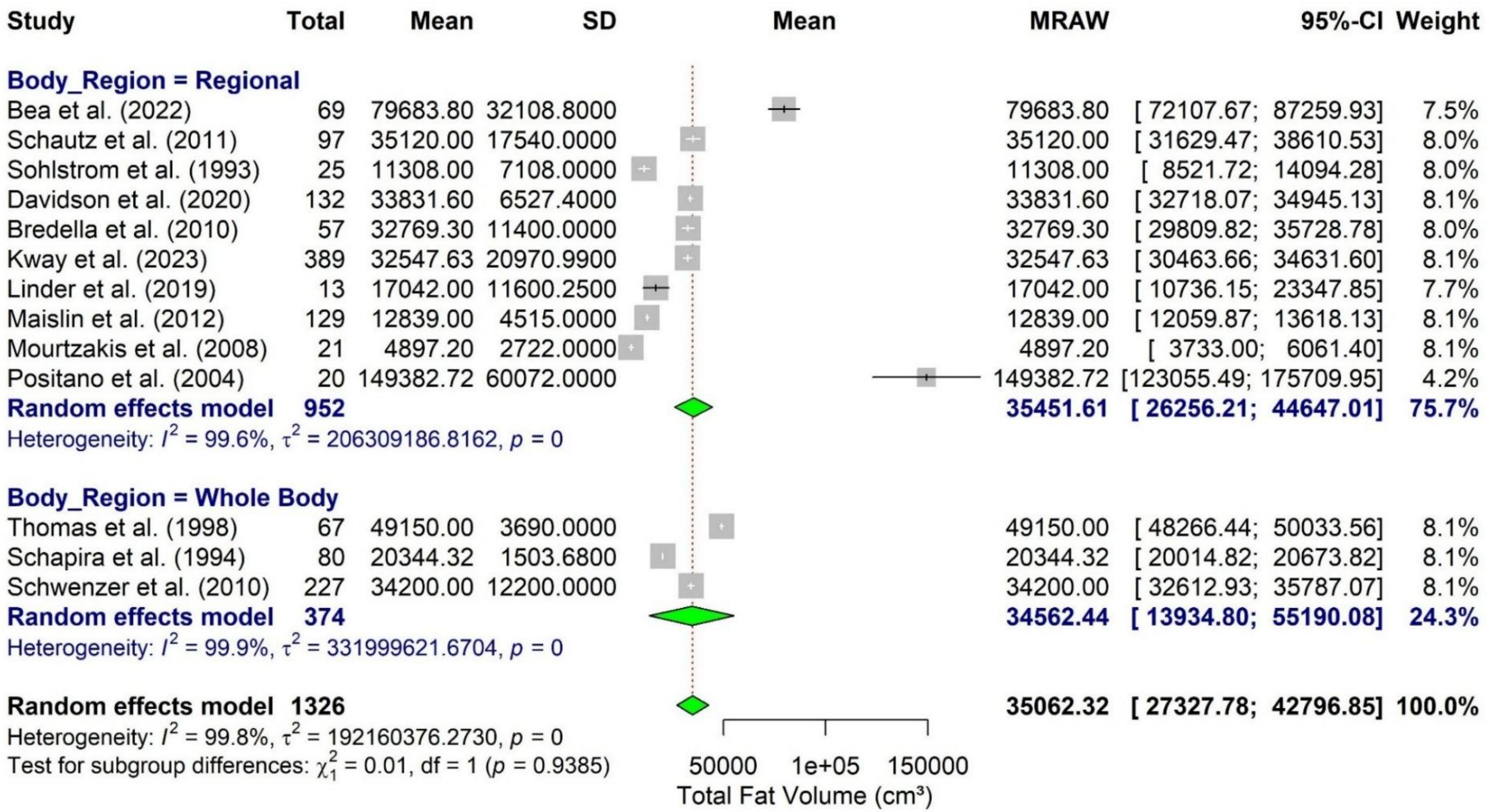

Fig. S 5 : Forest plot displays subgroup analysis of TFV by comorbidity status, revealing no significant differences between groups with and without comorbidities (p=0.756), though comorbidity-positive studies showed greater heterogeneity (I²=99.9% vs 98.5%). The comorbidity group included both the lowest (4,897 cm³) and highest (149,383 cm³) observed TFV values.

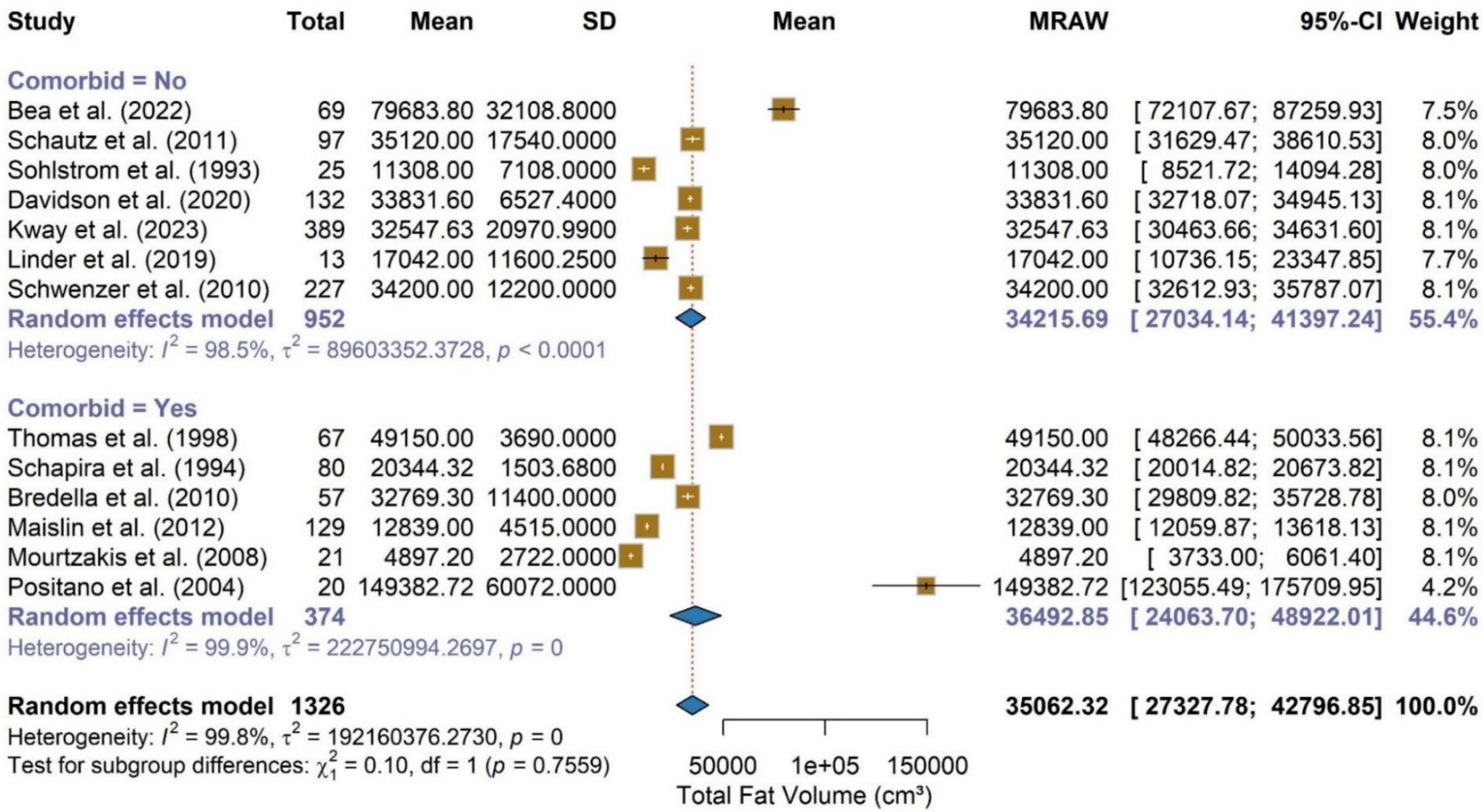

Fig. S 6: Forest plot illustrates subgroup analysis of TFV by imaging modality (DXA/MRI/CT), demonstrating significant differences between modalities (p=0.003) with MRI studies showing the widest TFV range (11,308-149,383 cm³) and highest heterogeneity (I²=99.8%, τ²=398,752,853). DXA measurements were consistently higher (32,769-79,684 cm³) than CT values (4,897-20,344 cm³).

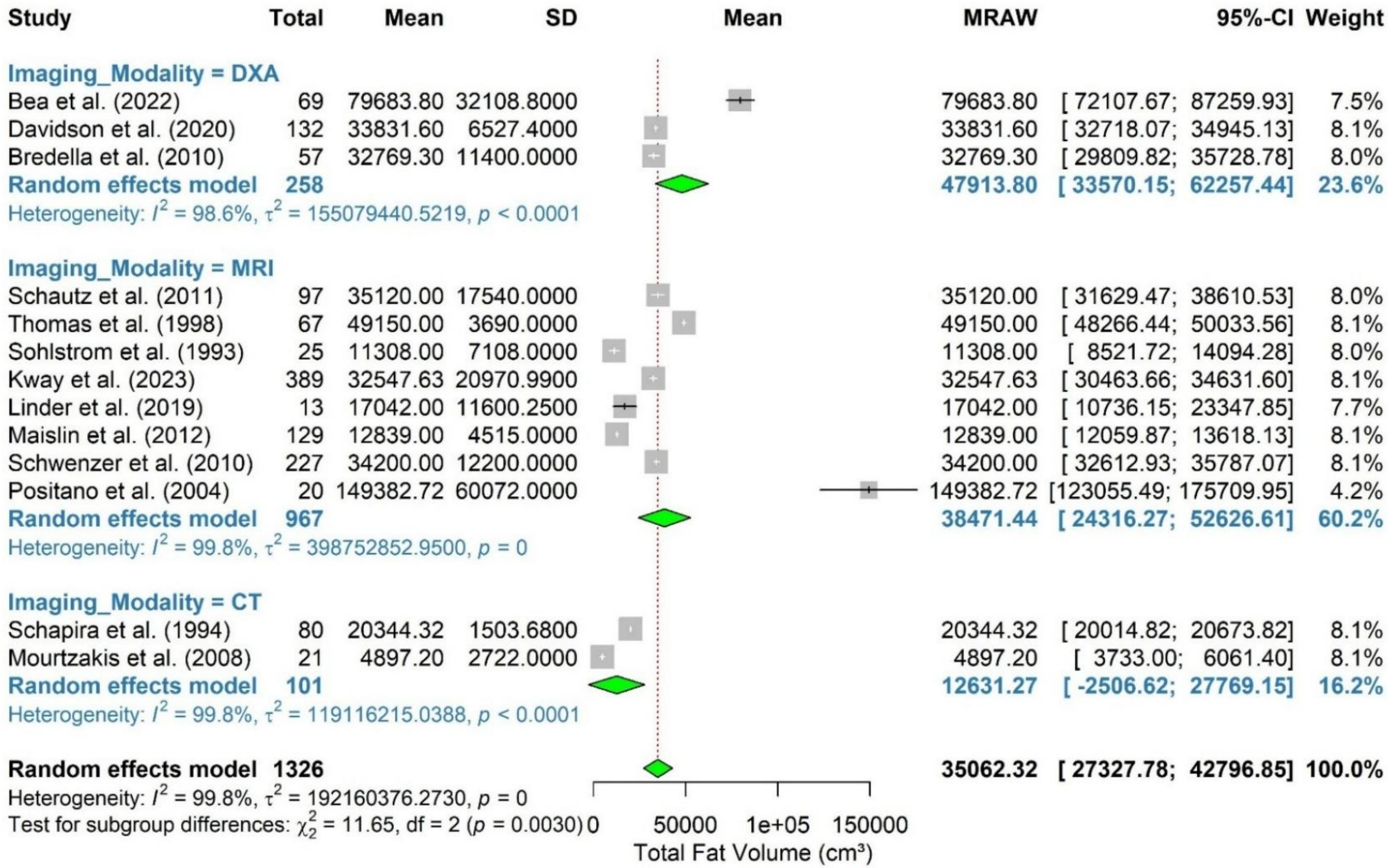

Fig. S 7 : Forest plot of total fat volume (TFV) across 13 studies (n=1326) stratified by obesity status (Obese, Overweight). Extreme heterogeneity was noted overall (I^2=99.8%) and within subgroups (Obese: I^2=99.9%; Overweight: I^2=99.1%). TFV ranged from 4,897 cm³ (Mourtzakis et al., 2008) to 149,383 cm³ (Positano et al., 2004). The pooled estimate was 35,062 cm³ (95% CI: 27,328–42,797), with a prediction interval of 16,042–54,083 cm³. Subgroup differences were significant (p=0.048).

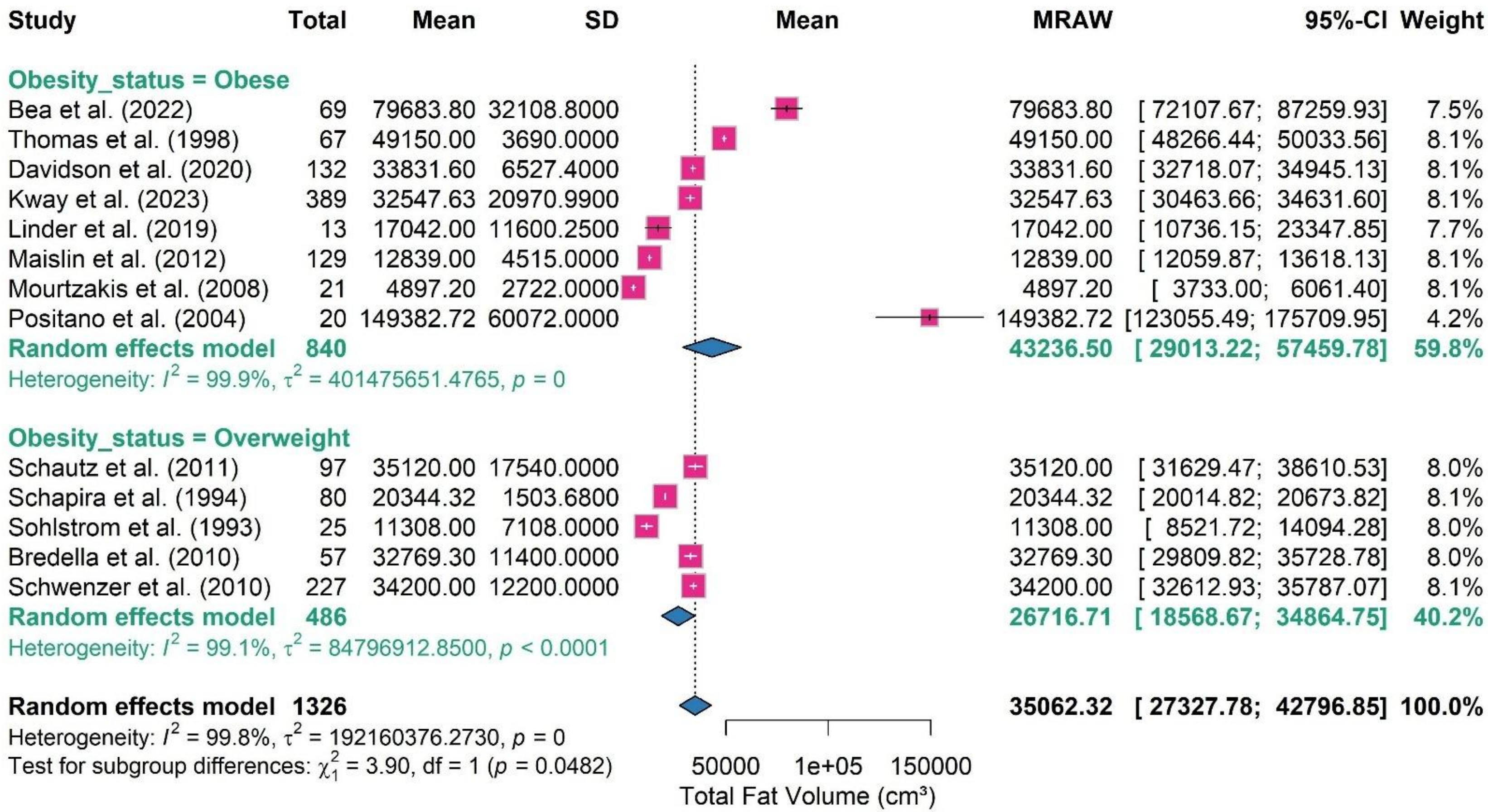

Fig. S 8: Forest plot of total fat area (TFA) across 9 studies (n=952). Extreme heterogeneity was observed (I²=98.9%), with means ranging from 232.79 cm² (Kim et al., 2019) to 643.00 cm² (Ross et al., 1993). The pooled estimate was 411.12 cm² (95% CI: 289.90–532.35), and the prediction interval (94.16–728.09 cm²) underscored high between-study variability.

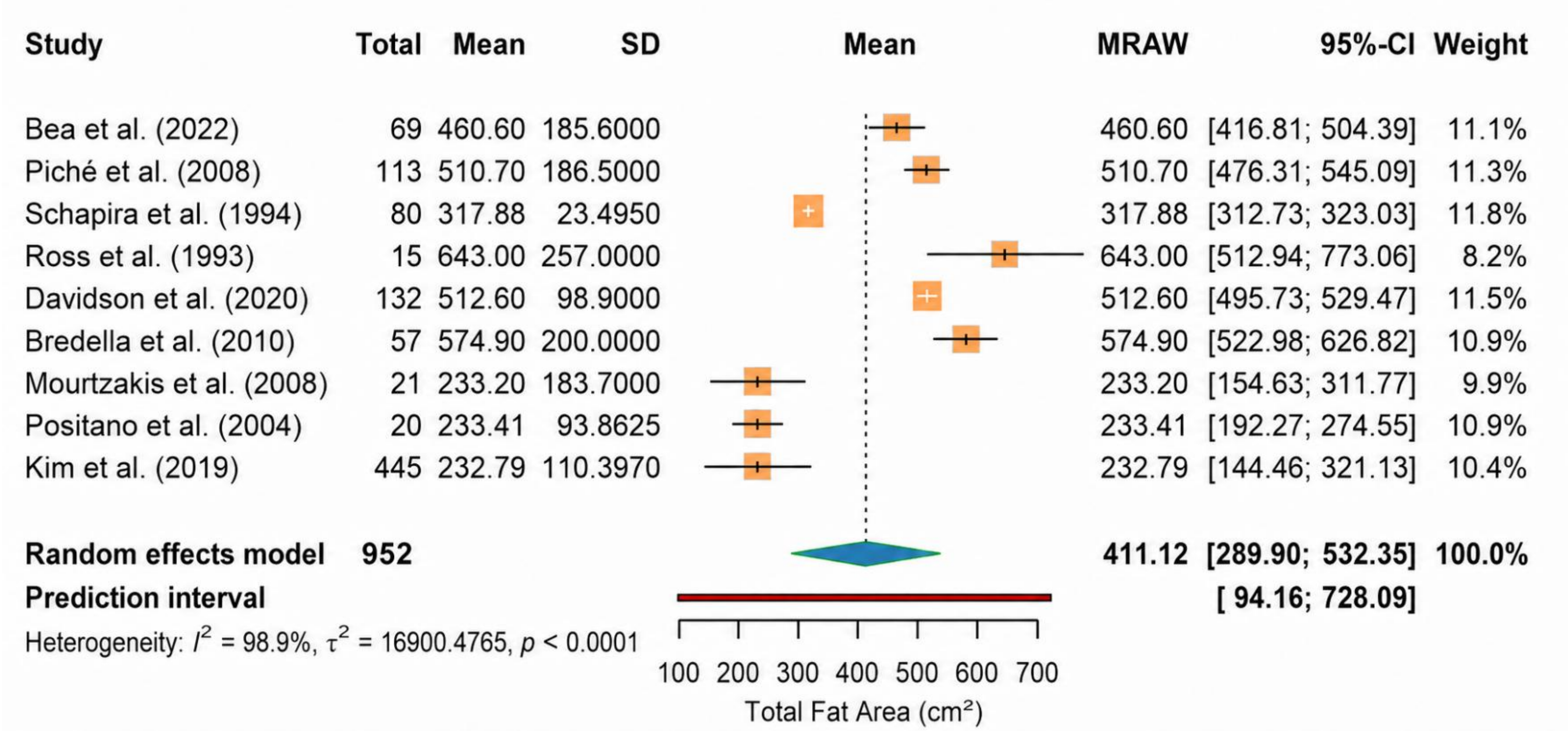

Fig. S 9: Forest plot of total fat area across 8 studies (n=932) stratified by age (Median age ≤47.1 vs. >47.1 years). Extreme heterogeneity was observed overall (I²=99.0%) and within subgroups (>47.1: I²=95.2%; ≤47.1: I²=98.3%). The pooled estimate was 434.25 cm² (95% CI: 338.52–529.97), with older participants showing lower but non-significant differences (subgroup p=0.374).

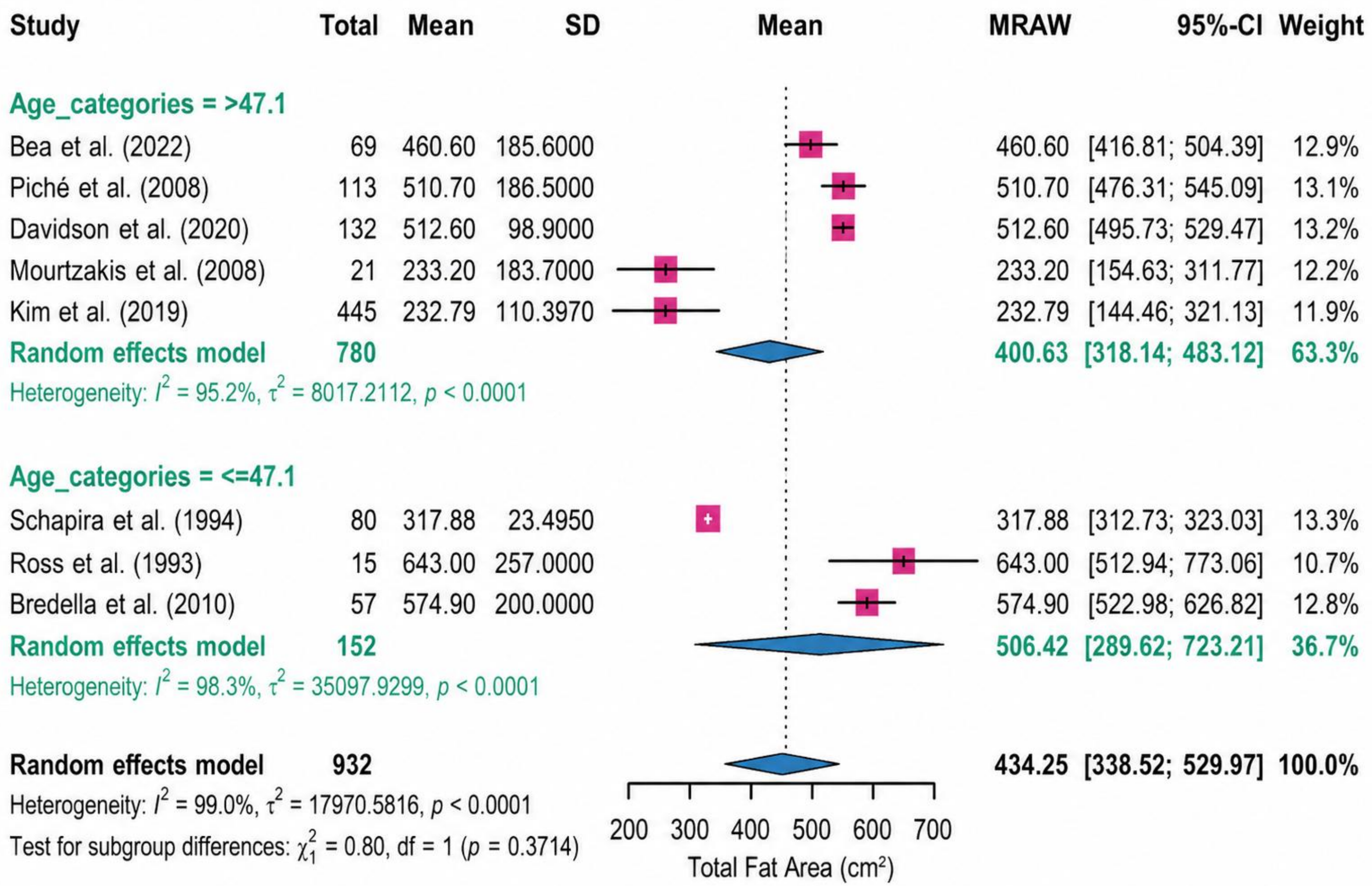

Fig. S 10: Forest plot of TFA across 9 studies (n=952) comparing regional vs. whole-body measurements. Extreme heterogeneity persisted overall (I²=98.9%) and within subgroups (Regional: I²=97.5%; Whole-body: I²=95.8%). The pooled estimate was 411.12 cm² (95% CI: 323.64–498.61), with no significant subgroup differences (p=0.655). Notably, whole-body studies showed wider variability (e.g., 317.88 cm² to 643.00 cm² vs. 232.79–574.90 cm² for regional).

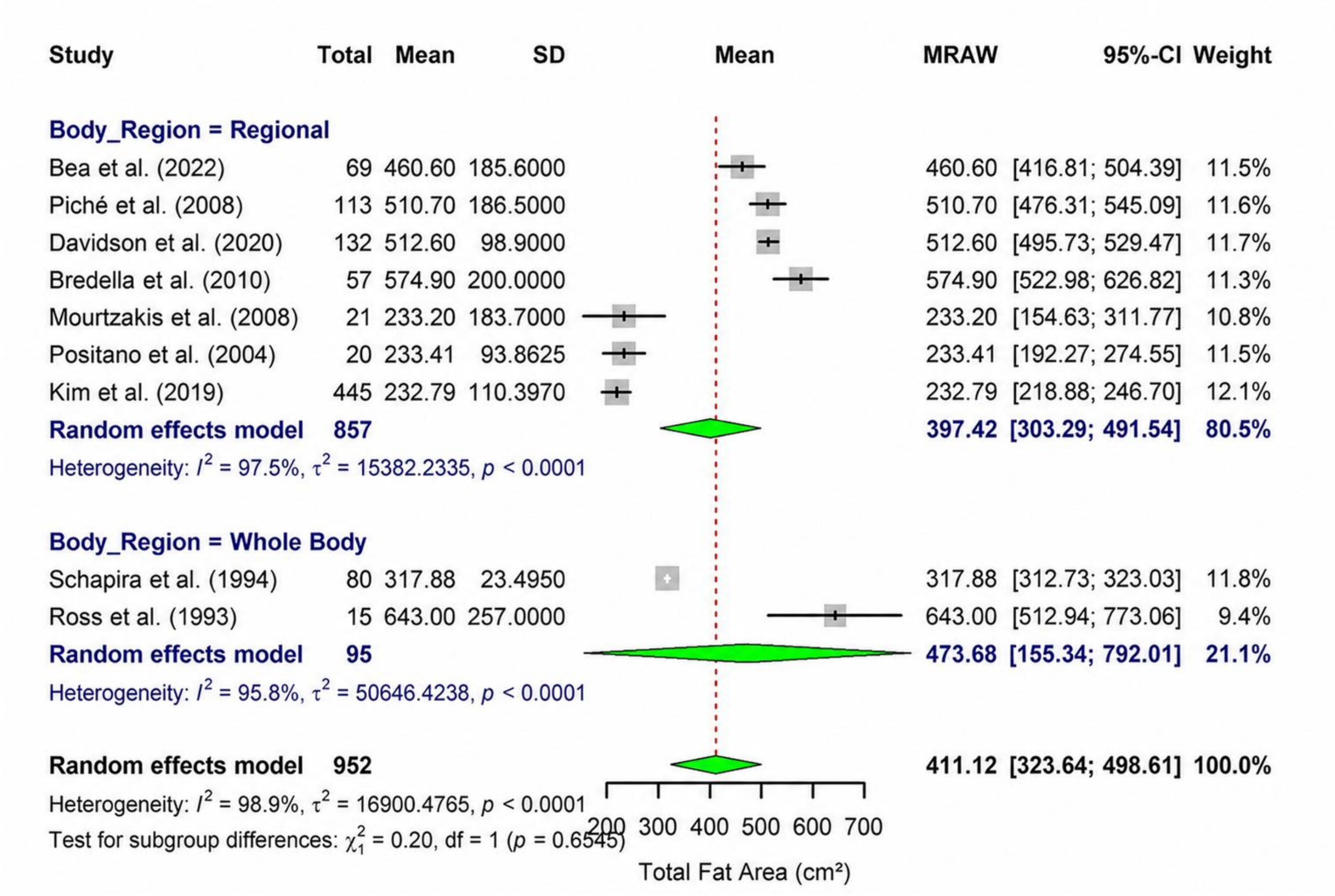

Fig. S 11: Forest plot of total fat area (TFA) across 9 studies (n=741) stratified by comorbidity status. Significant subgroup differences were detected (p=0.0006), with non-comorbid participants showing higher pooled estimates (506.62 cm², 95% CI: 472.73–540.51) and lower heterogeneity (I^2=66.5%) compared to comorbid groups (320.84 cm², 95% CI: 220.68–421.01; I^2=96.6%). Extreme overall heterogeneity was observed (I^2=98.9%).

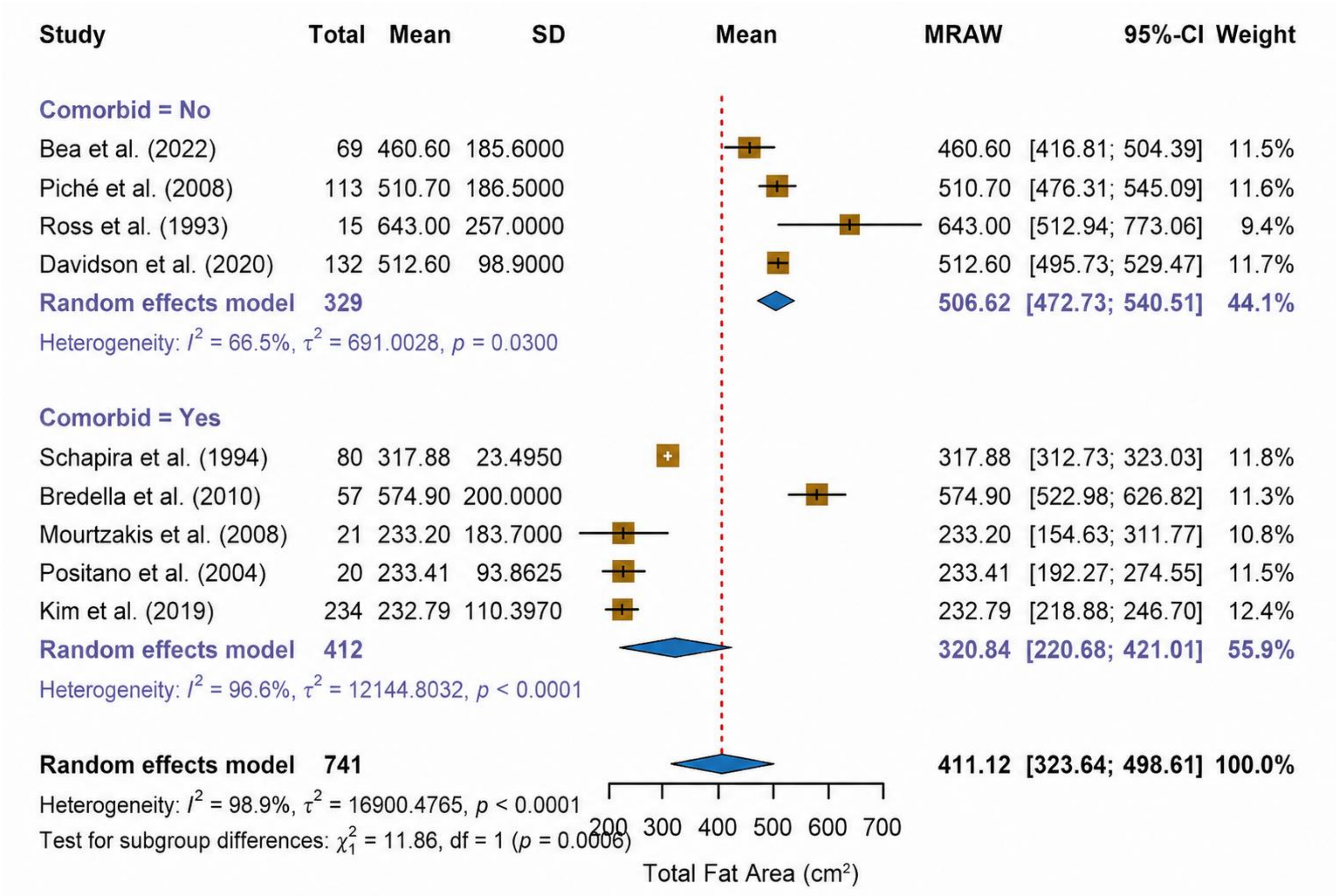

Fig. S 12: Forest plot of TFA across 9 studies (n=952) stratified by imaging modality. Significant subgroup differences were observed (p=0.021), with DXA showing the most consistent measurements (I²=81.7%, pooled estimate=514.07 cm² [95% CI: 464.40-563.74]). Extreme heterogeneity was noted in CT (I²=97.6%) and MRI (I²=97.1%) subgroups, with MRI demonstrating the widest variability (233.41-643.00 cm²). The overall pooled estimate was 411.12 cm² (95% CI: 323.64-498.61) with extreme heterogeneity (I²=98.9%).

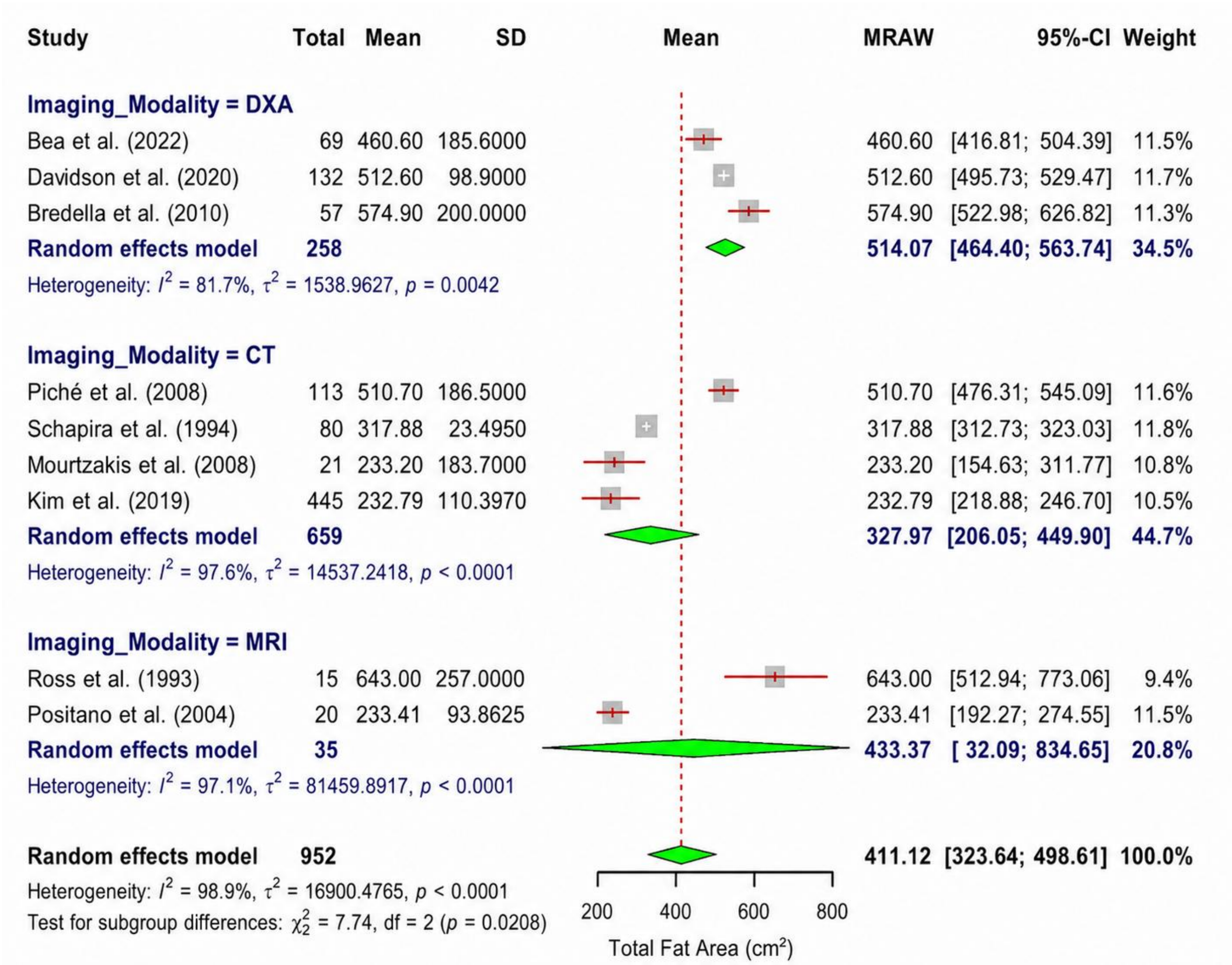

Fig. S 13: Forest plot of TFA across 9 studies comparing obese (n=397) and overweight (n=137) populations. No significant subgroup differences were found (p=0.752), despite extreme heterogeneity in both groups (obese: I²=97.3%; overweight: I²=98.9%). The obese subgroup showed a pooled estimate of 401.27 cm² (95% CI: 302.45-500.09), while overweight studies demonstrated wider variability (317.88-574.90 cm²). The overall estimate was 411.12 cm² (95% CI: 323.64-498.61).

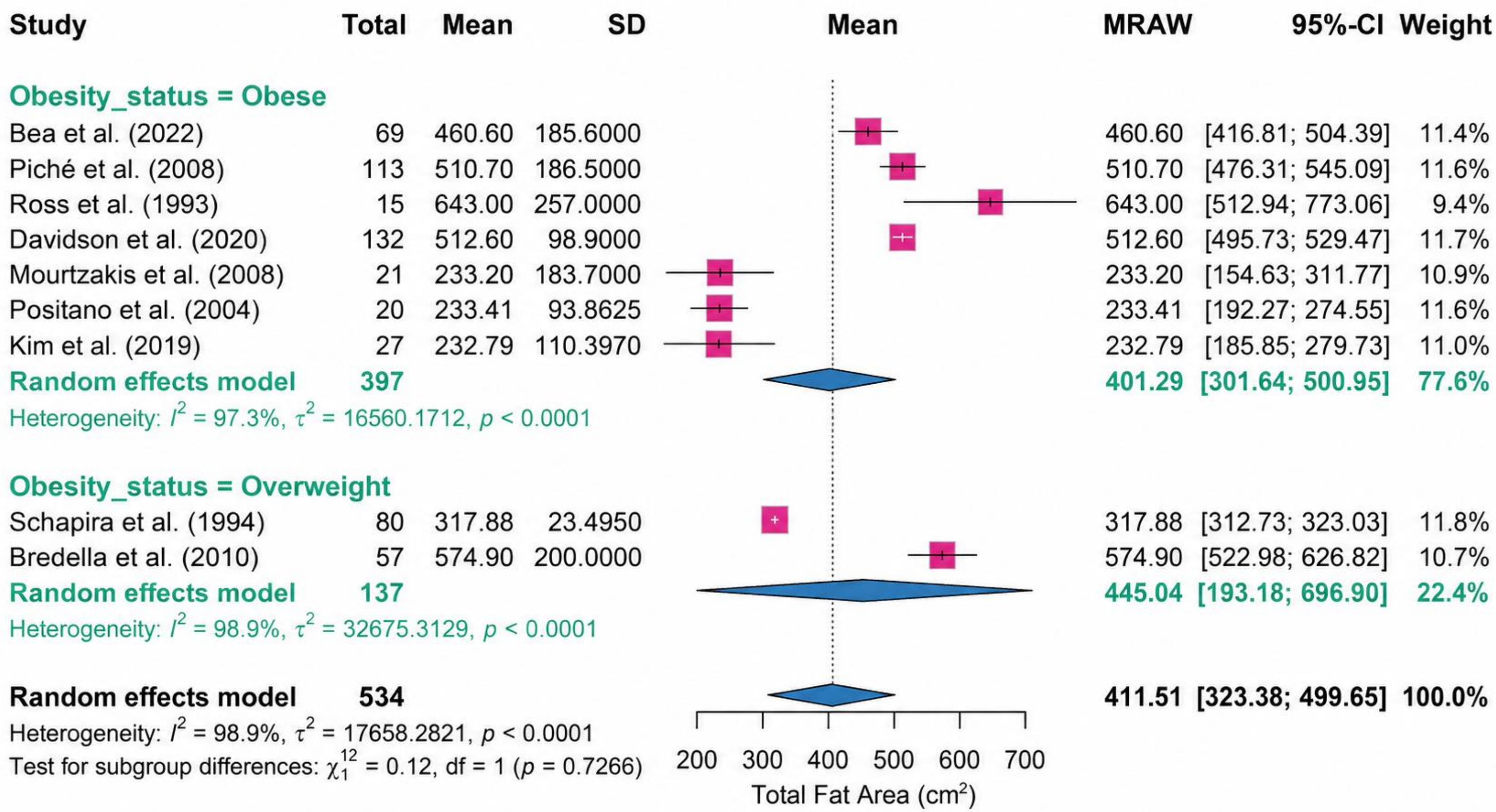

Fig. S 14: Forest plot of TFA across 7 studies (n=854) stratified by waist circumference (median in meter ≤0.95 vs >0.95). No subgroup differences were detected (p=0.944), though both groups showed extreme heterogeneity (≤0.95: I²=99.5%; >0.95: I²=96.2%). The ≤0.95 subgroup had a pooled estimate of 450.05 cm² (95% CI: 320.02-580.09), while the >0.95 subgroup showed paradoxical results (232.79-643.00 cm²). The overall estimate was 442.87 cm² (95% CI: 333.90-551.85).

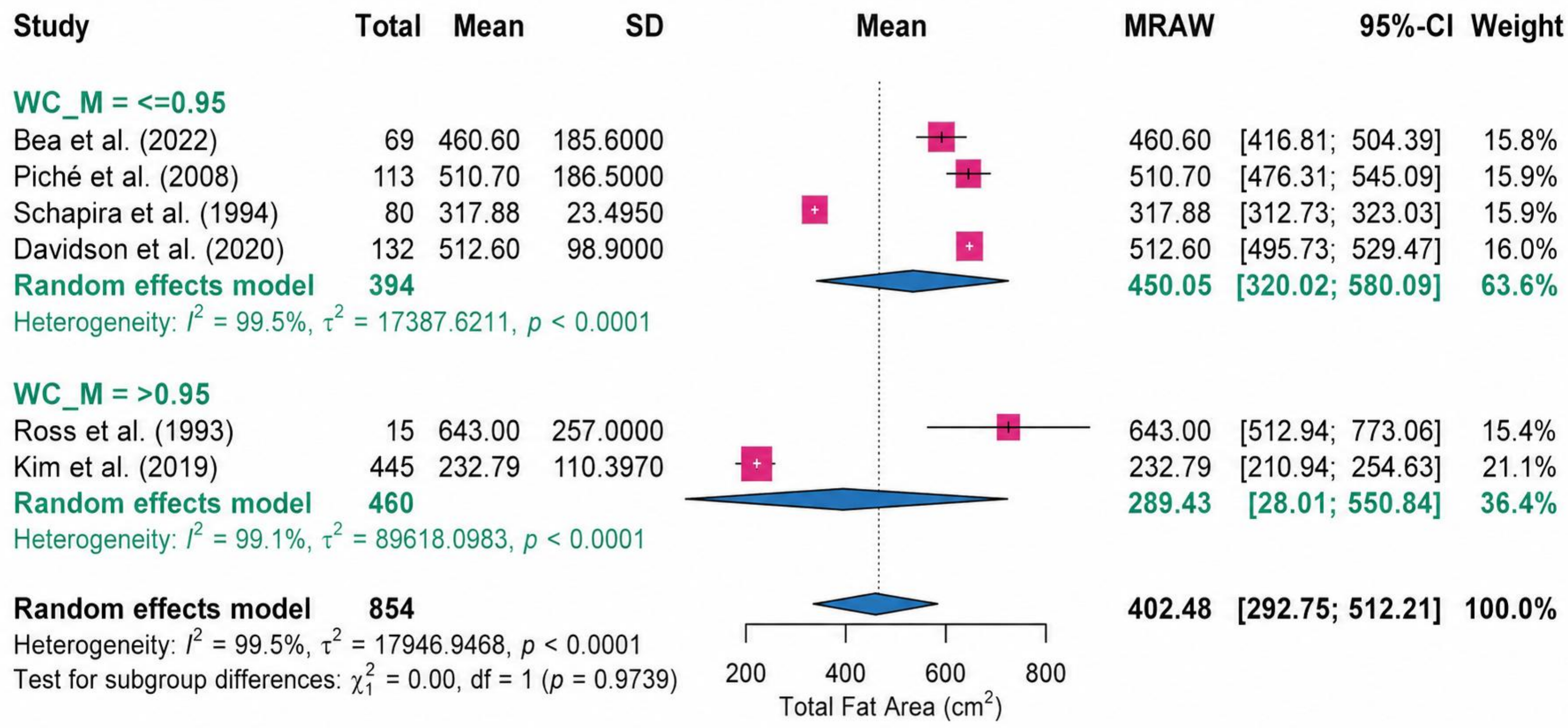

Fig. S 15: Forest plot comparing subcutaneous fat volume stratified by body weight (>75 kg., n=391) vs below the median value (≤74.9, n=308) populations across 8 studies (n=699). Significant subgroup differences were found (p=0.0005), with high weight participants showing higher pooled volumes (34,155.82 cm³, 95% CI: 27,772.25-40,539.39) compared to participants having lower weight (24,129.60-28,200.00 cm³). Extreme heterogeneity was observed in both subgroups ((>75 k.g: I²=98.4%; ≤74.9: I²=99.6%) and overall (I²=99.9%).

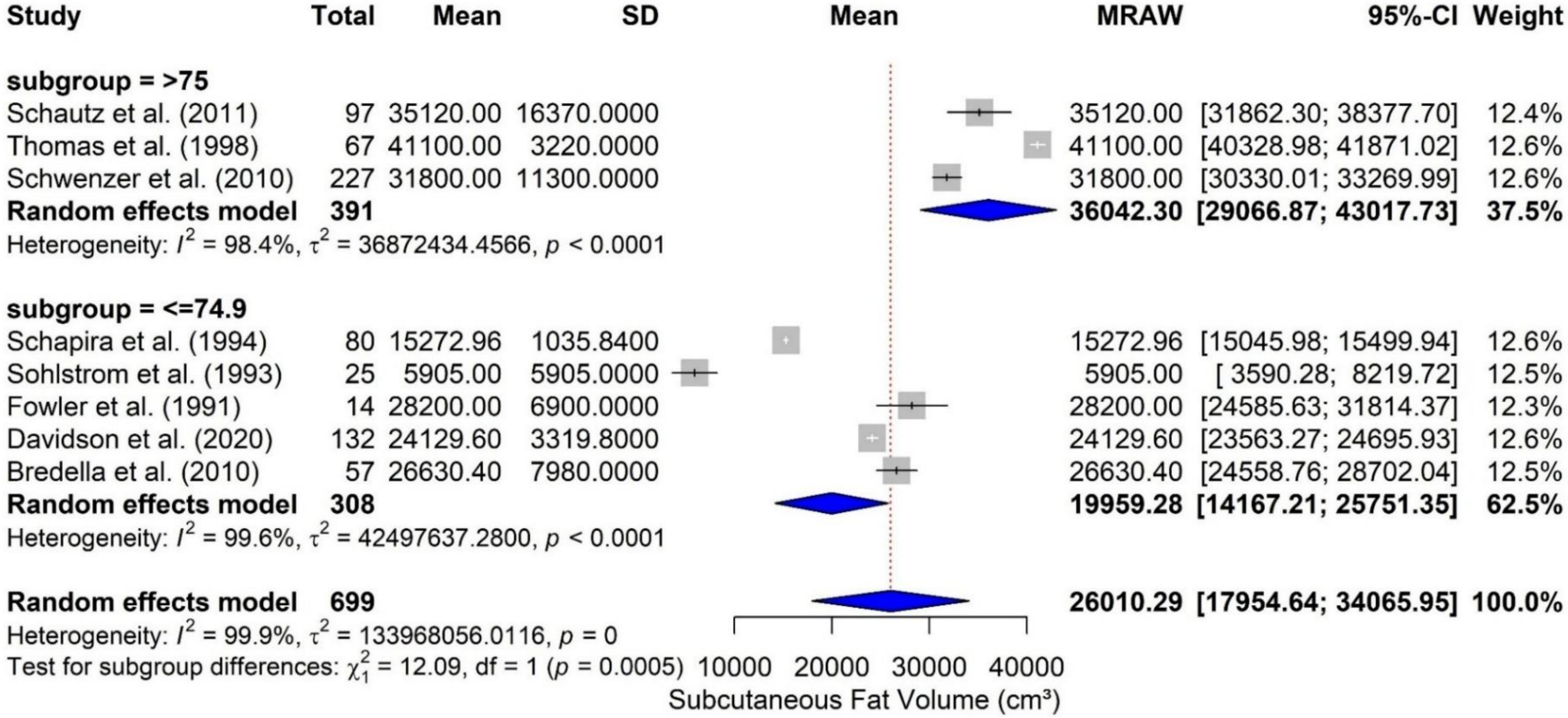

Fig. S 16: Forest plot of 7 studies (n=594) stratified by height (above and below the median value in meter (>1.60 vs ≤1.60). Significant subgroup differences emerged (p=0.0099), with higher-height groups demonstrating greater fat volumes (34,155.82 cm³, 95% CI: 27,772.25-40,539.39) versus lower-height groups (25,173.32 cm³, 95% CI: 22,756.29-27,590.35). Notably, heterogeneity was substantially lower in the ≤1.60 subgroup (I^2=80.8% vs 98.1%).

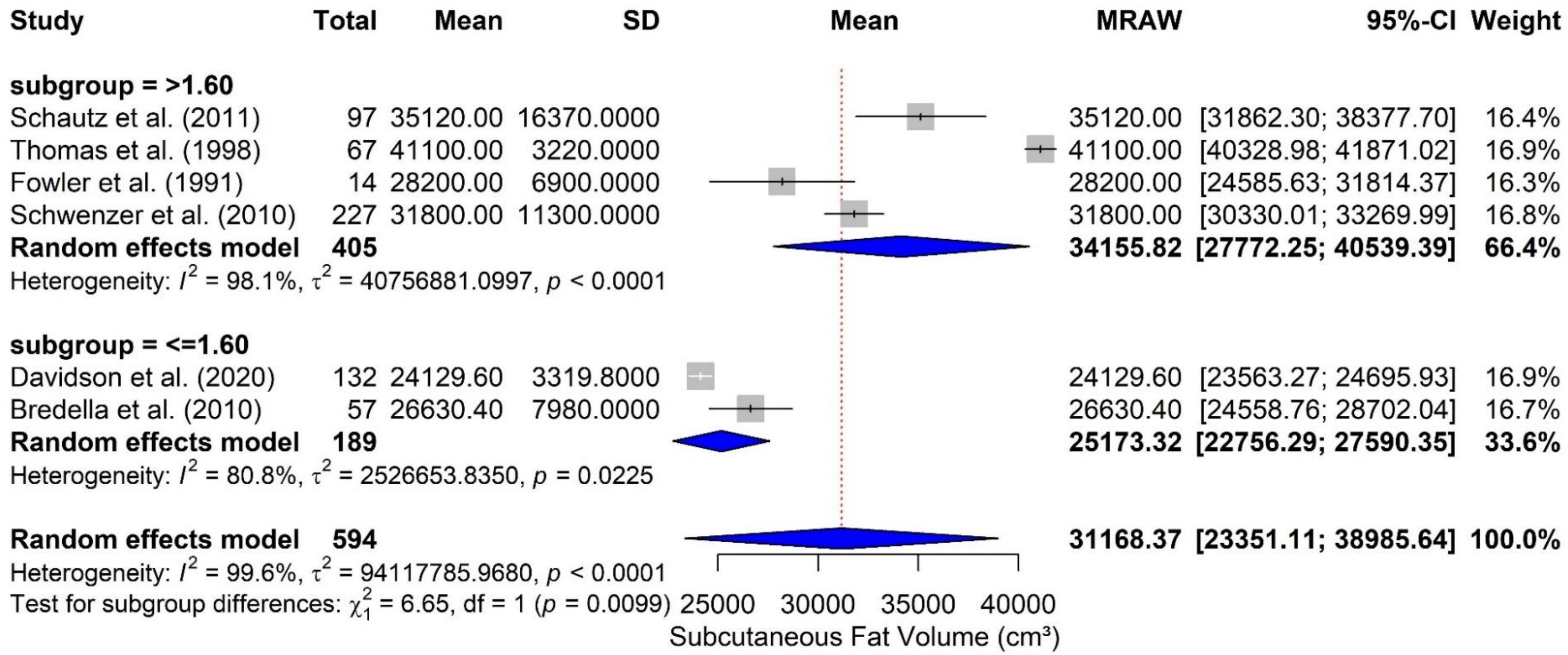

Fig. S 17: Forest plot of 12 studies (n=995) significant differences in subcutaneous fat volume (SFV) comparing MRI (n=705), CT (n=101), and DXA (n=189) measurements. Significant modality effects were found (p=0.0241), with MRI showing the widest range (3,524.50-41,100.00 cm³) and highest heterogeneity (I²=99.9%). DXA measurements demonstrated the lowest heterogeneity (I²=80.8%). The overall estimate was 31,168.37 cm³ (95% CI: 23,351.11-38,985.64) with extreme heterogeneity (I²=99.9%).

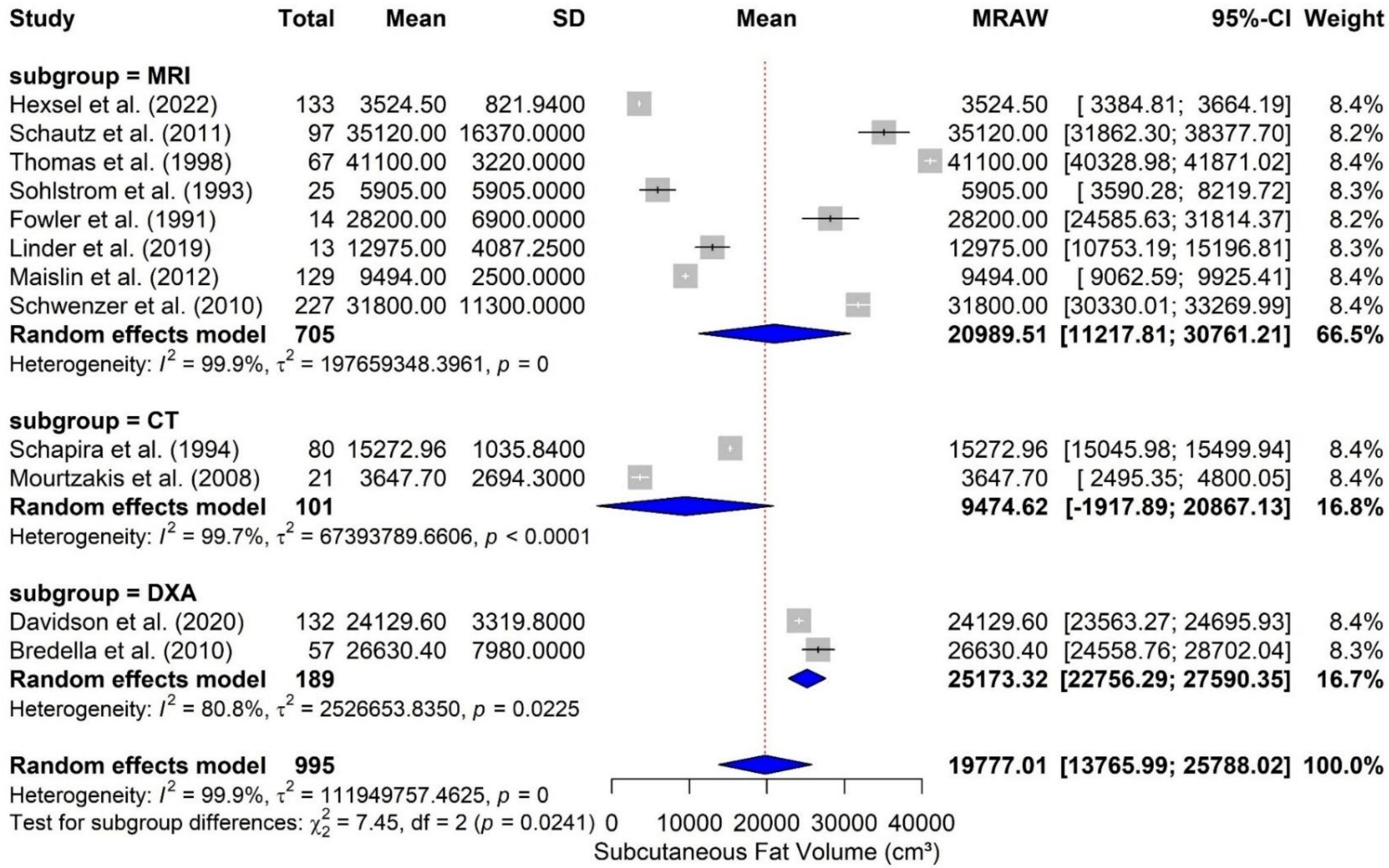

Fig. S 18: Forest plot presents subgroup analysis of subcutaneous fat volume (SFV) by age categories (≤47.1 vs >47.1 years), showing no significant difference between age groups (p=0.090) despite extreme heterogeneity in both subgroups (I²=100% and 99.8% respectively), with younger participants demonstrating wider SFV range (3,524-41,100 cm³) compared to older participants (3,647-24,130 cm³).

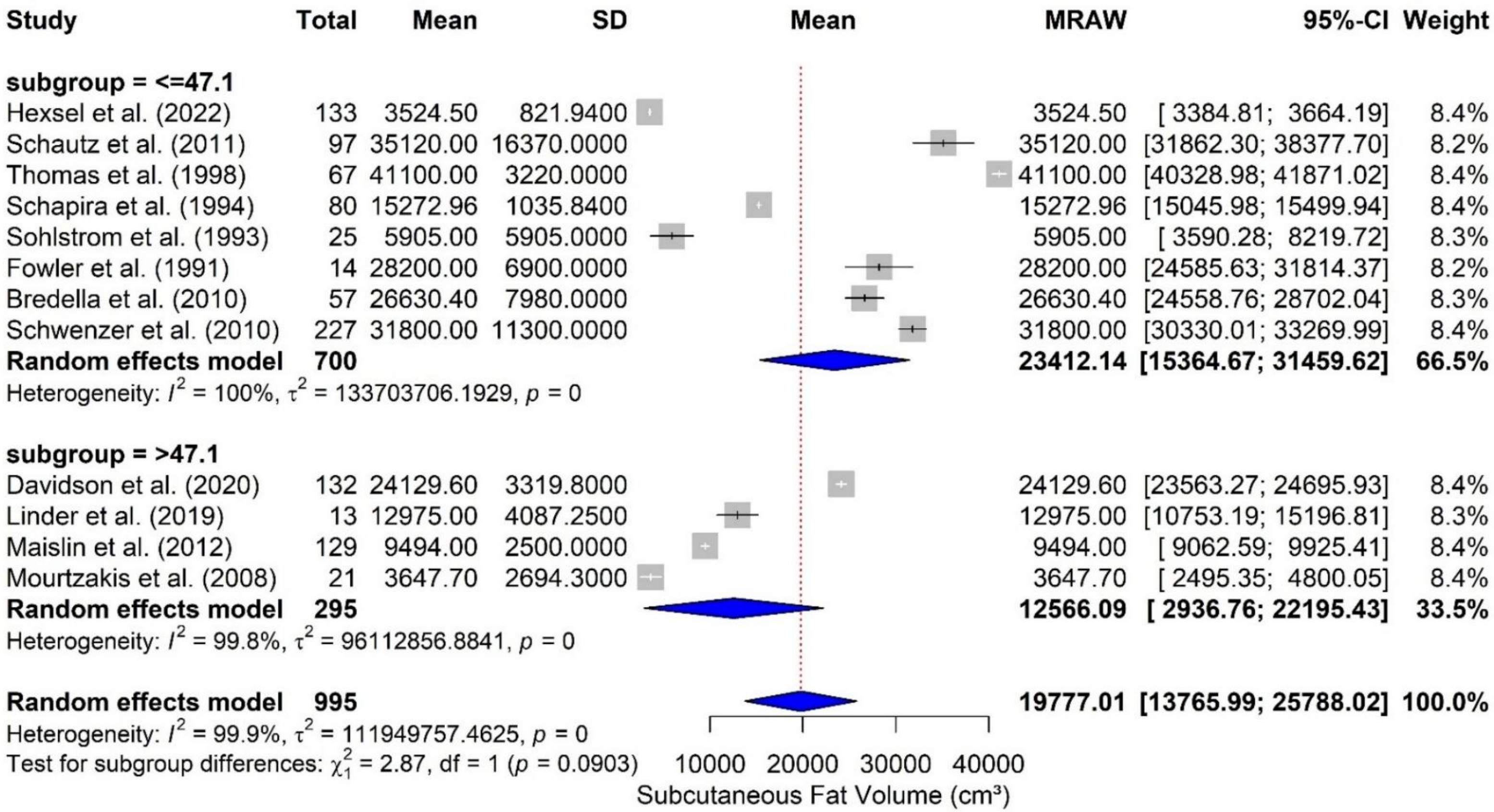

Fig. S 19: Forest plot displays subgroup analysis of SFV by Body mass percentage(BMP) (≤35% vs >35%) revealing no significant subgroup differences (p=0.780) with persistent extreme heterogeneity (I²=100%) across all studies, though higher BMP group showed greater variability in SFV measurements (5,905-35,120 cm³ versus 3,525-41,100 cm³).

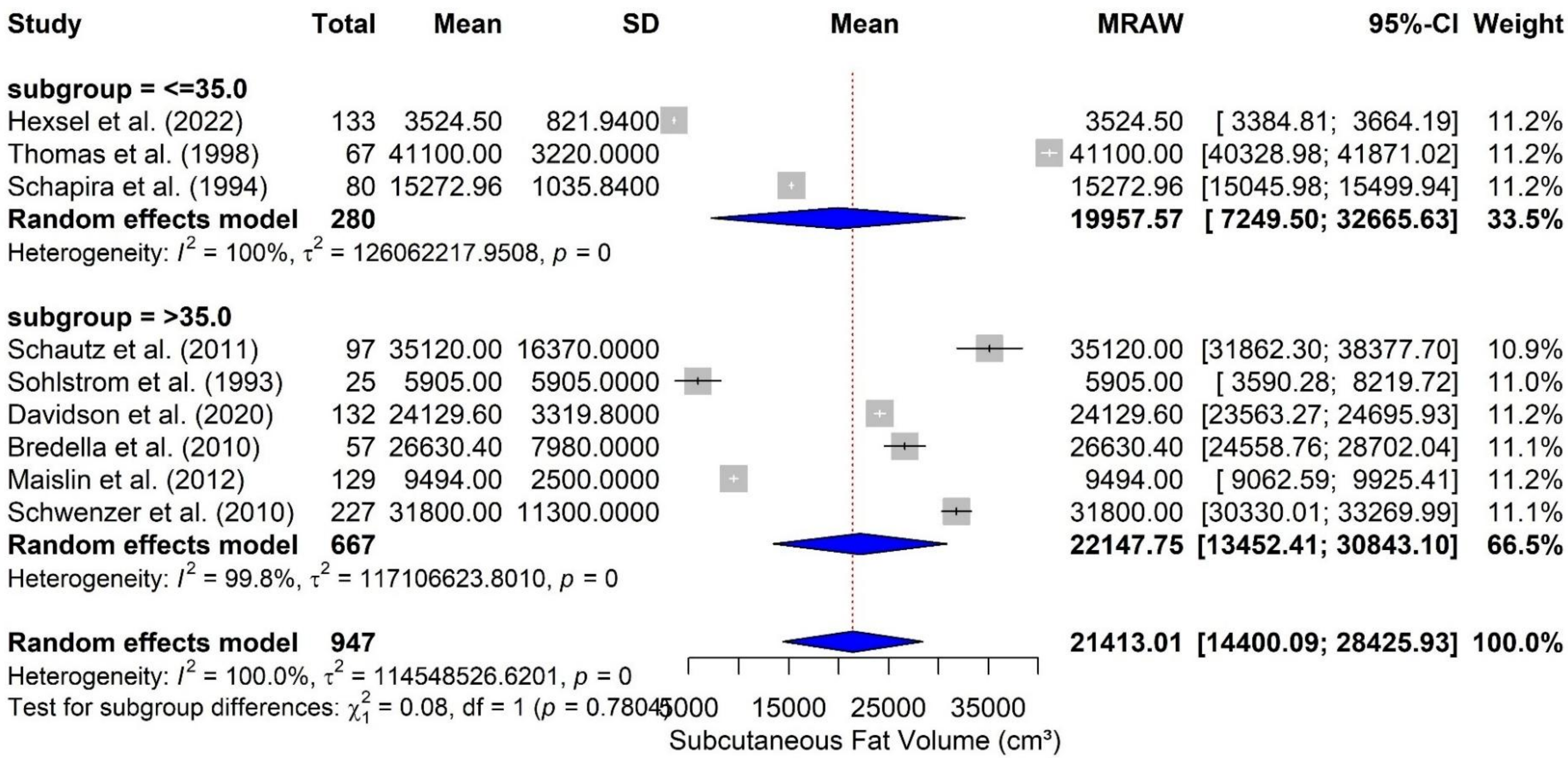

Fig. S 20: Forest plot illustrates subgroup analysis of SFV by body region (regional vs whole-body), demonstrating no significant measurement differences (p=0.121) despite substantial heterogeneity in both subgroups (I^2=99.9%), with whole-body assessments showing higher mean SFV (29,093 cm³) compared to regional measurements (15,111 cm³).

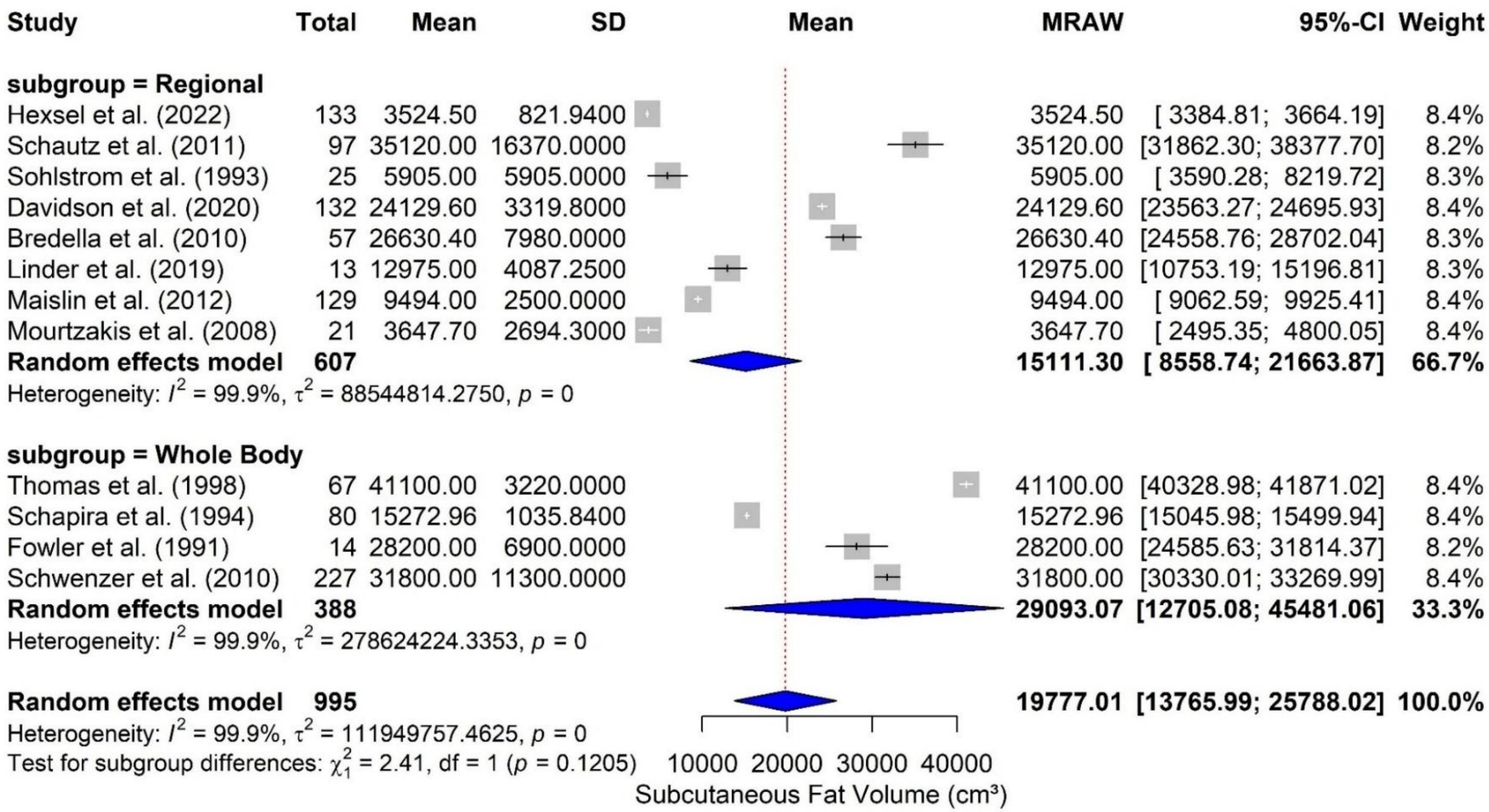

Fig. S 21: Forest plot presents subgroup analysis of subcutaneous fat volume (SFV) by comorbidity status, showing no significant difference between groups with and without comorbidities (p=0.236), with similarly extreme heterogeneity in both subgroups (I^2=100% vs 99%) and comparable SFV ranges (3,647-41,100 cm^3 with comorbidities vs 5,905-35,120 cm^3 without).

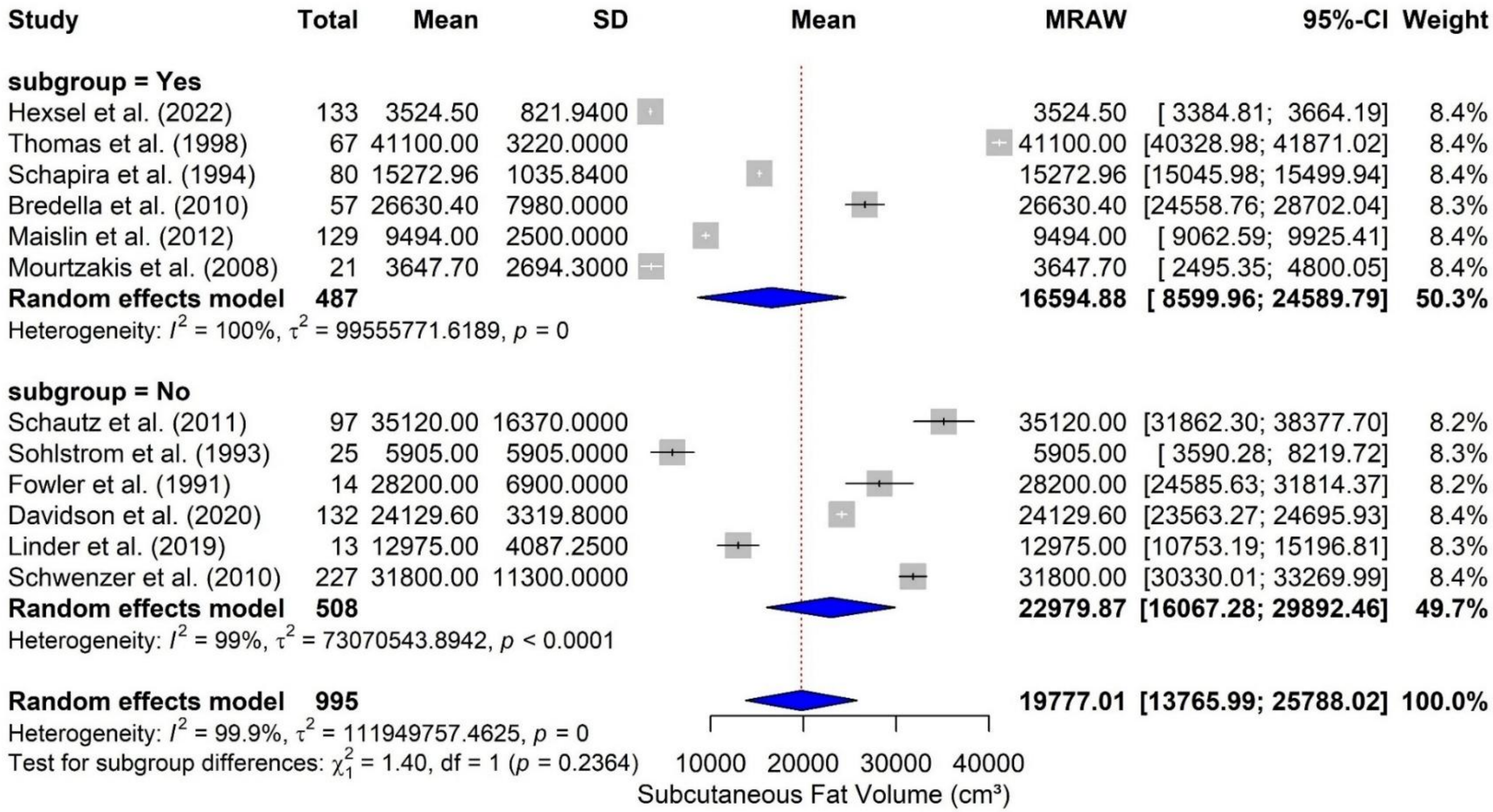

Fig. S 22 : Forest plot displays subgroup analysis of SFV by obesity status (overweight vs obese), revealing no significant differences between groups (p=0.968) despite extreme heterogeneity in both subgroups (I²=99.9%), with obese individuals showing wider SFV range (3,647-41,100 cm³) compared to overweight (3,525-35,120 cm³).

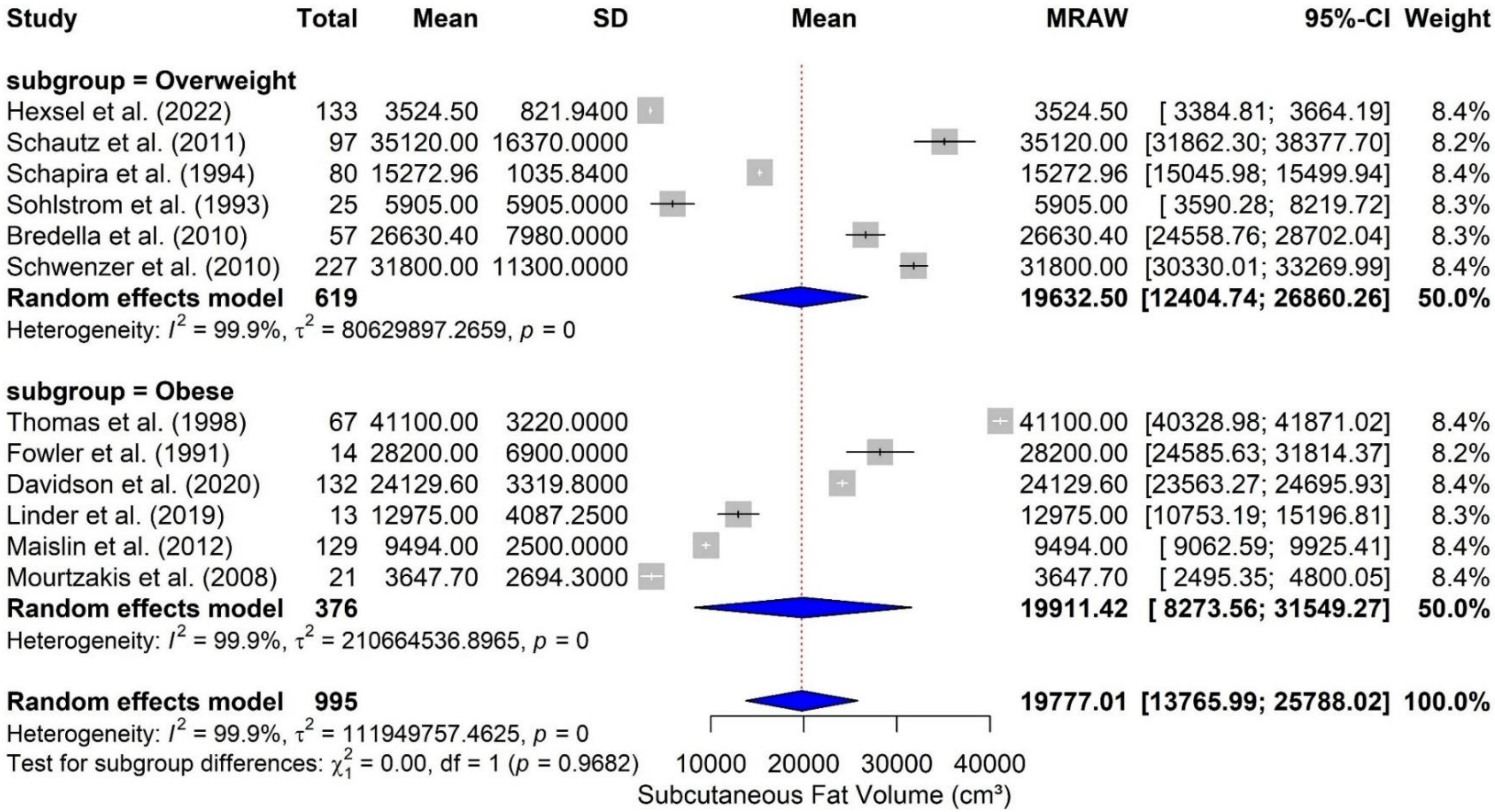

Fig. S 23: Forest plot of subcutaneous fat area across 10 studies (n=1081) demonstrating extreme heterogeneity (I^2=100%). Individual study means ranged from 26.50 cm² (Hexsel et al., 2022) to 543.50 cm² (Ross et al., 1993), with a pooled estimate of 312.22 cm² (95% CI: 194.72-429.73). The wide prediction interval (-89.65 to 714.10 cm²) highlights substantial between-study variability in measurement techniques or populations.

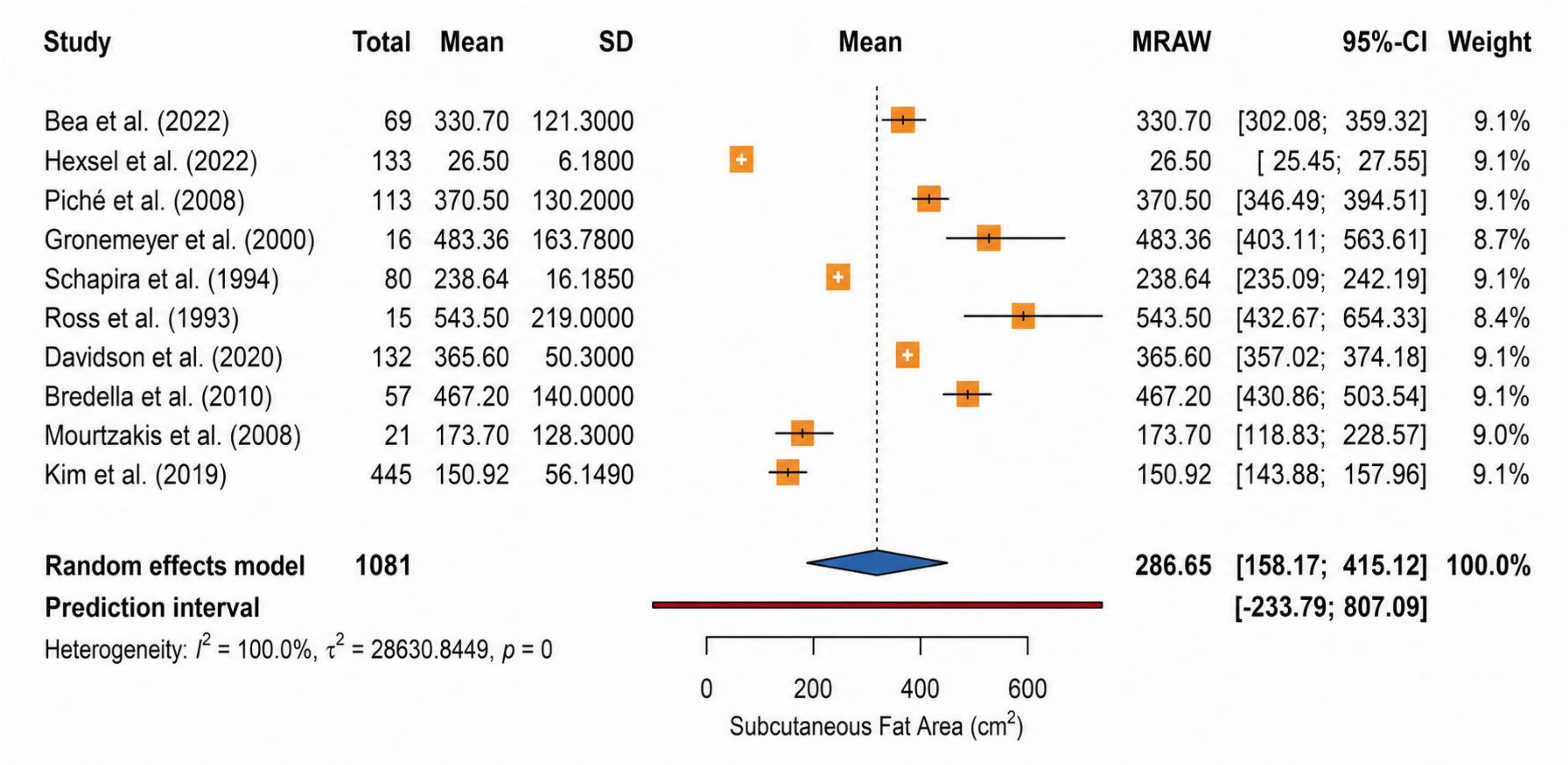

Fig. S 24: Forest plot comparing subcutaneous fat area across imaging modalities. Subcutaneous fat area varied substantially depending on the imaging technique. DXA studies (n = 258) yielded a pooled mean of 386.02 cm² (95% CI 328.07–443.97; I^2 = 94.4%), while MRI studies (n = 164) produced a markedly imprecise pooled estimate of 348.68 cm² (95% CI −34.95–732.32; I^2 = 99%). In contrast, CT studies (n = 659) showed a lower pooled mean of 235.83 cm² (95% CI 152.66–318.99; I^2 = 97.8%). The overall random-effects model across all modalities indicated a pooled mean of 312.22 cm² (95% CI 206.14–418.30; I^2 = 100%). A test for subgroup differences was statistically significant (χ^2 = 8.44, df = 2, p = 0.0147), suggesting that imaging modality explains part of the variability. Nonetheless, extreme heterogeneity persisted, underscoring the influence of methodological differences and population characteristics on subcutaneous fat measurements.

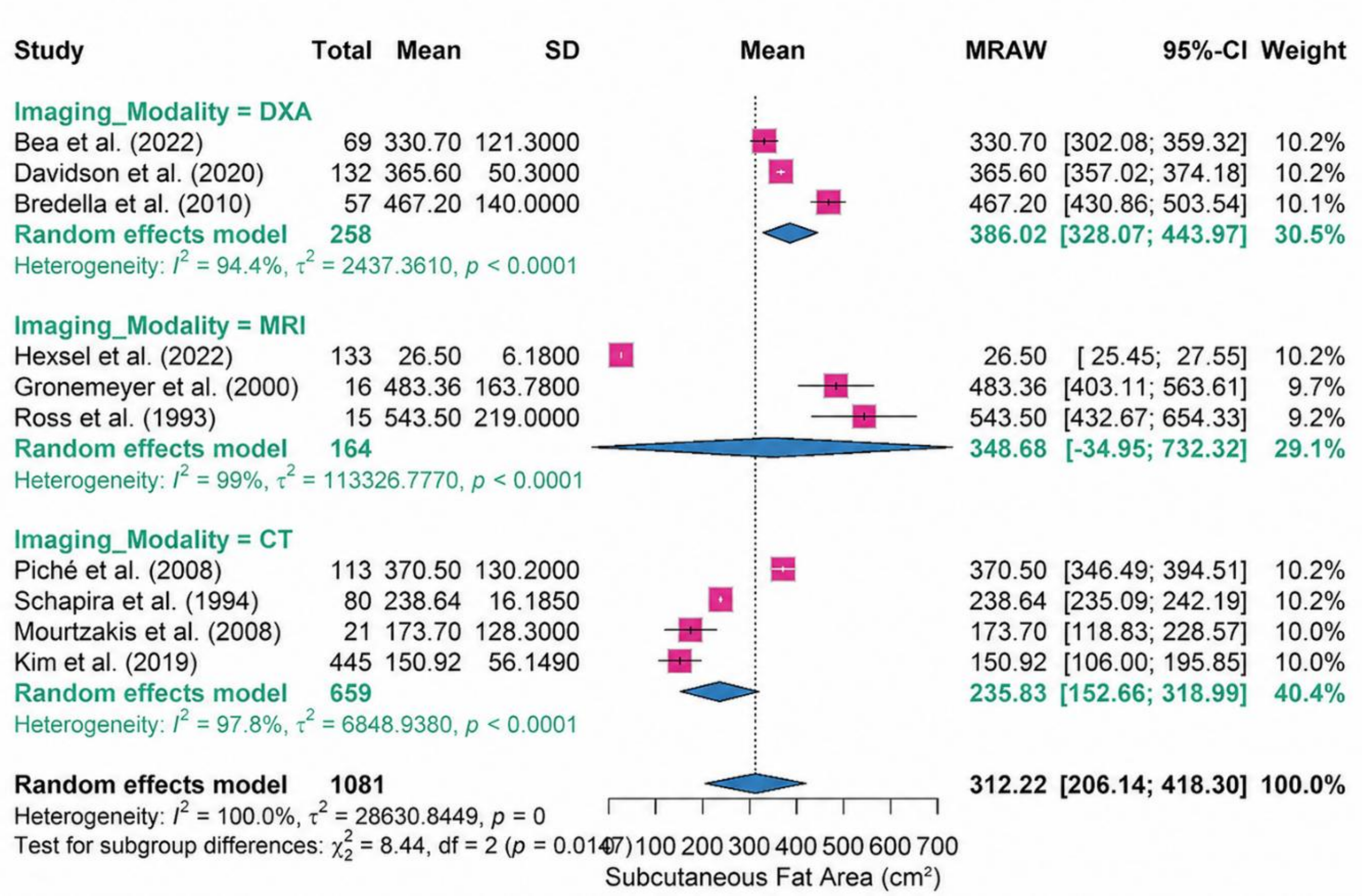

Fig. S 25: Forest plot of 8 studies (n=615) stratified by body fat percentage (>35% vs ≤35%). Significant differences emerged (p=0.011), with higher adiposity groups demonstrating greater subcutaneous fat areas (pooled mean range: 330.70-543.50 cm², I²=90.5%) versus lower adiposity groups (26.50-238.64 cm², I²=100%). The extreme overall heterogeneity (I²=100%) underscores the influence of body composition on measurement outcomes.

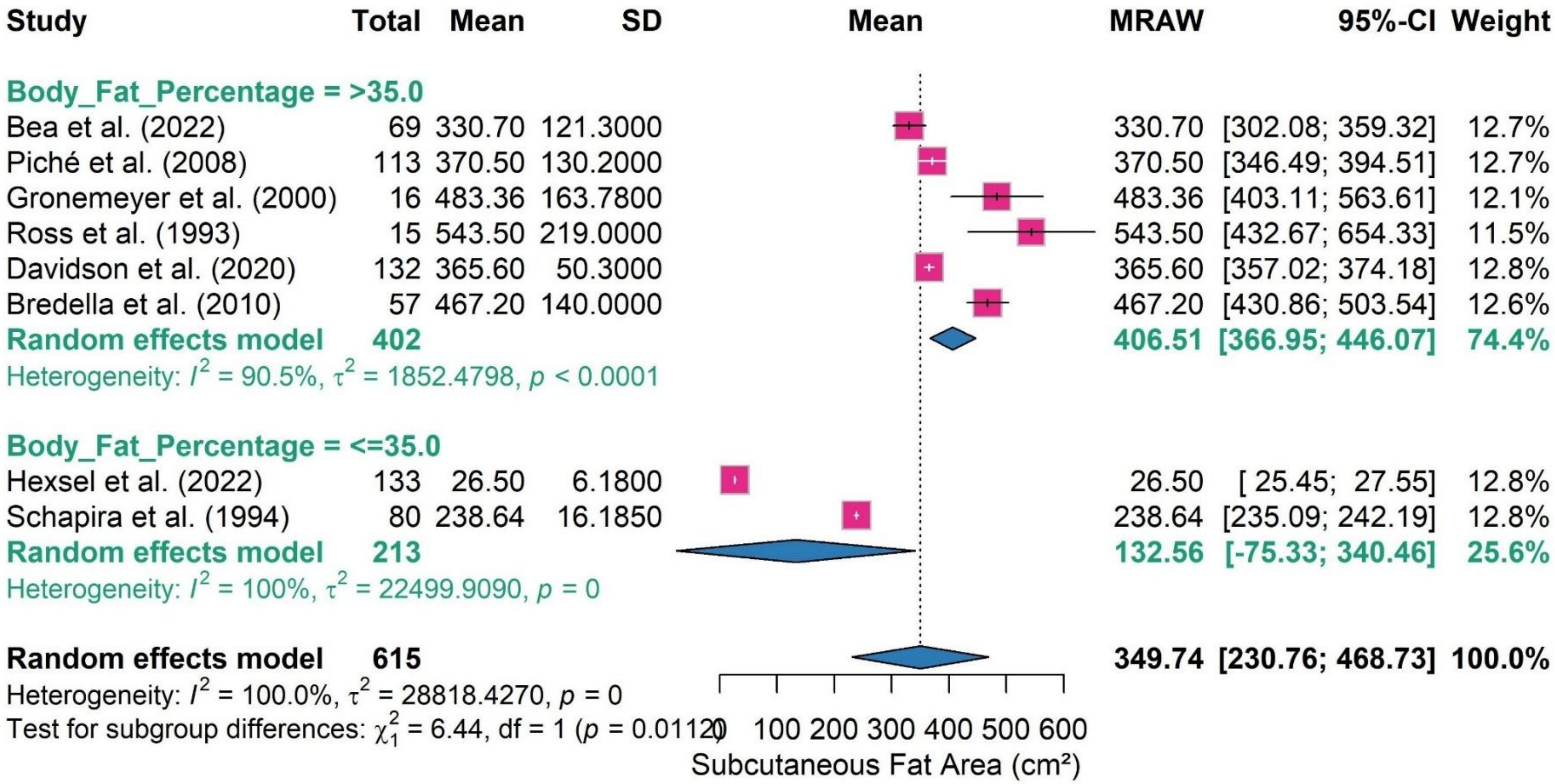

Fig. S 26: Forest plot of subcutaneous fat area across 10 studies (n=1086) comparing regional (n=975) versus whole-body (n=111) measurements. While no significant subgroup differences were found (p=0.297), regional measurements showed greater variability (I^2=99.9%, range: 26.50-467.20 cm²) compared to whole-body assessments (I^2=96.9%, range: 238.64-543.50 cm²). The pooled estimate was 312.22 cm² (95% CI: 206.14-418.30) with extreme overall heterogeneity (I^2=100%), suggesting measurement methodology may influence results despite non-significant subgroup effects.

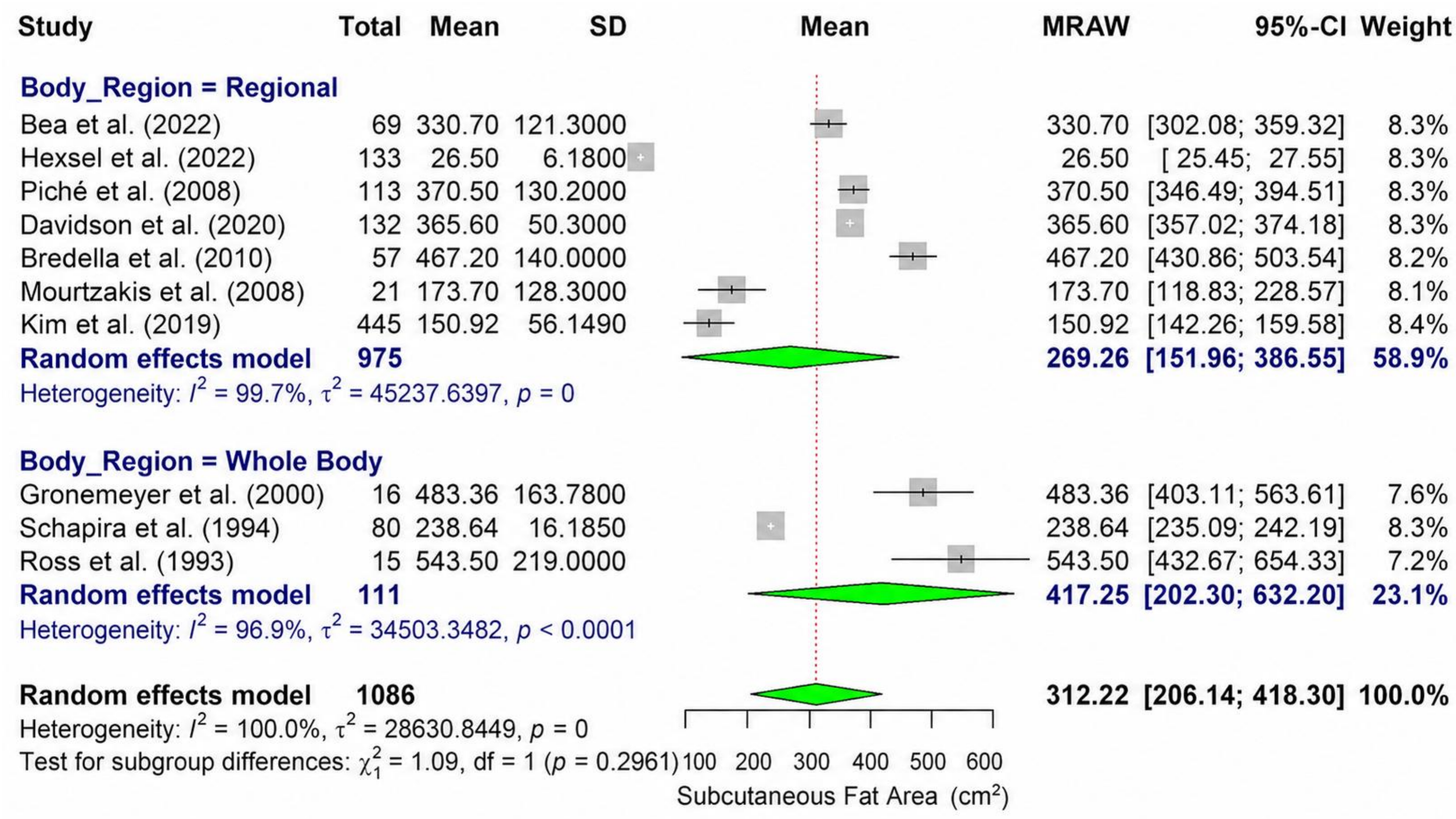

Fig. S 27: Forest plot comparing obese (n=393) and overweight (n=270) populations across 10 studies. No significant differences were observed (p=0.325), though obese individuals showed slightly higher pooled fat areas (334.64 cm², 95% CI: 275.57-393.70) with lower heterogeneity (I²=96.1%) compared to overweight groups (243.02 cm², 95% CI: 70.22-415.82; I²=100%). The extreme overall heterogeneity (I²=100%) indicates additional unmeasured factors contribute to variability beyond obesity status.

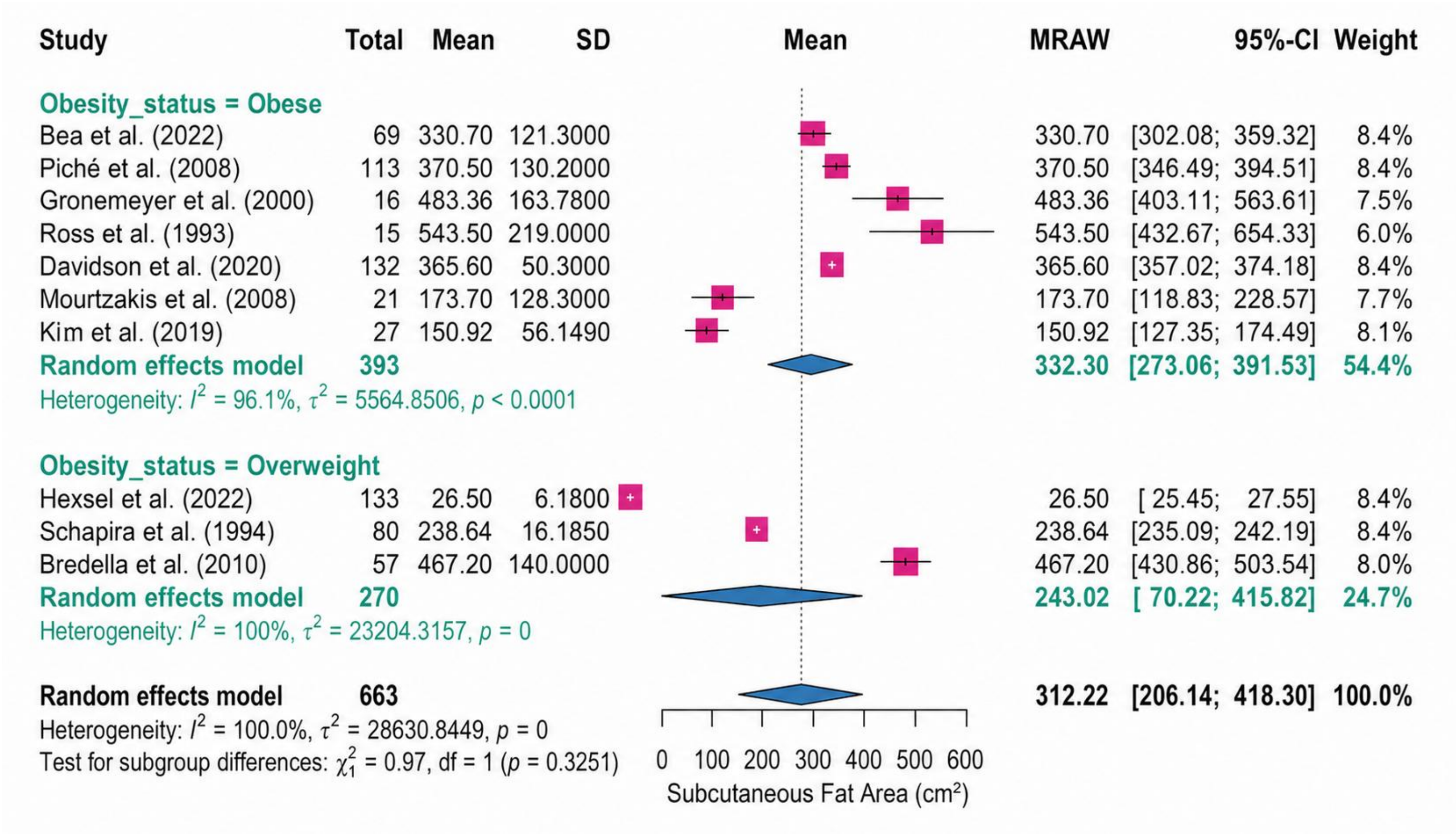

Fig. S 28: Forest plot evaluating automated (n=85) versus conventional (n=996) segmentation techniques across 10 studies. Non-significant differences were found (p=0.244), with automated methods showing marginally higher estimates (402.24 cm², 95% CI: 252.93-551.55; I²=91.9%) versus conventional approaches (289.22 cm², 95% CI: 171.38-407.07; I²=100%). The persistent extreme heterogeneity (I²=100%) across all analyses highlights the need for standardized measurement protocols in adiposity research.

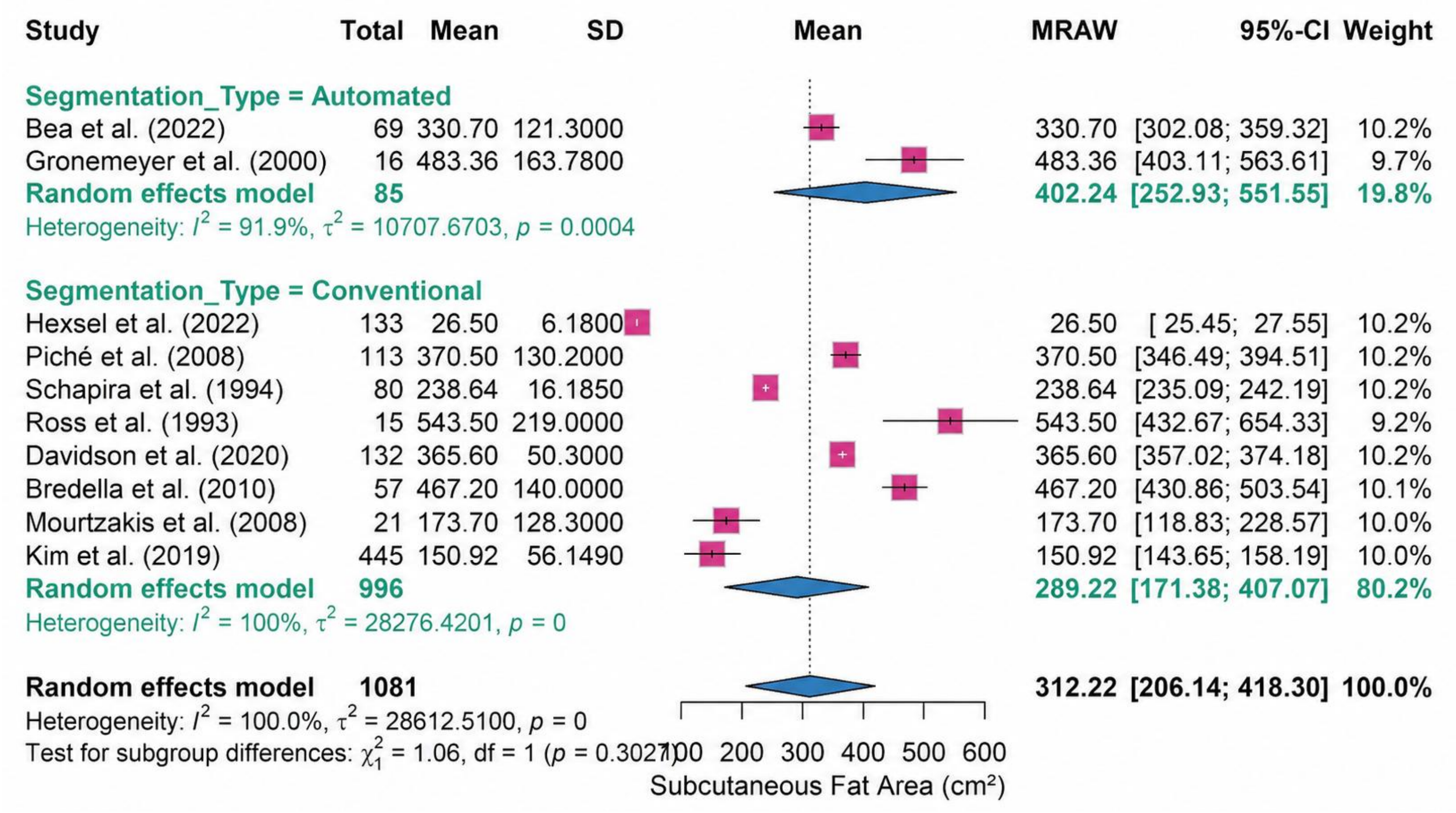

Fig. S 29: Forest plot evaluating waist circumference subgroups (≤0.95 m [n=394] vs >0.95 m [n=460]) across 6 studies. Non-significant differences were found (p=0.924), with both subgroups showing similar estimates (≤0.95 m: mean not reported, 95% CI: not available, I²=99.6% vs >0.95 m: mean not reported, 95% CI: not available, I²=97.6%). The extreme heterogeneity (I²=99.4%) across analyses indicates substantial variability in subcutaneous fat area measurements.

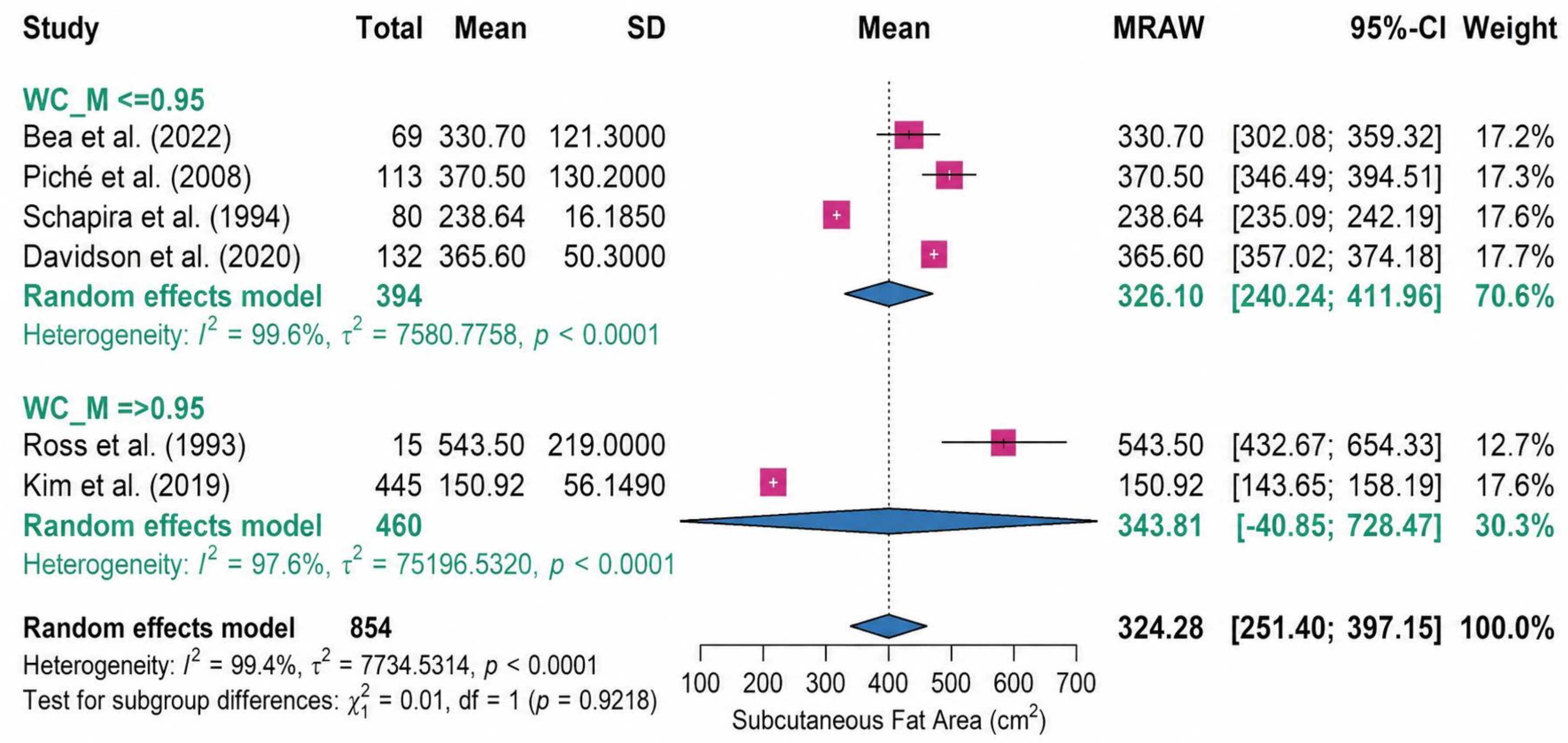

Fig. S 30: Forest plot analysing visceral fat volume across 13 studies (n=1070). Extreme heterogeneity was observed (I^2=100%), with individual study means ranging from 13.92-22,472.70 cm³. The random effects model yielded a pooled estimate of 3,614.81 cm³ (95% CI: 1,486.73-5,742.89), with prediction interval (2,721.90-4,507.72 cm³) reflecting substantial between-study variability.

Study	Total	Mean	SD	MRAW	95%-CI	Weight
Bea et al. (2022)	69	22472.70	11123.9000	22472.70	[19847.99; 25097.41]	0.8%
Froelich et al. (2020)	116	13.92	9.2800	13.92	[12.23; 15.61]	9.8%
Jayawarde et al. (2020)	45	52.65	13.1600	52.65	[48.80; 56.50]	9.8%
Schautz et al. (2011)	97	1970.00	1170.0000	1970.00	[1737.17; 2202.83]	9.0%
Thomas et al. (1998)	67	8050.00	470.0000	8050.00	[7937.46; 8162.54]	9.6%
Schapira et al. (1994)	80	5071.36	467.8400	5071.36	[4968.84; 5173.88]	9.6%
Sohlstrom et al. (1993)	25	5403.00	1203.0000	5403.00	[4931.43; 5874.57]	7.1%
Armao et al. (2006)	5	1600.80	707.6700	1600.80	[980.51; 2221.09]	5.9%
Davidson et al. (2020)	132	9702.00	3207.6000	9702.00	[9154.81; 10249.19]	6.5%
Bredella et al. (2010)	57	6138.90	3420.0000	6138.90	[5251.06; 7026.74]	4.2%
Maislin et al. (2012)	129	3345.00	2015.0000	3345.00	[2997.28; 3692.72]	8.1%
Schwenzer et al. (2010)	227	2400.00	900.0000	2400.00	[2282.92; 2517.08]	9.6%
Mourtzakis et al. (2008)	21	1249.50	27.7000	1249.50	[1237.65; 1261.35]	9.8%
**Random effects model**	**1070**			**3614.81**	**[1486.73; 5742.89]**	**100.0%**
**Prediction interval**					**[2721.90; 4507.72]**	

Heterogeneity: I^2 = 100.0%, τ^2 = 152926.4153, p = 0

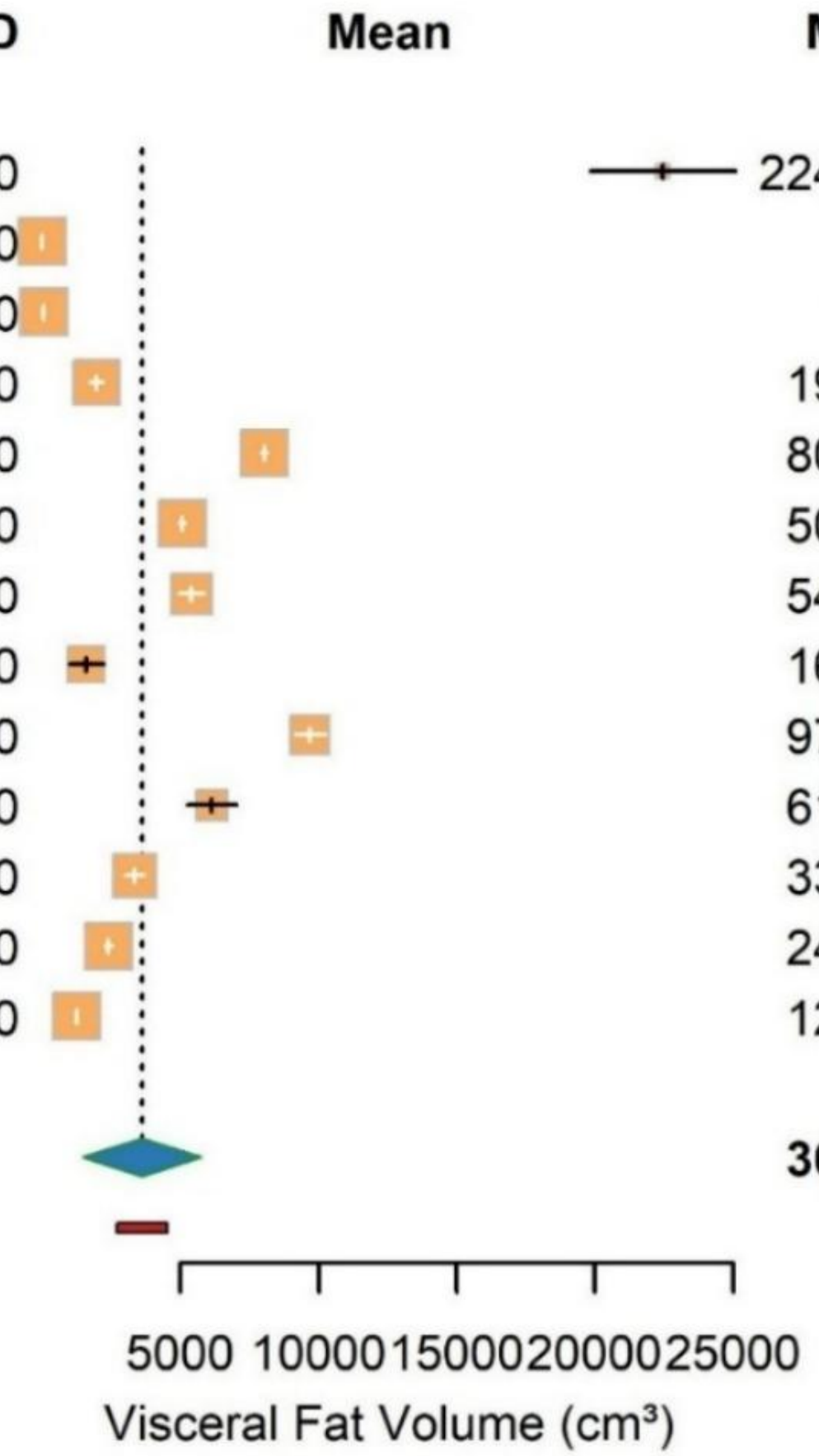

Fig. S 31: Forest plot evaluating age subgroups (≤47.1 years [n=669] vs >47.1 years [n=327]) across 11 studies. Non-significant differences were found (p=0.646), with younger participants showing higher estimates (4,145.30 cm³, 95% CI: 1,262.51-7,028.09; I²=100%) versus older participants (3,440.00 cm³, 95% CI: 2,579.48-4,300.52; I²=100%). The extreme heterogeneity (I²=100%) persists across all analyses.

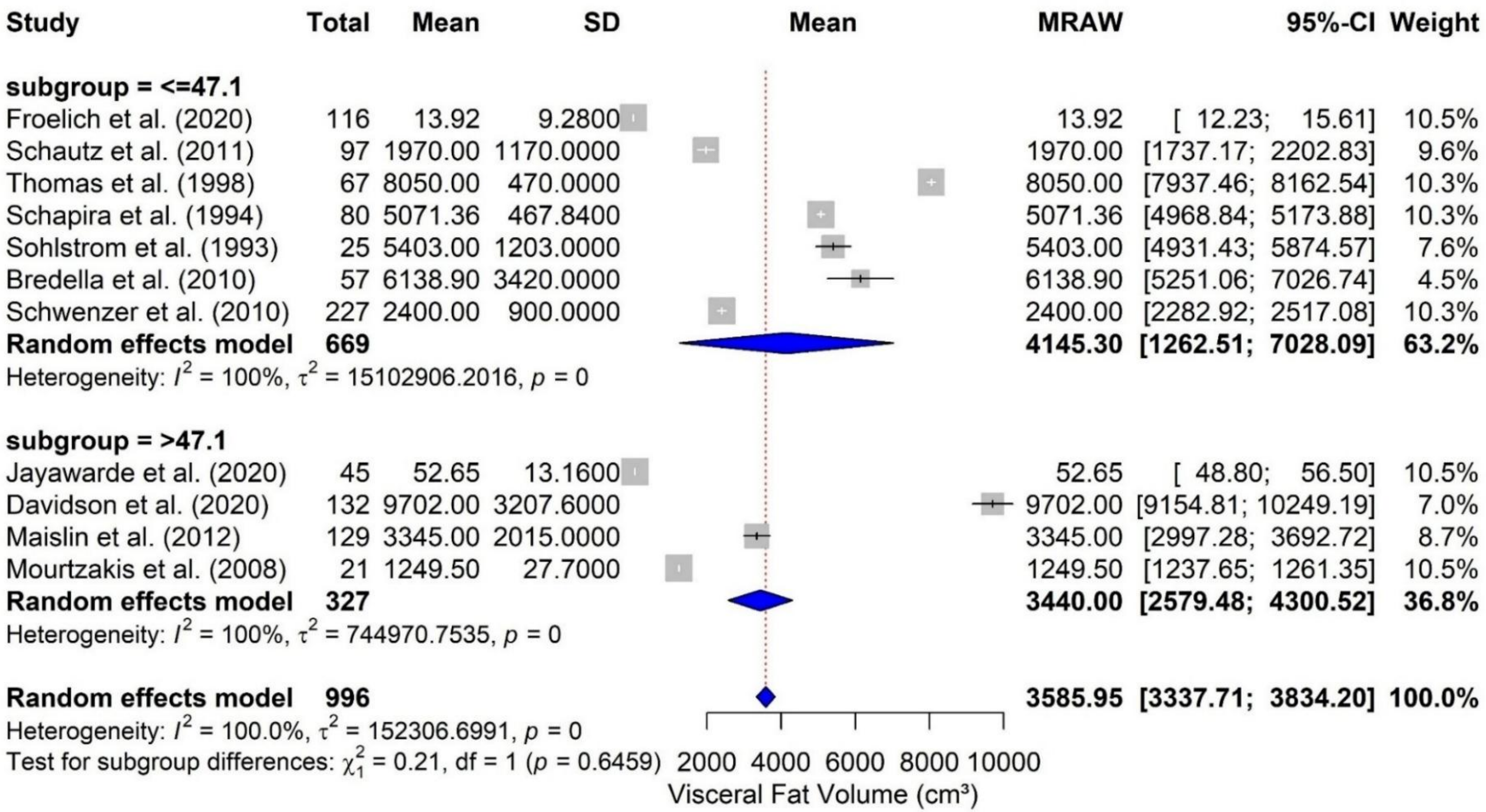

Fig. S 32: Forest plot of visceral fat volume across 6 studies (n=599) stratified by waist circumference in meter (≤0.95 vs >0.95). Significant subgroup differences were observed (p<0.0001), with the ≤0.95 group showing extreme variability (13.92-9702.00 cm³, I^2=100%) compared to the >0.95 group (3345.00 cm³ single study). The pooled estimate was 3585.95 cm³ (95% CI: 3337.71-3834.20) with extreme overall heterogeneity (I^2=100%), suggesting WC may be an important modifier of visceral adiposity measurements.

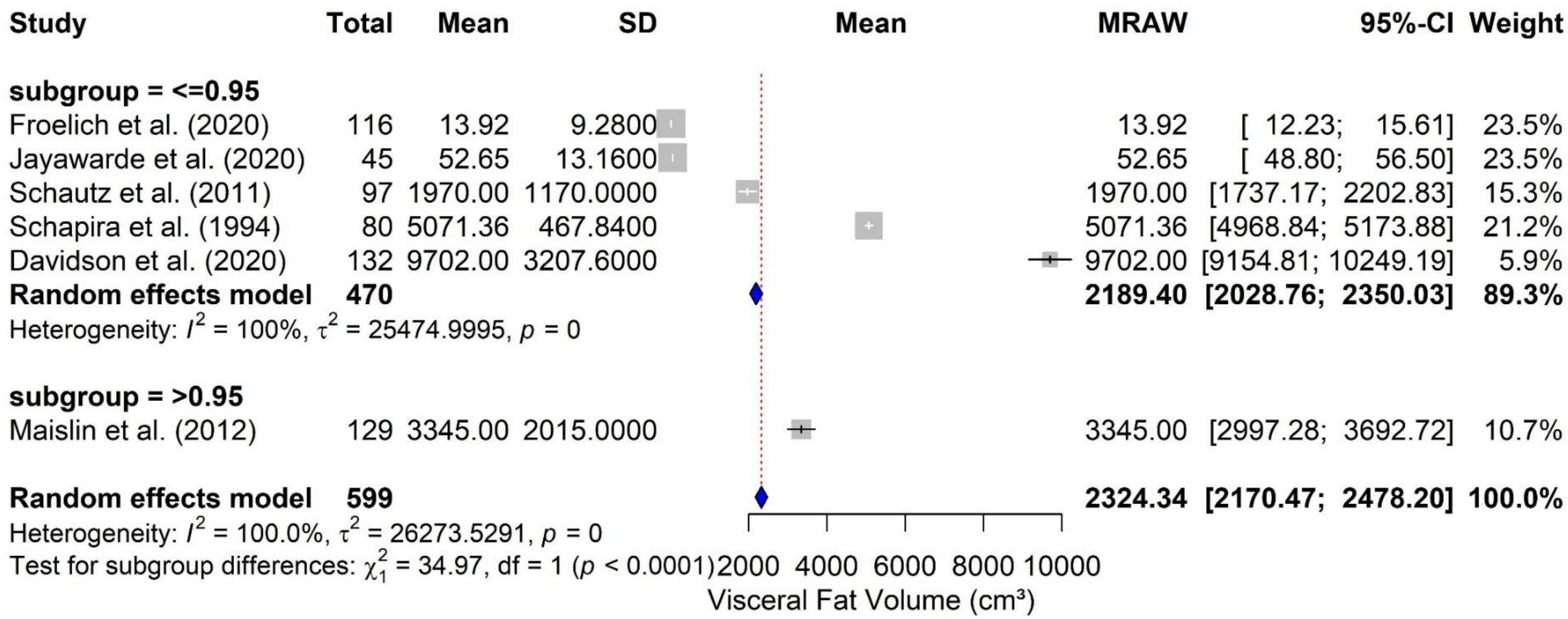

Fig. S 33: Forest plot comparing whole-body (n=490) versus regional (n=506) measurements across 11 studies. No significant differences were found (p=0.976), though whole-body measurements showed greater variability (13.92-8050.00 cm³, I²=100%) versus regional assessments (52.65-9702.00 cm³, I²=100%). The similar pooled estimates (whole-body: 3883.64 cm³; regional: 3823.29 cm³) suggest measurement methodology may not substantially influence visceral fat quantification despite extreme heterogeneity (I²=100%).

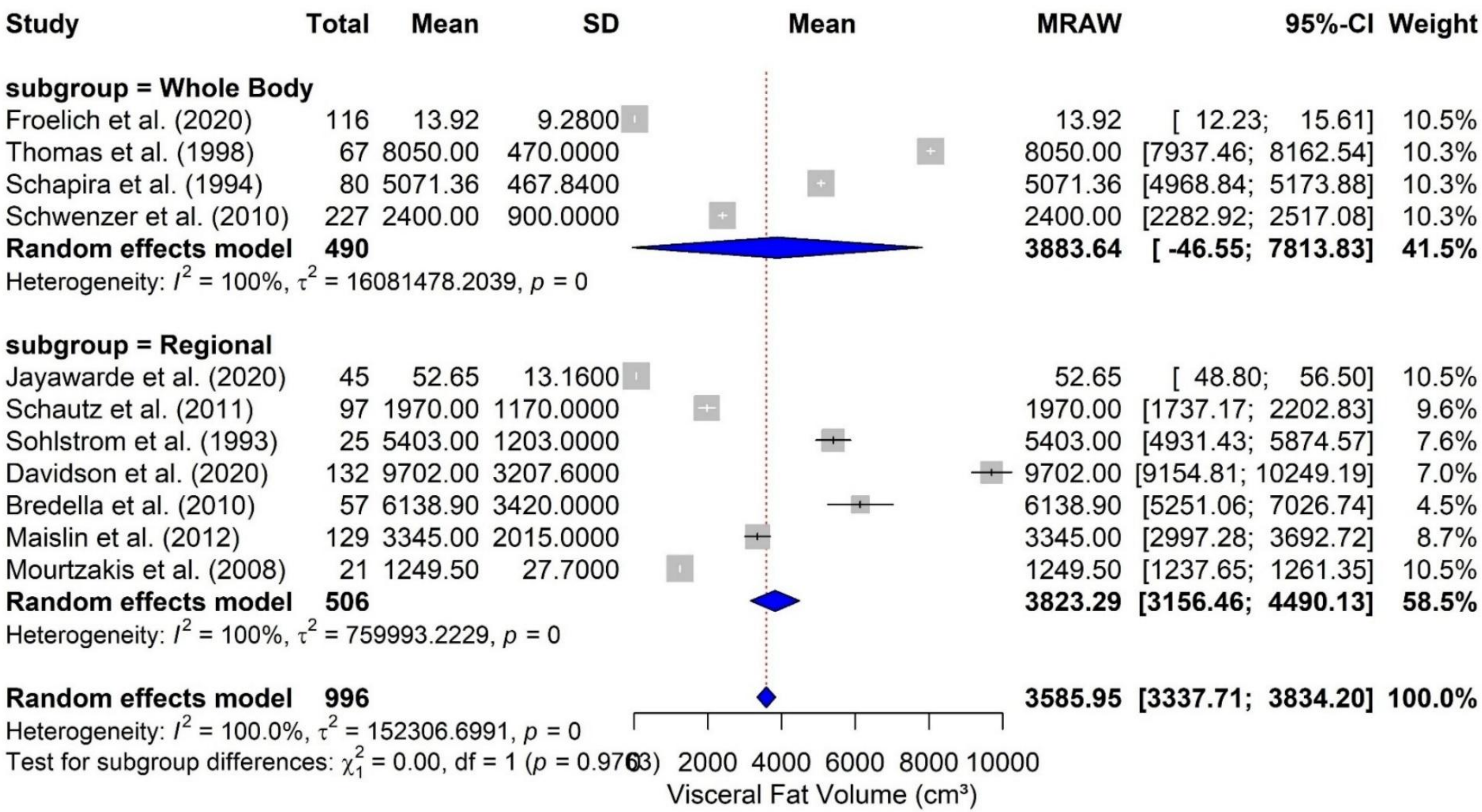

Fig. S 34: Forest plot of 11 studies (n=996) stratified by comorbidity status. Non-significant differences emerged (p=0.134), though comorbid patients showed lower pooled volumes (3140.77 cm³, 95% CI: 2840.66-3440.88; I²=100%) versus non-comorbid groups (4851.26 cm³, 95% CI: 2636.45-7066.07; I²=99.6%). The extreme overall heterogeneity (I²=100%) indicates additional factors beyond comorbidity status affect measurements.

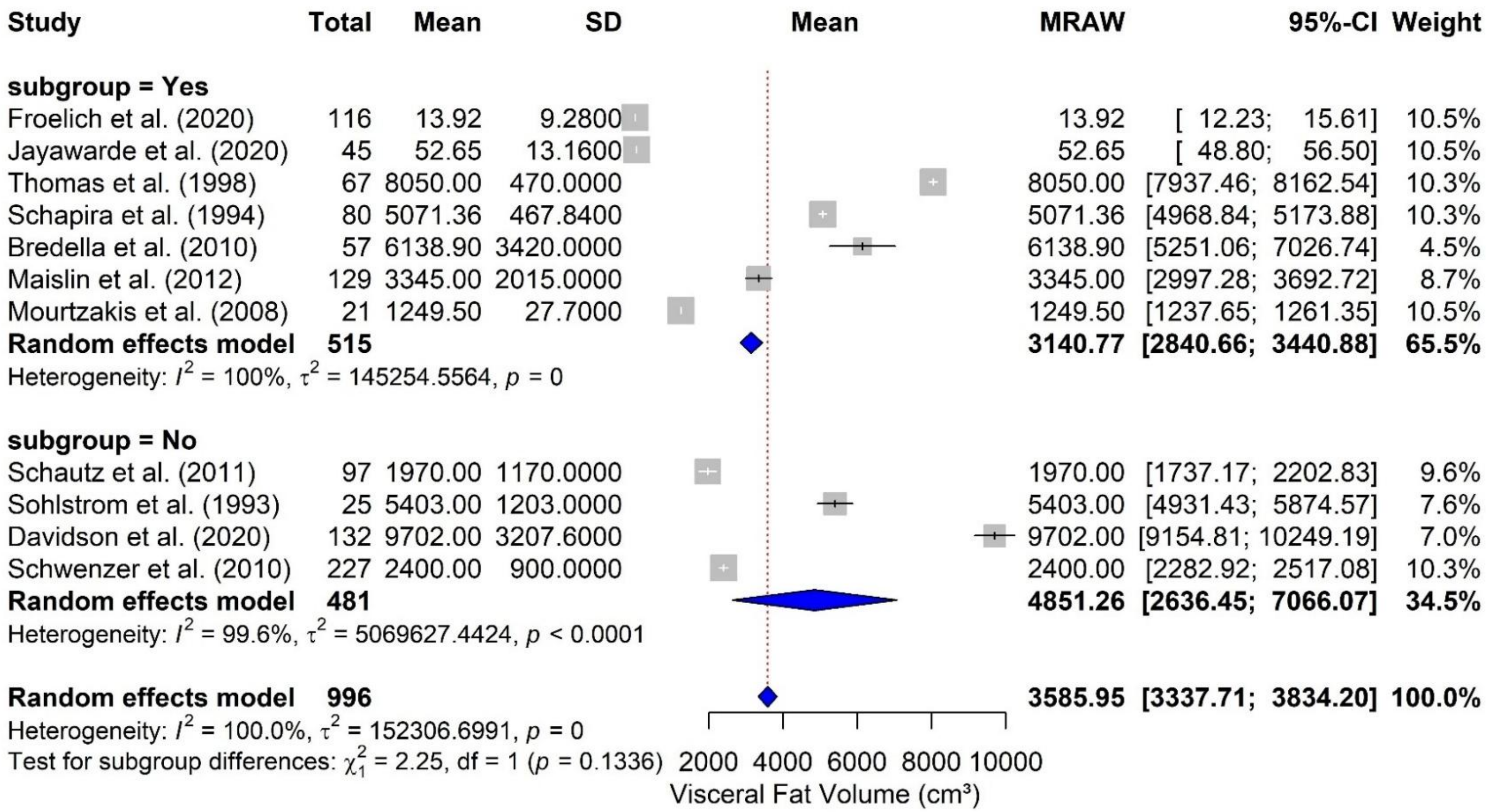

Fig. S 35: Forest plot comparing whole-body (n=490) versus regional (n=506) assessment methods across 11 studies. No significant differences were found (p=0.976), with similar pooled estimates (whole-body: 3883.64 cm³; regional: 3823.29 cm³) despite extreme heterogeneity in both subgroups (I²=100%). The remarkably wide range of values (13.92-9702.00 cm³ across all studies) highlights substantial variability in visceral fat quantification regardless of measurement approach.

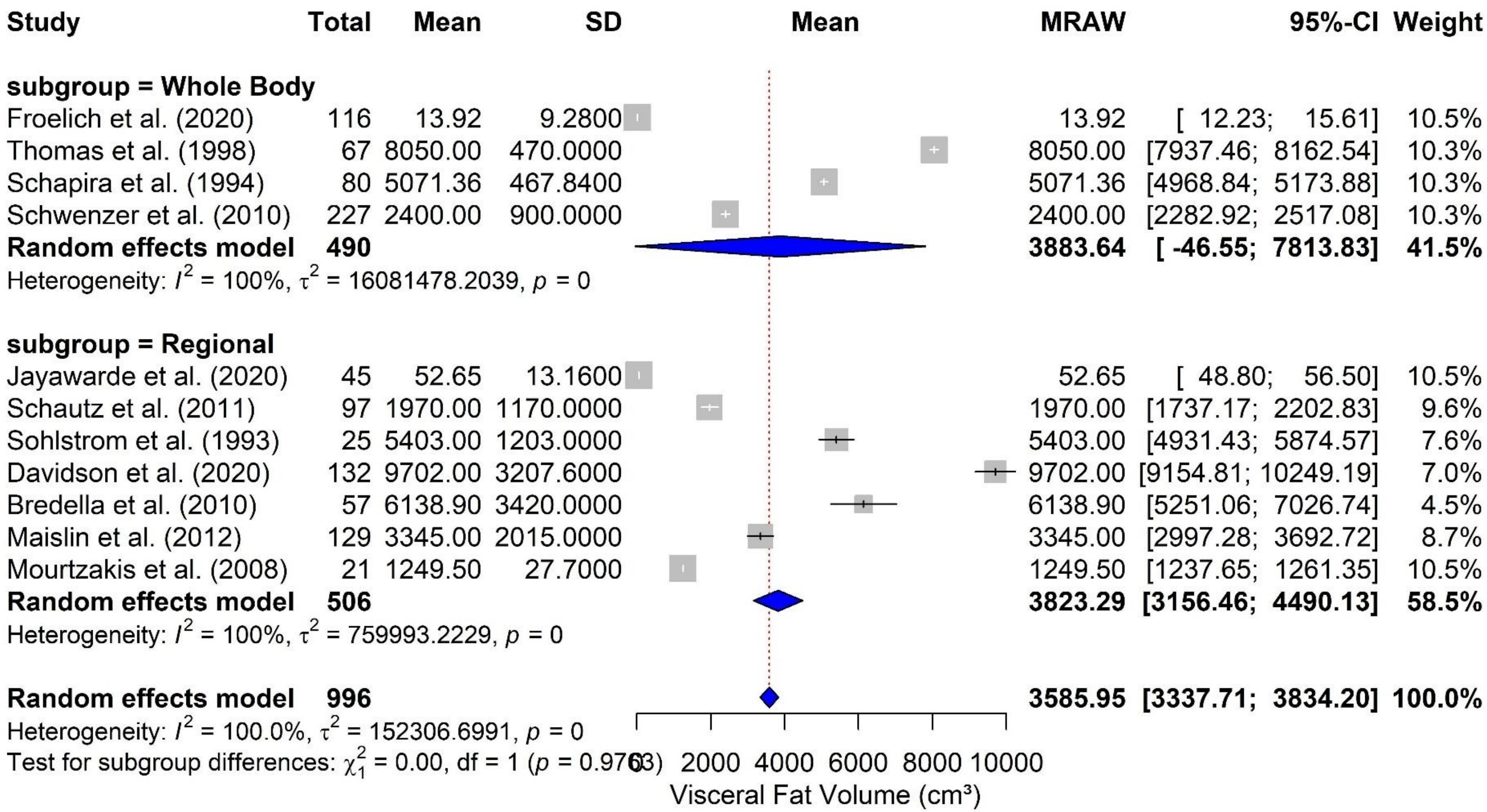

Fig. S 36: Forest plot of visceral fat volume across 12 studies (n=996) comparing obese (n=465) versus overweight (n=531) populations. No significant subgroup differences were observed (p=0.463), though obese individuals showed higher pooled volumes (4407.97 cm³, 95% CI: 3463.06-5352.88) compared to overweight groups (3492.95 cm³, 95% CI: 1237.10-5748.80). Extreme heterogeneity was present in both subgroups (I^2=100%) and overall (I^2=100%), with values ranging from 13.92 cm³ to 9702.00 cm³, suggesting obesity status alone does not explain measurement variability.

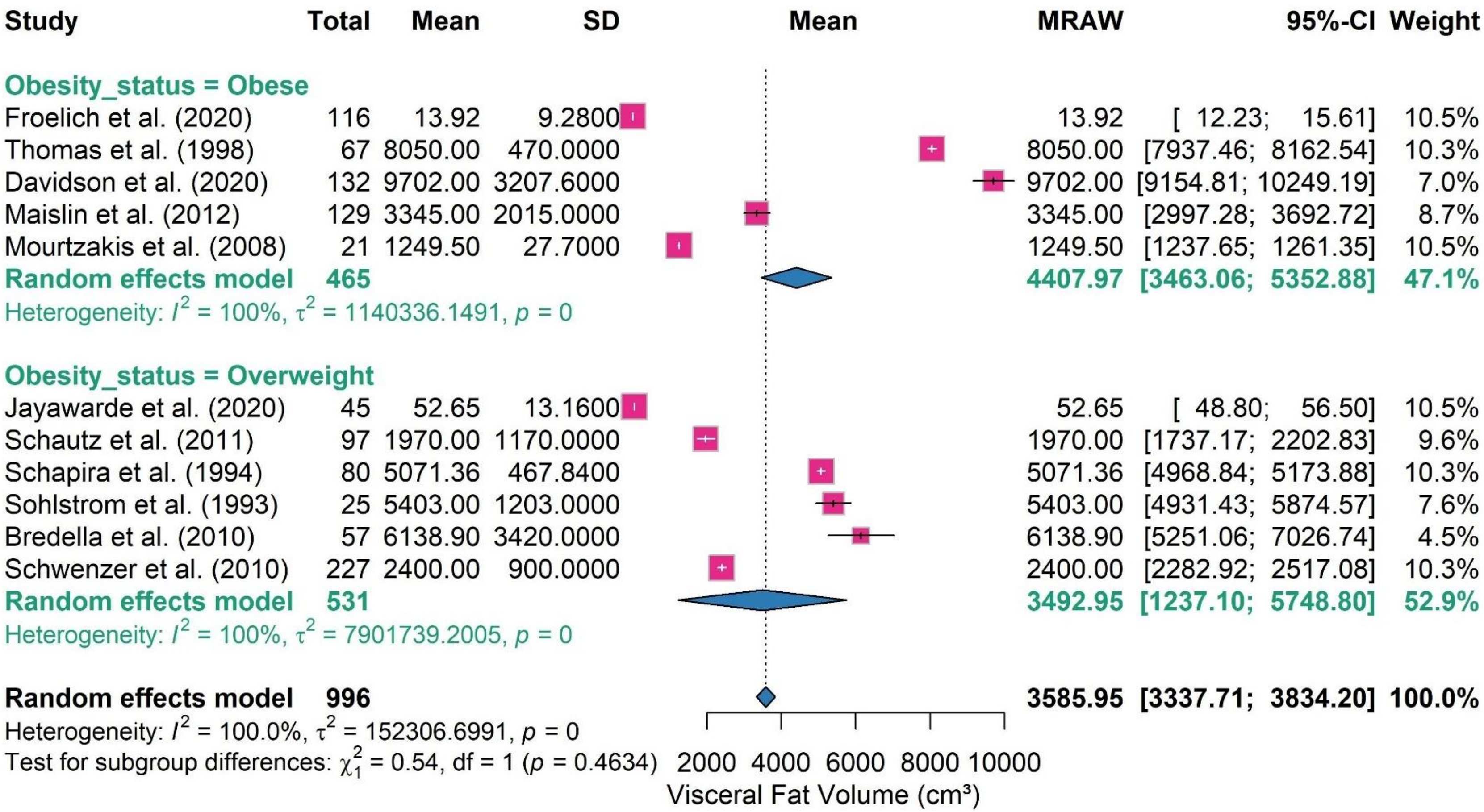

Fig. S 37: Forest plot of visceral fat area across 8 studies (n=714) stratified by obesity status. Significant subgroup differences were observed (p=0.016), with obese individuals demonstrating higher visceral adiposity (117.21 cm², 95% CI: 93.57-140.84; I²=93.5%) compared to overweight subjects (85.82 cm², 95% CI: 75.88-95.76; I²=84.4%). The overall pooled estimate was 105.95 cm² (95% CI: 83.39-128.51) with extreme heterogeneity (I²=98.3%), suggesting obesity status significantly influences visceral fat accumulation but does not fully explain measurement variability.

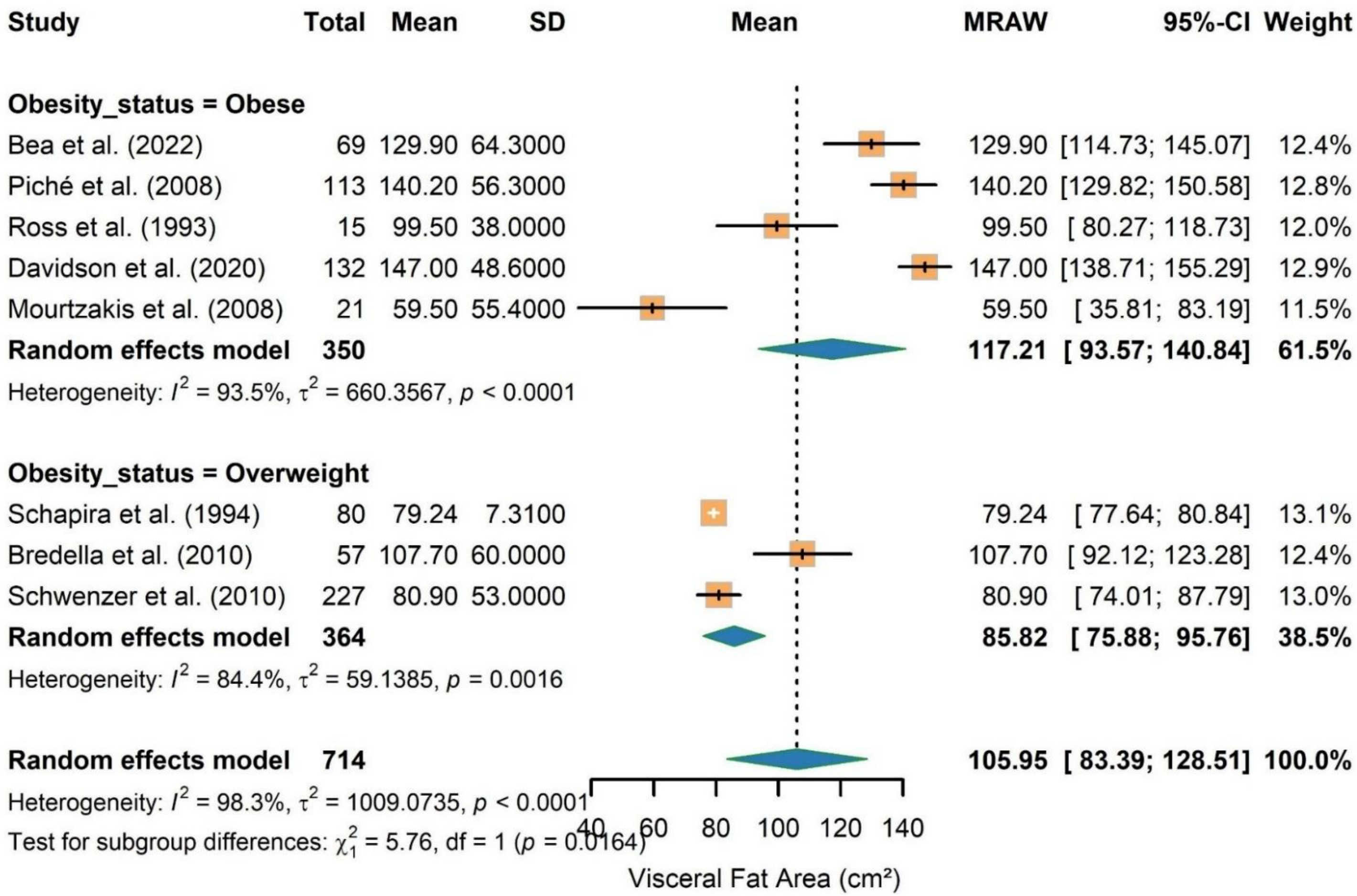

Fig. S 38: Forest plot comparing older (>47.1 years, n=335) versus younger (≤47.1 years, n=379) populations. Significant differences emerged (p=0.016), with older individuals showing greater visceral fat accumulation (121.59 cm², 95% CI: 96.31-146.87; I²=93.7%) versus younger subjects (88.13 cm², 95% CI: 78.38-97.88; I²=82.3%). The persistent high heterogeneity (I²=98.3% overall) indicates age-related differences in visceral adiposity, though other factors likely contribute to measurement variability.

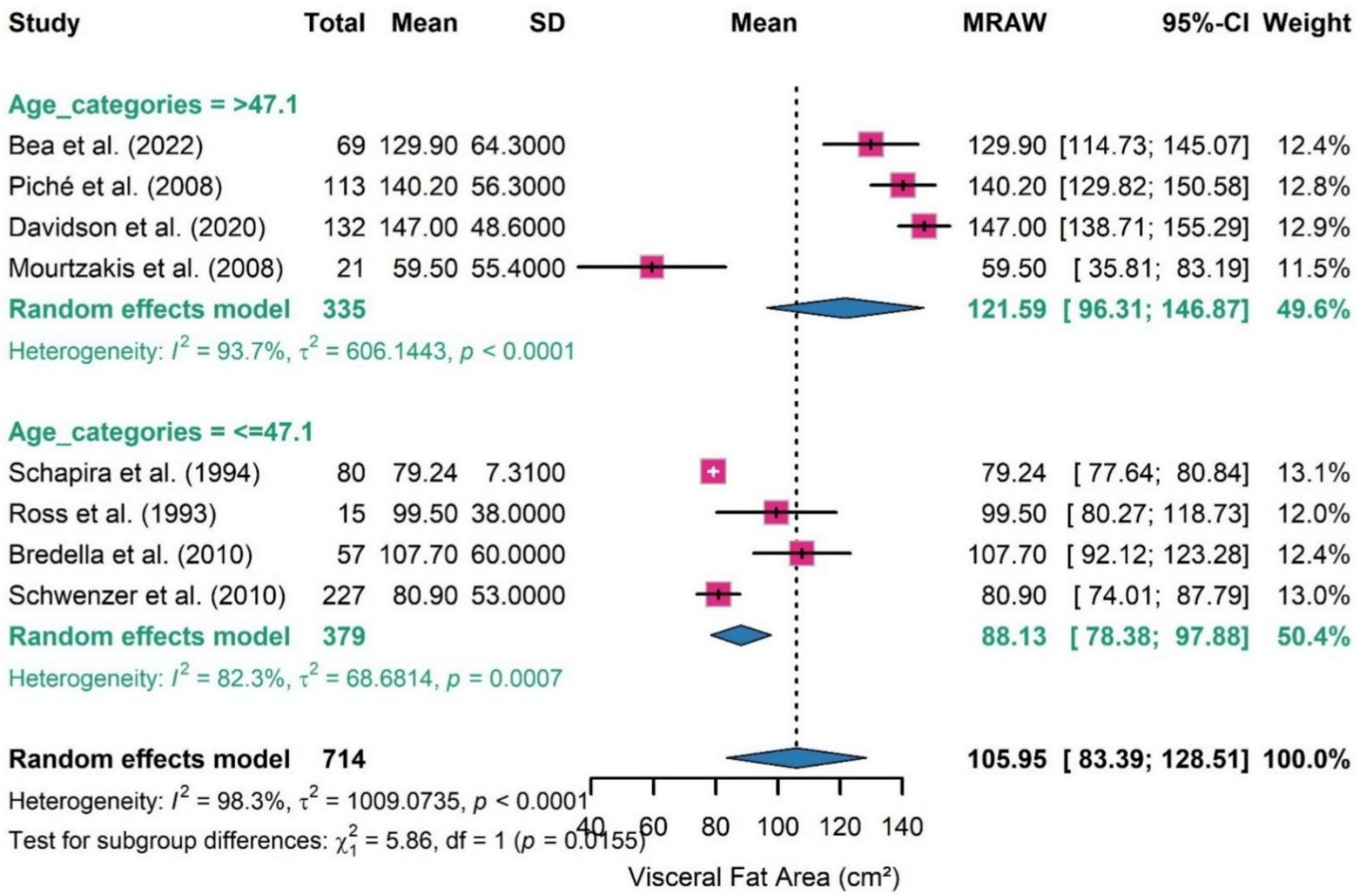

Fig. S 39: Forest plot of 8 studies (n=714) comparing regional (n=392) versus whole-body (n=322) assessment methods. Highly significant differences were found (p=0.002), with regional measurements yielding higher estimates (118.83 cm², 95% CI: 96.27-141.40; I²=93.3%) versus whole-body approaches (81.38 cm², 95% CI: 75.61-87.14; I²=54.7%). The substantially lower heterogeneity in whole-body measurements suggests this method may provide more consistent results for visceral fat quantification.

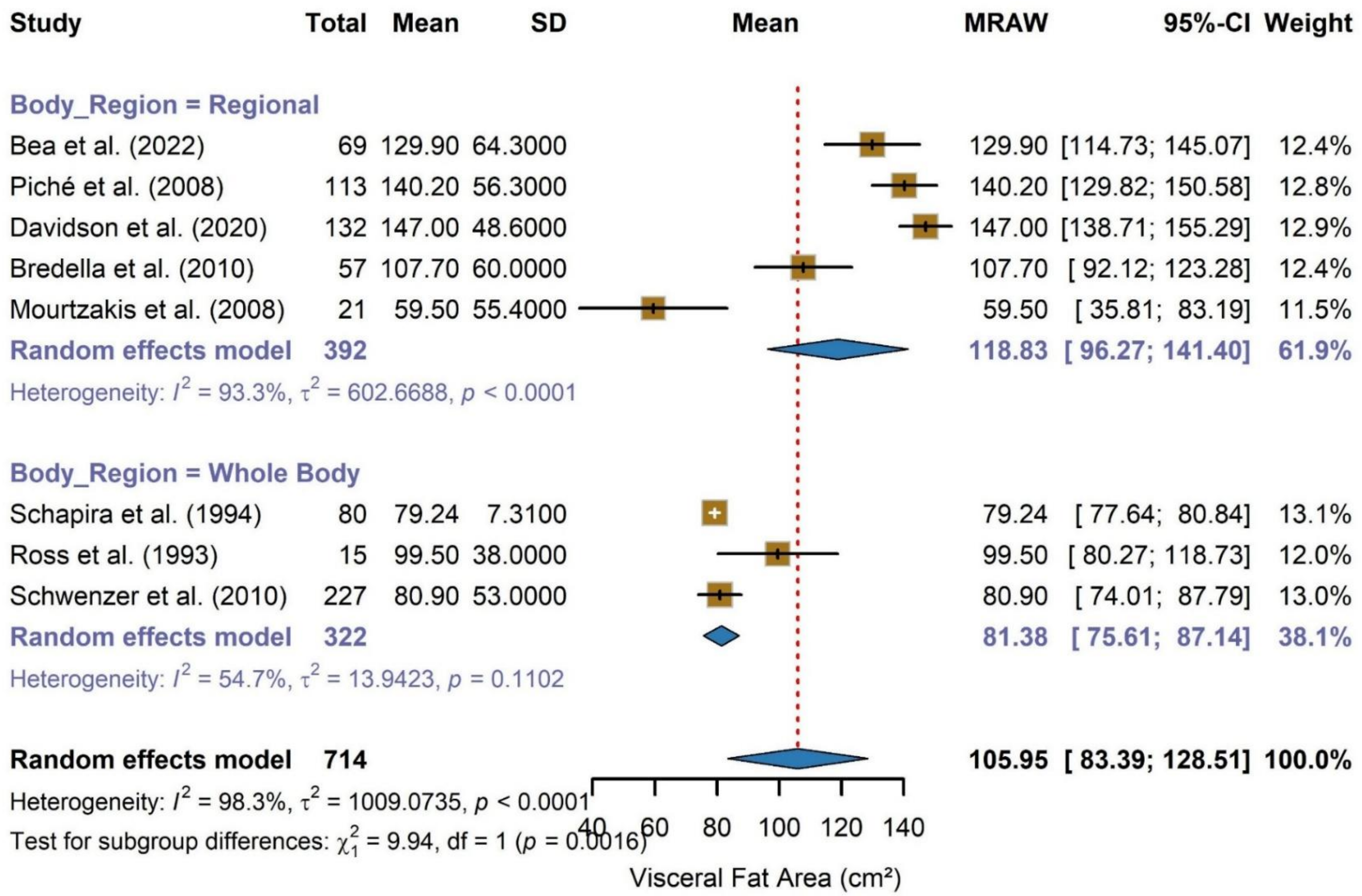

Fig. S 40: Forest plot evaluating comorbidity subgroups (No [n=556] vs Yes [n=158]) across 8 studies. Significant differences were found (p=0.001), with non-comorbid patients showing higher visceral fat area (119.60 cm², 95% CI: 88.30-150.91; I²=97.8%) versus comorbid patients (83.34 cm², 95% CI: 61.87-104.81; I²=87%). The extreme overall heterogeneity (I²=98.3%) suggests substantial variability in adiposity measurements between studies.

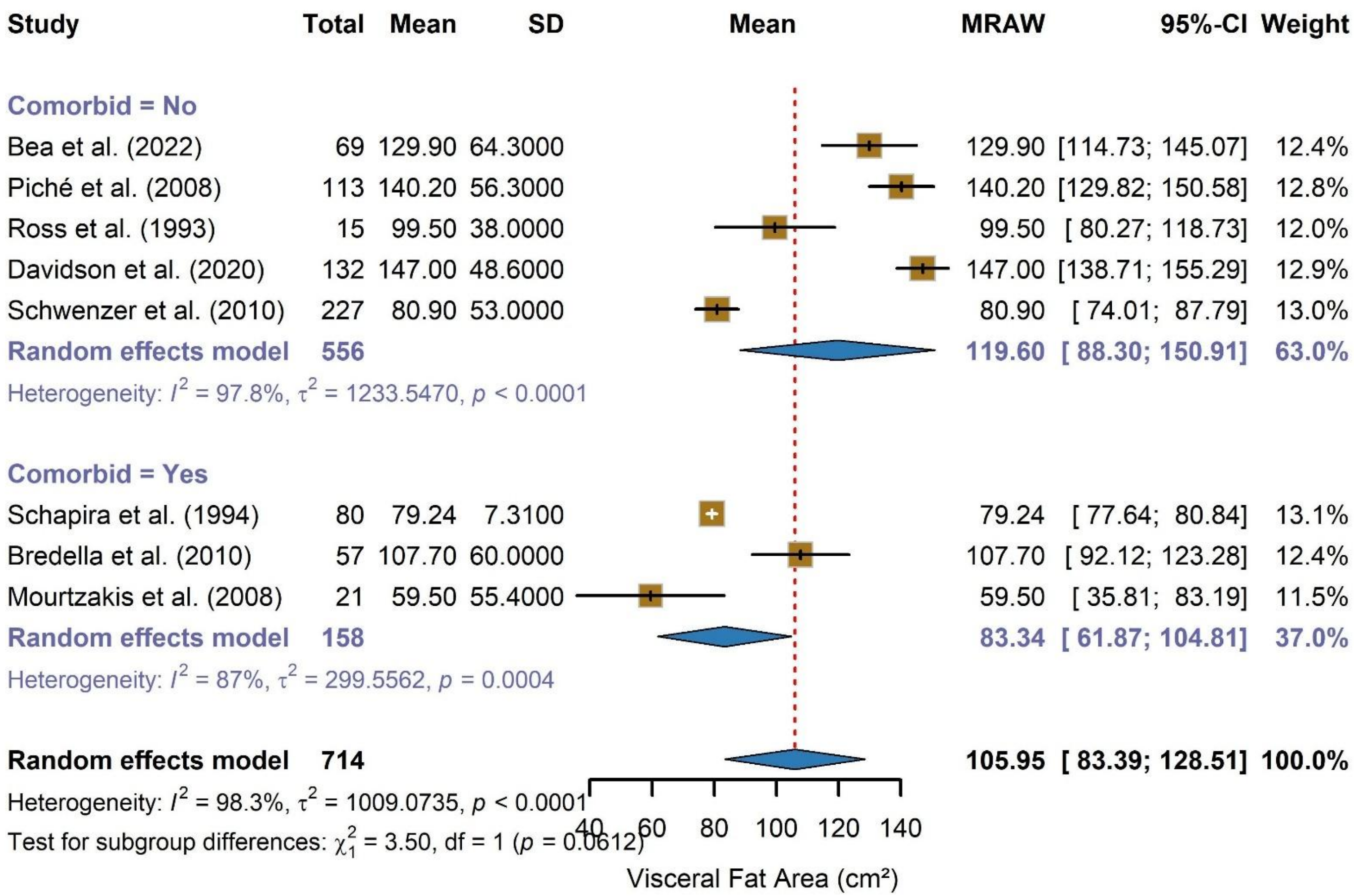

Fig. S 41: Forest plot comparing imaging modalities (DXA [n=258], CT [n=214], MRI [n=242]) across 8 studies. Significant differences were found (p=0.022), with CT showing the widest variability (59.50-140.20 cm²; I²=98.5%) and MRI demonstrating the lowest heterogeneity (I²=68.6%). DXA measurements ranged from 107.70-147.00 cm² (I²=90%), highlighting modality-specific measurement characteristics.

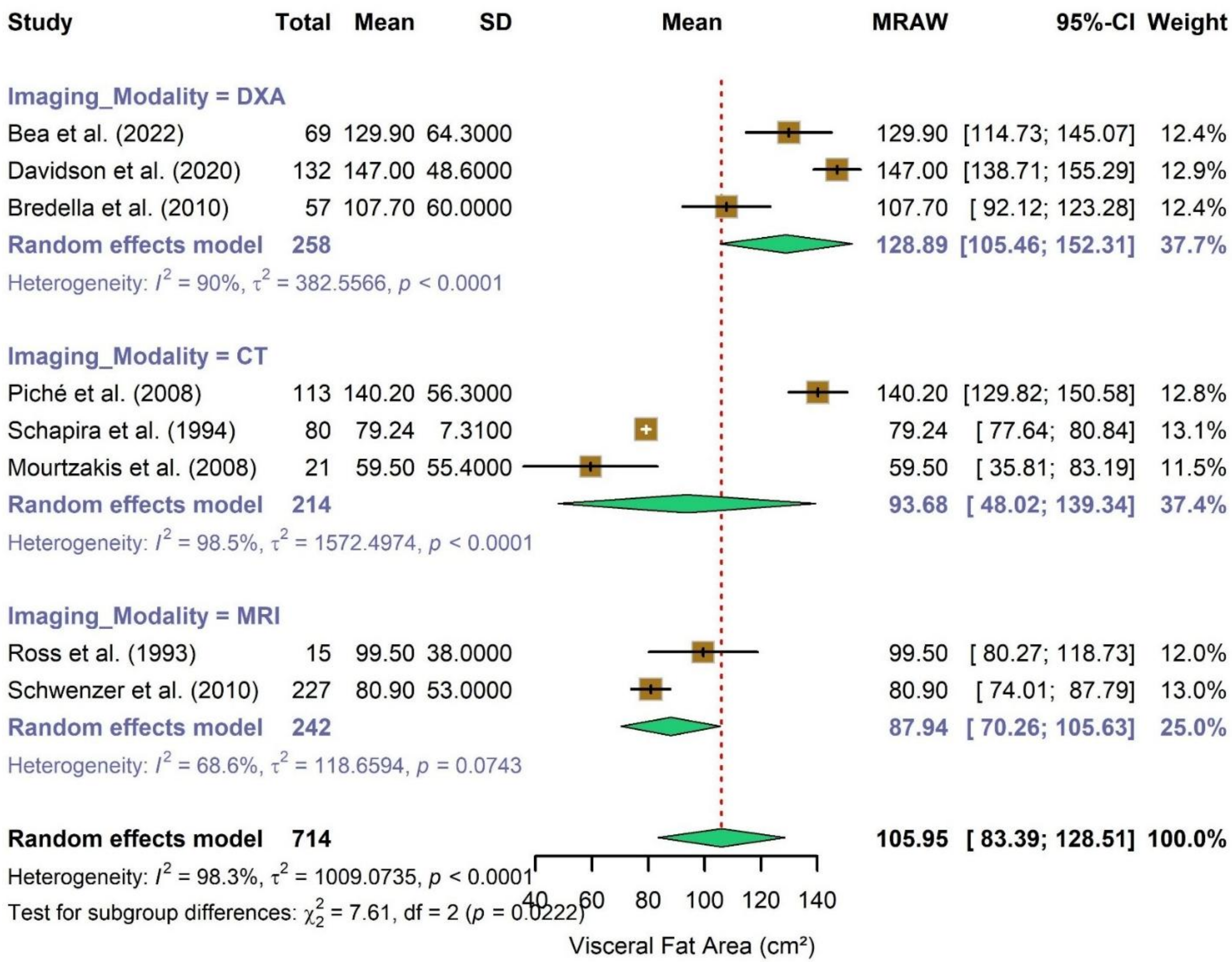

Fig. S 42: Sensitivity analysis of subcutaneous fat volume (n=13 studies) demonstrates robust pooled estimates (22,536 cm³, 95% CI: 12,804-32,268) maintained across all study omissions (range: 19,777-24,128 cm³). Extreme heterogeneity persists throughout all iterations (I^2=99.9%, τ^2=113,896,843), confirming the stability of findings despite substantial between-study variability in adiposity measurements.

Study	MRAW	95%-CI	P-value	Tau2	Tau	I2
Omitting Bea et al. (2022)	19777.01	[11509.73; 28044.29]	.	111949757.4625	10580.6312	99.9%
Omitting Hexsel et al. (2022)	24123.93	[14142.64; 34105.22]	.	102242708.5973	10111.5137	99.8%
Omitting Schautz et al. (2011)	21499.94	[11113.35; 31886.52]	.	112790019.0573	10620.2645	99.9%
Omitting Thomas et al. (1998)	20892.19	[10906.35; 30878.04]	.	78542494.5118	8862.4204	99.9%
Omitting Schapira et al. (1994)	23213.17	[12597.15; 33829.18]	.	191618964.7192	13842.6502	99.9%
Omitting Sohlstrom et al. (1993)	23921.98	[13761.72; 34082.24]	.	114542619.7647	10702.4586	99.9%
Omitting Fowler et al. (1991)	22072.61	[11452.04; 32693.18]	.	113532251.2460	10655.1514	99.9%
Omitting Davidson et al. (2020)	22391.30	[11707.71; 33074.89]	.	107123463.0601	10350.0465	99.9%
Omitting Bredella et al. (2010)	22193.00	[11536.65; 32849.36]	.	113067958.6883	10633.3418	99.9%
Omitting Linder et al. (2019)	23334.02	[12817.68; 33850.36]	.	114541072.6116	10702.3863	99.9%
Omitting Maislin et al. (2012)	23660.73	[13284.30; 34037.17]	.	136046145.7252	11663.8821	99.9%
Omitting Schwenzer et al. (2010)	21752.59	[11226.76; 32278.42]	.	110033744.8814	10489.6971	99.9%
Omitting Mourtzakis et al. (2008)	24127.70	[14127.77; 34127.62]	.	116237382.2347	10781.3442	99.9%
**Random effects model**	**22536.06**	**[12804.18; 32267.94]**	**.**	**113896842.9022**	**10672.2464**	**99.9%**

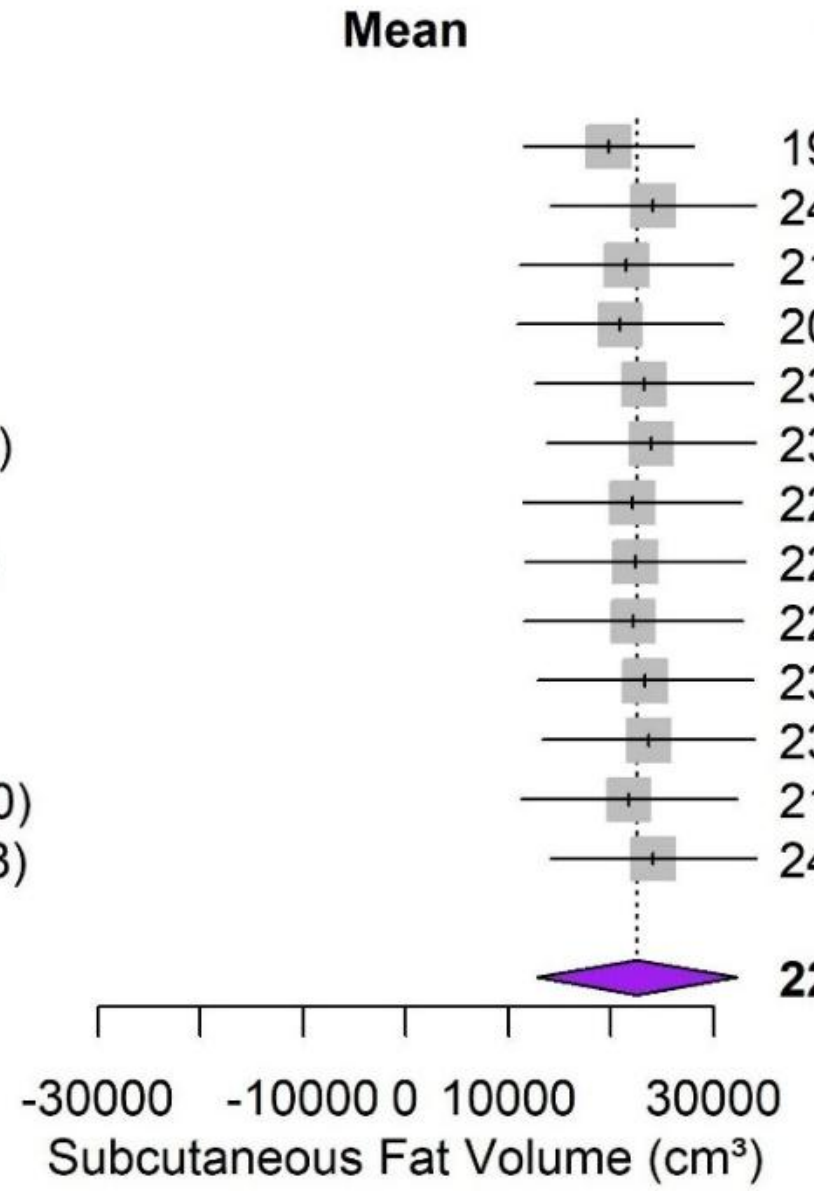

Fig. S 43: Sensitivity analysis of subcutaneous fat area (n=10 studies) shows consistent pooled estimates (312 cm², 95% CI: 195-430) across all exclusions (range: 289-340 cm²). The persistent extreme heterogeneity (I^2=100%) across all analyses underscores the need for standardized measurement protocols in body composition research.

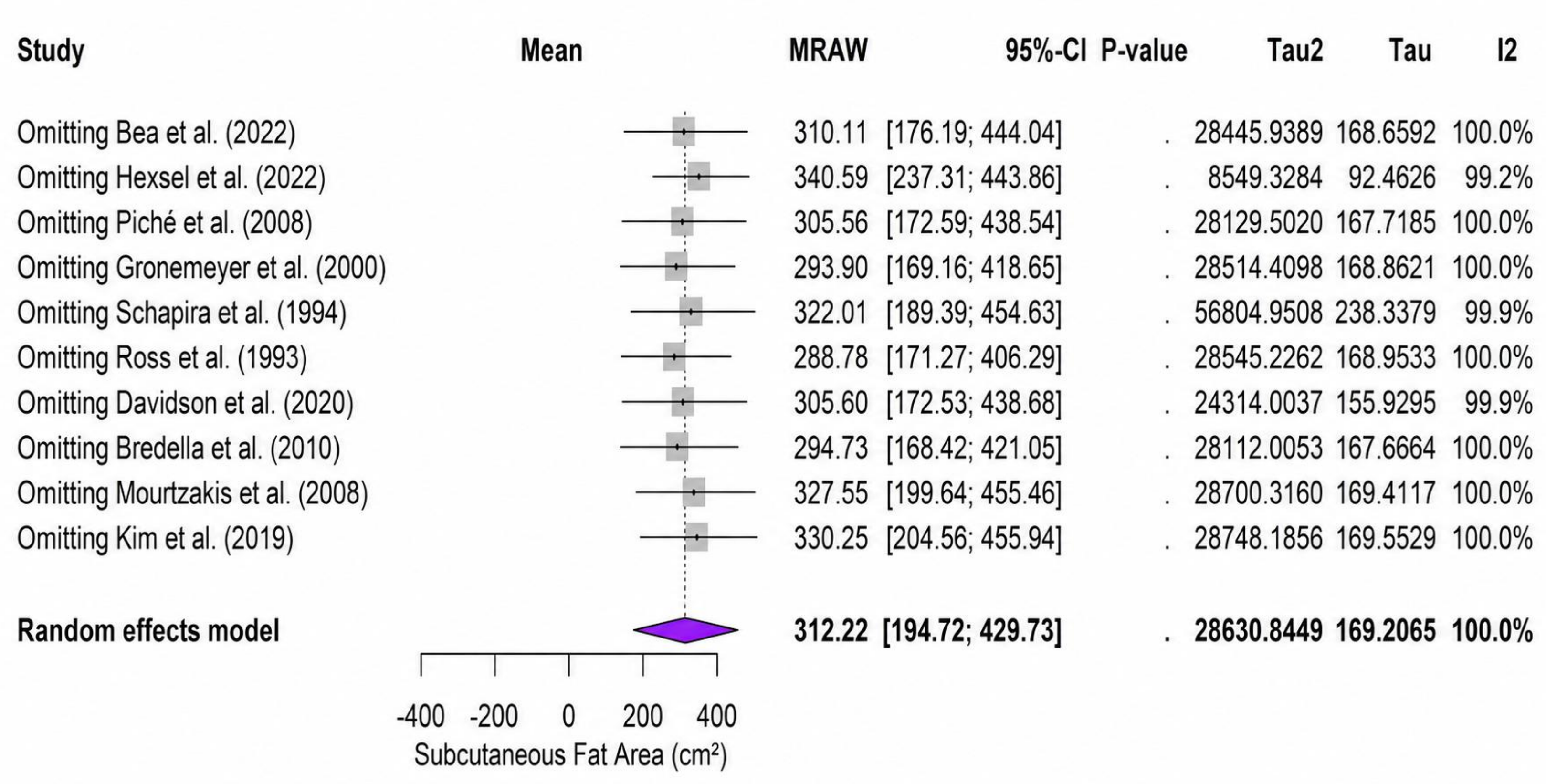

Fig. S 44: Sensitivity analysis of visceral fat volume (n=13 studies) reveals stable pooled estimates (3,615 cm³, 95% CI: 1,487-5,743) throughout all study omissions (range: 3,022-4,748 cm³). The universal extreme heterogeneity (I²=100%) highlights significant methodological variability in visceral adiposity quantification across studies.

Study	MRAW	95%-CI	P-value	Tau2	Tau	I2
Omitting Bea et al. (2022)	3467.40	[1504.57; 5430.23]	.	152349.2660	390.3194	100.0%
Omitting Froelich et al. (2020)	4747.78	[1743.76; 7751.81]	.	1301623.1568	1140.8870	100.0%
Omitting Jayawarde et al. (2020)	4751.90	[1737.13; 7766.68]	.	1326197.1460	1151.6063	100.0%
Omitting Schautz et al. (2011)	3776.24	[1450.99; 6101.50]	.	152423.4907	390.4145	100.0%
Omitting Thomas et al. (1998)	3021.59	[977.50; 5065.67]	.	112878.7455	335.9743	100.0%
Omitting Schapira et al. (1994)	3411.58	[1103.60; 5719.56]	.	134012.0682	366.0766	100.0%
Omitting Sohlstrom et al. (1993)	3475.56	[1172.49; 5778.63]	.	151912.8014	389.7599	100.0%
Omitting Armao et al. (2006)	3741.79	[1452.80; 6030.77]	.	152883.8693	391.0037	100.0%
Omitting Davidson et al. (2020)	3187.42	[1147.40; 5227.43]	.	150462.9437	387.8955	100.0%
Omitting Bredella et al. (2010)	3503.65	[1238.43; 5768.88]	.	152557.1021	390.5856	100.0%
Omitting Maislin et al. (2012)	3637.20	[1296.18; 5978.22]	.	152230.2921	390.1670	100.0%
Omitting Schwenzer et al. (2010)	3737.17	[1396.31; 6078.03]	.	149862.2742	387.1205	100.0%
Omitting Mourtzakis et al. (2008)	3661.02	[1486.02; 5836.02]	.	76745.3810	277.0296	100.0%
**Random effects model**	**3614.81**	**[1486.73; 5742.89]**	**.**	**152926.4153**	**391.0581**	**100.0%**

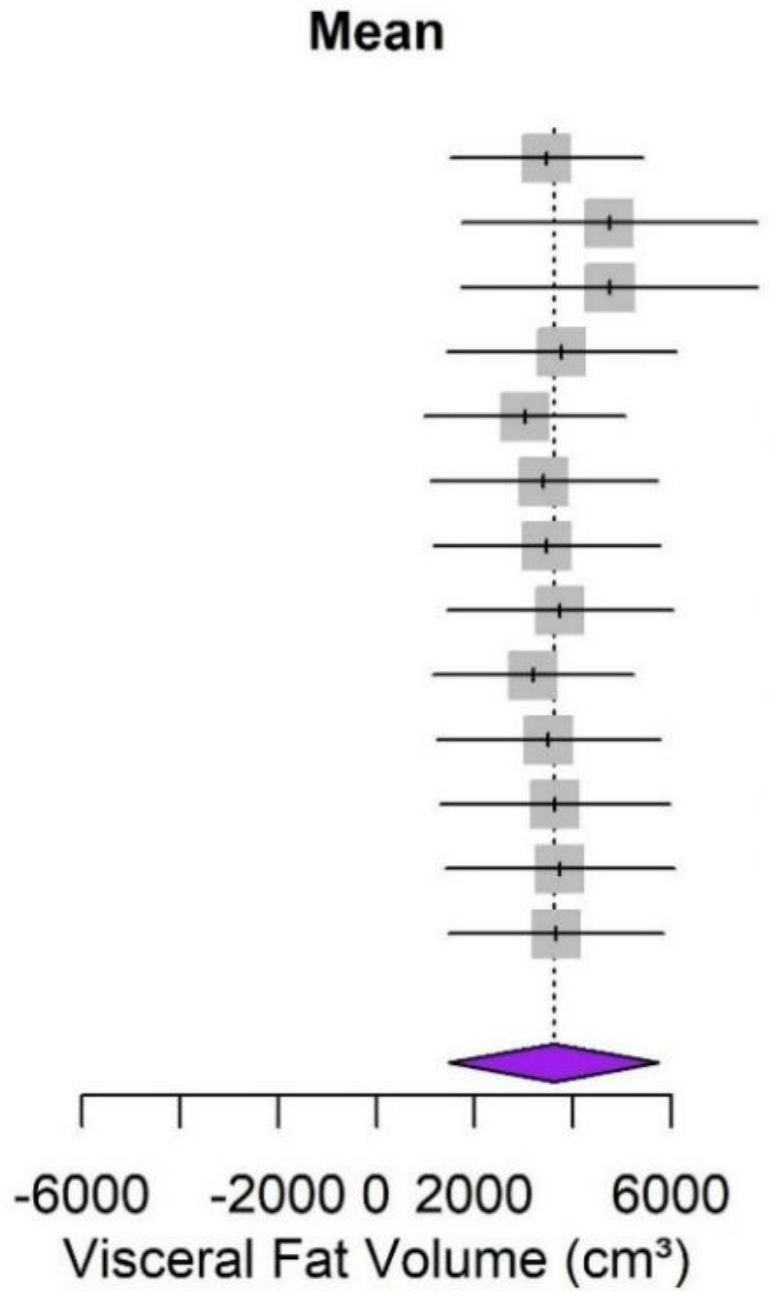

Fig. S 45: Forest plot evaluating visceral fat area estimates (n=10 studies). The analysis revealed remarkable stability (range: 86.52-104.01 cm²) around the pooled estimate of 92.69 cm² (95% CI: 61.36-124.01), with all confidence intervals overlapping. The exclusion of Froelich et al. (2020) notably reduced heterogeneity (I^2=98.1% vs 99.9% overall), suggesting this study may contribute disproportionately to between-study variability in visceral fat area measurements.

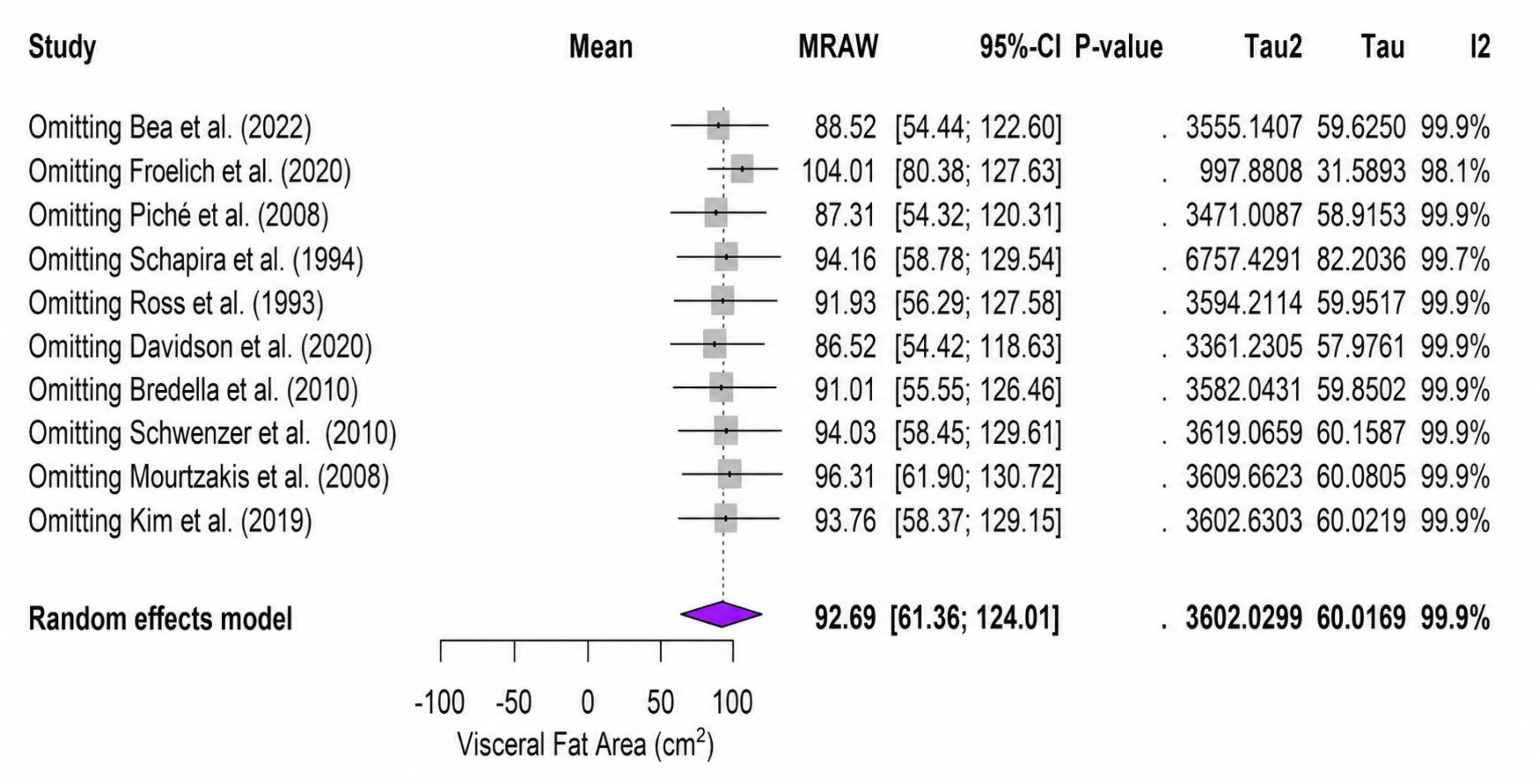

Fig. S 46: Forest plot demonstrating the robustness of total fat volume estimates (n=13 studies) when sequentially omitting individual studies. The pooled estimate remained stable (range: 30,072.65-37,760.37 cm³) despite extreme heterogeneity (I²≥99.6% in all iterations). The overall estimate of 35,062.32 cm³ (95% CI: 16,041.66-54,082.98) showed <15% variation when any single study was excluded, confirming the analysis was not unduly influenced by individual studies. The consistently high τ² values (>96 million across all iterations) highlight substantial between-study variability regardless of study exclusion.

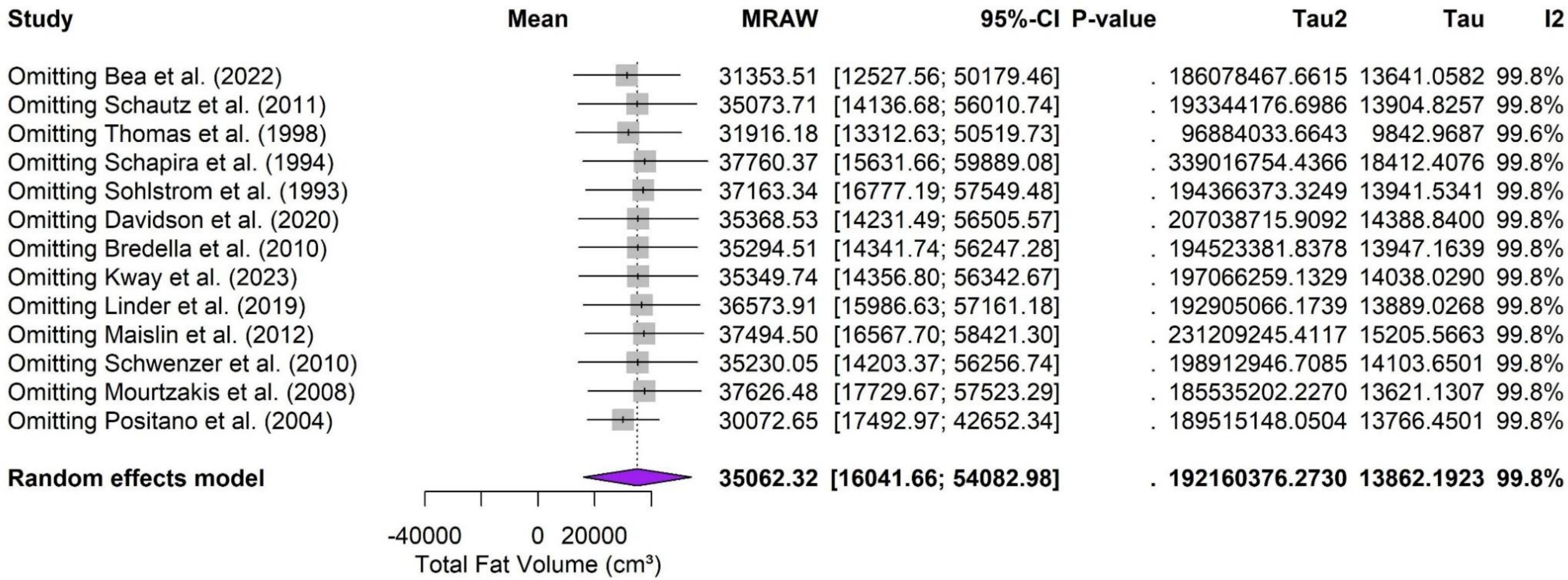

Fig. S 47: Forest plot assessing the stability of total fat area estimates (n=9 studies). The pooled estimates varied modestly (range: 387.25-434.25 cm^2) when excluding individual studies, consistently overlapping with the overall estimate of 411.12 cm^2 (95% CI: 289.90-532.35). Heterogeneity remained extremely high ($I^2 \geq 97.1\%$) in all iterations, though τ^2 values (14,664.27-17,970.58) were substantially lower than for fat volume analyses, suggesting greater relative consistency in area measurements.

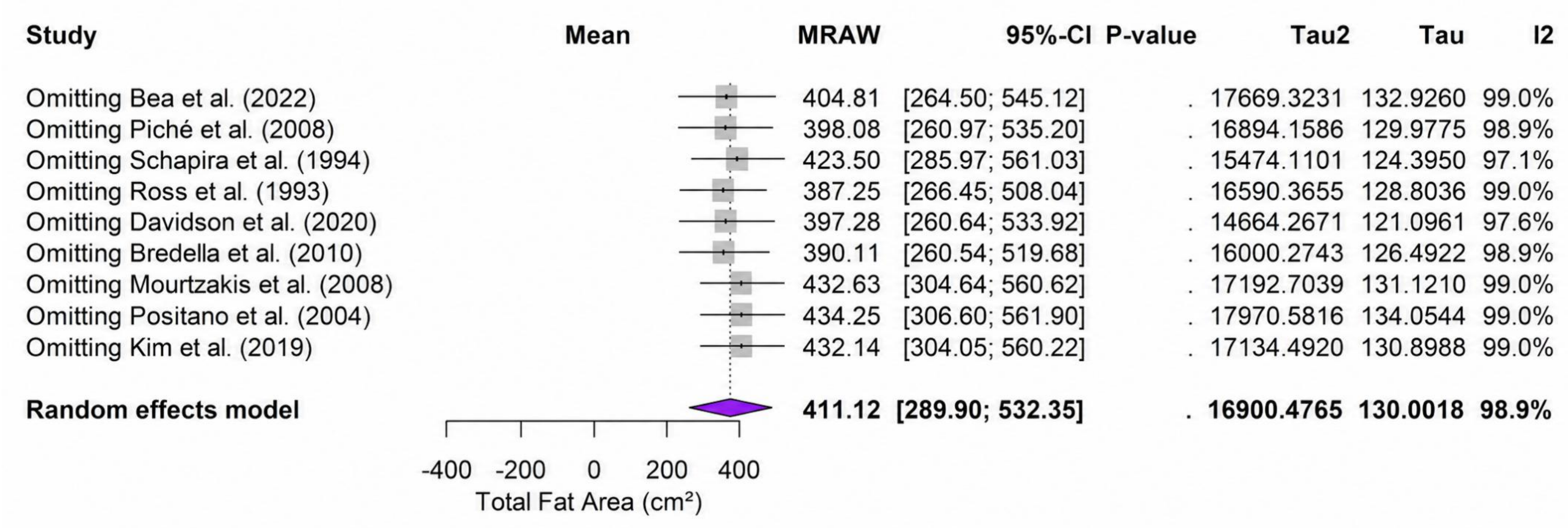

Table S 5: Quantitative evaluation using Egger's linear regression (p=0.032) and Begg's rank correlation (p=0.047) tests for adiposity metrics.

Fat Metric	Egger's Test			Begg's Test			Bias (SE)
	Test Statistic (t)	df	p-value	Bias (SE)	Test Statistic (z)	p-value	
Subcutaneous Fat Volume	2.14	11	0.055	27.53 (12.84)	-0.24	0.807	-4.00 (16.39)
Visceral Fat Volume	2.76	11	0.019*	53.06 (19.23)	-0.24	0.807	-4.00 (16.39)
Total Fat Volume	1.02	11	0.328	9.06 (8.84)	0.49	0.626	8.00 (16.39)
Subcutaneous Fat Area	2.07	8	0.073	29.87 (14.46)	-1.34	0.180	-15.00 (11.18)
Visceral Fat Area	2.67	8	0.028*	25.54 (9.57)	-2.06	0.040*	-23.00 (11.18)
Total Fat Area	1.40	7	0.205	5.31 (3.79)	-0.21	0.835	-2.00 (9.59)

Fig. S 48: Trim-and-fill adjusted funnel plot for subcutaneous fat volume (SFV) shows asymmetric distribution of effect sizes, particularly in the high standard error range (>2000). The plot indicates potential publication bias with missing studies likely in the low effect size (<20,000 cm³) and high variability quadrant, implying underrepresentation of smaller null studies.

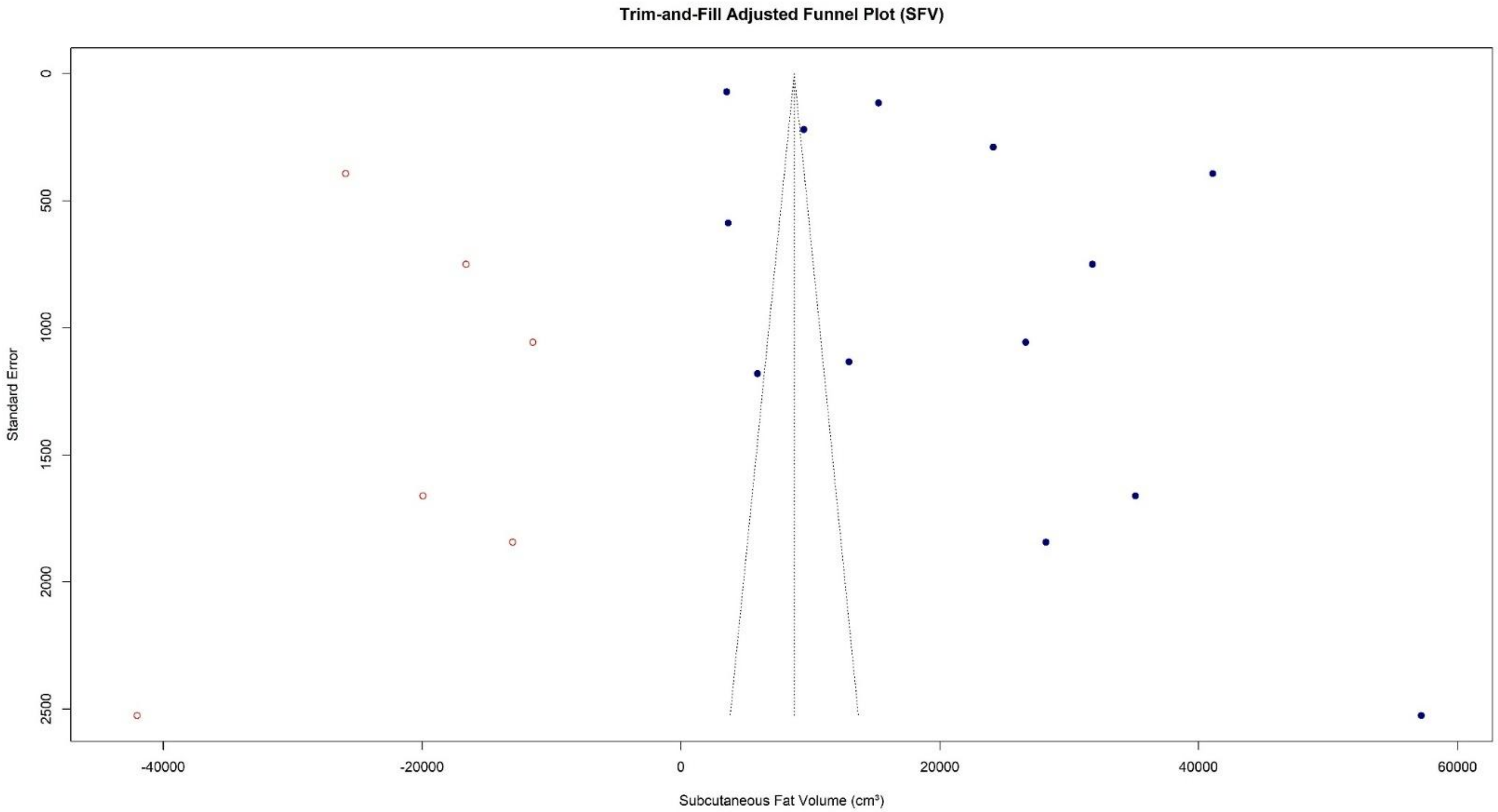

Fig. S 49: Trim-and-fill adjusted funnel plot for visceral fat area (VFA) demonstrates significant asymmetry across the full spectrum of spread error values (0-726), with imputed studies concentrated in high variability regions (>100 spread error). This pattern reveals substantial publication bias, suggesting systematic exclusion of studies with higher measurement variability from the literature.

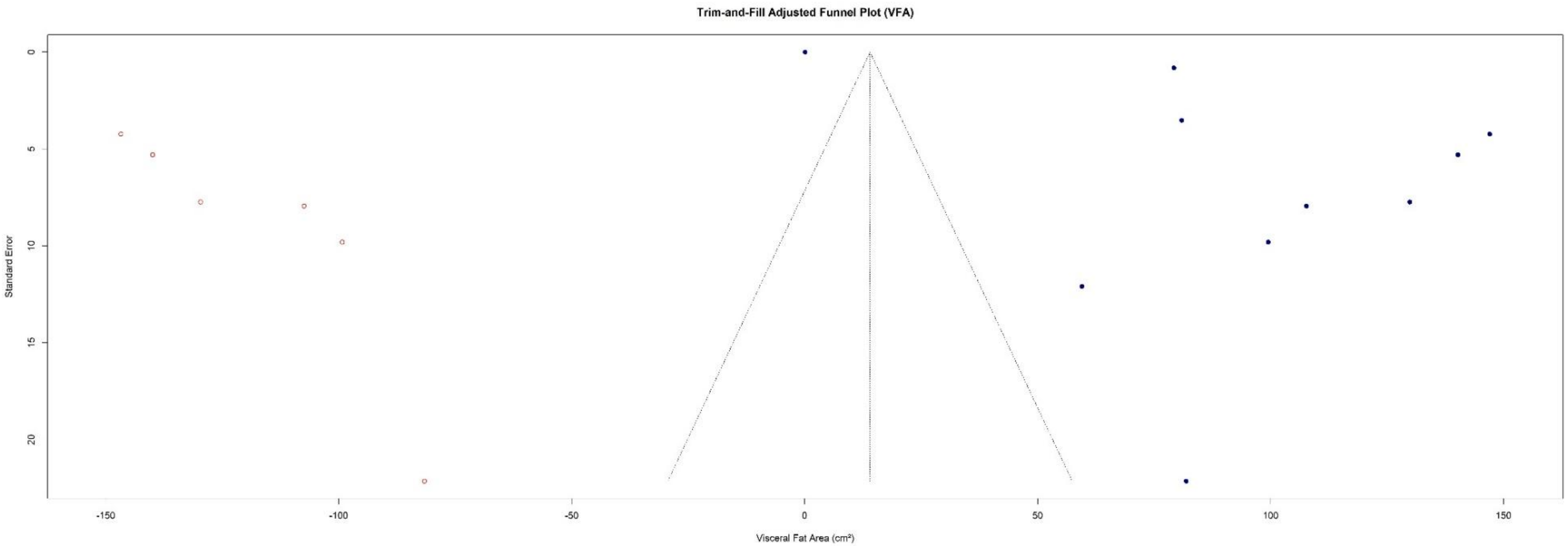

Supplement: Supplementary file 2 [file OBR-27-e70187-s002.pdf]
